# Asian dust-deposition flux to the subarctic Pacific estimated using single quartz particles

**DOI:** 10.1038/s41598-023-41201-6

**Published:** 2023-09-29

**Authors:** Kana Nagashima, Hajime Kawakami, Koji Sugie, Tetsuichi Fujiki, Jun Nishioka, Yoko Iwamoto, Toshihiko Takemura, Takuma Miyakawa, Fumikazu Taketani, Maki Noguchi Aita

**Affiliations:** 1 Research Institute for Global Change, Japan Agency for Marine-Earth Science and Technology, Yokosuka, Japan; 2 Research Institute for Value-Added-Information Generation, Japan Agency for Marine-Earth Science and Technology, Yokohama, Japan; 3 Institute of Low Temperature Science, Hokkaido University, Sapporo, Japan; 4 Graduate School of Integrated Sciences for Life, Hiroshima University, Higashi-Hiroshima, Japan; 5 Research Institute for Applied Mechanics, Kyushu University, Fukuoka, Japan

**Keywords:** Biogeochemistry, Environmental sciences, Ocean sciences

## Abstract

Iron availability limits marine ecosystem activities in large areas of the ocean. However, the sources and seasonal supply of iron, critically important for controlling surface ocean biogeochemistry and carbon cycling, are poorly understood. The western subarctic Pacific is a high-nutrient and low-chlorophyll region, and despite high concentrations of macronutrients, iron limits phytoplankton production in summer. Here, we determine the seasonal deposition flux of Asian dust using scanning electron microscope–cathodoluminescence analysis of single quartz particles derived from the western subarctic Pacific during 2003–2022 to trace provenance. We found a high (up to 6.9 mg m^−2^ day^−1^) deposition flux of Asian dust in May, June, and early July, with an annual average of 1.0 ± 0.2 mg m^−2^ day^−1^. The supply of dissolved-iron flux calculated from Asian dust was 0.9 ± 0.3 µg m^−2^ day^−1^ during the high productivity season (April–July), which is approximately half that from the deeper part of the ocean, calculated from vertical profiles of dissolved iron. Our study provides a reliable approach for estimating iron supply from dust to the surface ocean that may be critical for sustaining biological productivity under future ocean stratification, which suppresses nutrient supply from the subsurface ocean.

## Introduction

Iron availability limits primary production in large areas (approximately 30%) of the surface ocean^1^. The western subarctic Pacific is a high-nutrient and low-chlorophyll area, where the availability of iron limits phytoplankton production in summer^2,3^. Even under such a limited supply of micronutrient iron, this area has a capacity for significant biological CO_2_ drawdown^4^. The mechanism and amount of bioavailable iron supplied to this region are therefore critical for the North Pacific’s marine ecosystems and the biogeochemical cycling of carbon.

Four potential sources of iron in the subarctic Pacific have been established: (1) Iron is derived from mineral dust emitted from the deserts in China and Mongolia, transported eastward through the westerlies, and deposited into the sea^5^. (2) Iron from anthropogenic aerosols from East Asia is transported eastward through the westerlies^6,7^. (3) Iron is supplied from volcanic ash from the Kuril and Aleutian Islands^8–11^. (4) Iron enriched in the intermediate water originating from the north-western shelf of the Okhotsk Sea is transported to the surface by advection or diffusion or retained in the mixed layer by deep winter mixing^12,13^.

Asian dust has been considered a major source of iron supplied to the western subarctic Pacific^14,15^. However, recent comprehensive trace element measurements revealed the vertical and spatial distribution of dissolved iron in the North Pacific and highlighted the importance of sedimentary iron supply via intermediate water circulation^3,12^. These findings demonstrate the need for a thorough description of the ocean surface phytoplankton production system that quantifies iron supplied via the atmosphere as well as ocean circulation.

Dust deposition fluxes into the ocean estimated using dissolved aluminum concentrations and thorium isotopes from mixed layers show large uncertainties^16,17^ (e.g., 0.015–0.6 g m^−2^ year^−1^ in the subarctic gyre^16^) because their solubilities and residence times are not well understood. In addition, it is difficult to distinguish between dissolved elements from dust and those from volcanic or coastal sources. To solve these problems and evaluate deposition flux, we focused on quartz as the tracer of Asian dust, as detrital quartz is one of the major components of dust^18^ and is chemically stable during transport. We performed scanning electron microscope–cathodoluminescence (SEM-CL) analysis of single quartz particles in shallow seawater to estimate the dust deposition flux. SEM-CL can detect impurities and native imperfections in quartz that vary with conditions during its formation and subsequent geological background (e.g., metamorphic pressure and temperature)^19^, thus characterising quartz particles from Asian deserts^20^. Based on the identified quartz particles from Asian deserts within the surface mixed layer of the western subarctic Pacific, we calculated the seasonal dust deposition flux into the ocean. Furthermore, we estimated the dissolved-iron flux supplied by the Asian dust and assessed its relative importance for phytoplankton production by comparing it to the dissolved-iron flux from the deeper ocean.

### Provenance of quartz particles in the surface mixed layer of the subarctic Pacific

**Figure 1:**
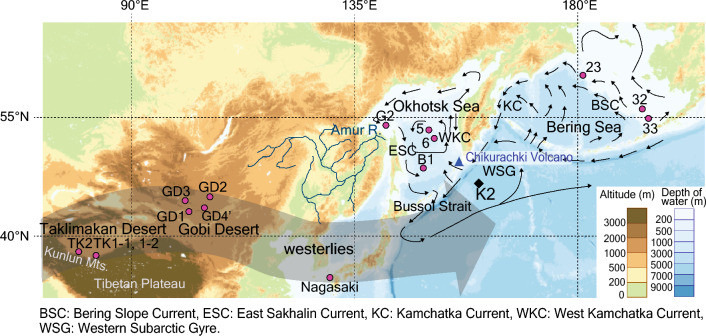
Locations of station K2 and other terrestrial and marine sampling stations. The black diamond and pink circles show K2 and other terrestrial and marine sampling stations, respectively. Thin black arrows show the main surface-water currents^57,58^. We modified this map from the Geospatial Information Authority of Japan (https://maps.gsi.go.jp/).

We measured CL spectra of quartz particles in samples representing their potential sources in the western subarctic Pacific such as Asian deserts, the Bering Sea, and the Okhotsk Sea (Fig. 1, Supplementary Table S1). Volcanic eruptions from the Kuril and Aleutian Islands are other sources of quartz transported to the subarctic Pacific, and we used published CL features of volcanic quartz^19^ to represent them.

**Figure 2:**
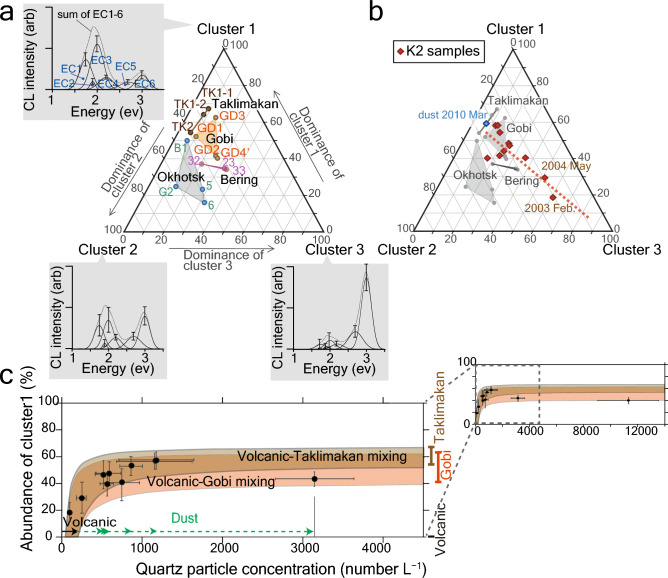
Provenances of quartz particles in seawater at station K2. (**a**) Cluster compositions of quartz particles in samples from Gobi and Taklimakan Deserts and Bering and Okhotsk Seas. The relative abundances of emission components (EC1–EC6) for each cluster are plotted close to each corner point. (**b**) Cluster compositions of quartz particles in K2 samples. The regression line of K2 samples is also shown as a red dotted line. (**c**) Diagram showing the relationship between abundance of cluster 1 and quartz particle concentration. The curves illustrate the mixing lines between volcanic quartz and quartz from Taklimakan Desert (light brown curve) or Gobi Desert (orange curve). Error bars show 95% confidence intervals.

We separated each measured CL spectrum into six Gaussian emission components (EC1–EC6), each of which reflects an individual impurity or native imperfection in the quartz particle^21^ (Supplementary Fig. S1, Table S2). Then, we performed a hierarchical cluster analysis, utilising the calculated fractional areas of EC1–EC6 for quartz particles from the potential sources (Supplementary Table S3). The calculated cluster analysis dendrogram contained clusters 1–3, characterised by high abundances of EC1 and EC3, EC3 and EC6, and EC5 and EC6, respectively (Fig. 2a and Supplementary Fig. S2, Table S4).

The characteristics of these clusters correlated well with those identified by Nagashima et al*.*^20^ using Asian desert samples that overlap with those of the present study. Based on empirical relations between the CL features of quartz particles and those of host-rock types^19,22–24^, Nagashima et al*.*^20^ interpreted “cluster 1” as characteristic of quartz particles in low-grade metamorphic or slow-cooling high-grade metamorphic rocks, whereas “cluster 2” and “cluster 3” were characteristic of quartz particles in plutonic rocks and volcanic/fast-cooling high-grade metamorphic rocks, respectively. Clusters 1–3 identified by us are similar to their “clusters 1–3”^20^.

Cluster 1 predominated in the three loess samples from Kunlun Mountains (TK1-1, TK1-2, and TK2, Figs. 1, 2a, Supplementary Table S5), representing the Taklimakan Desert, and in the four Gobi Desert samples (GD1–4’), which is consistent with the widely exposed low-grade metamorphic rocks surrounding the desert regions^20^. The Bering Sea samples (23, 32, and 33) are characterised by similar abundances of clusters 1–3 (Fig. 2a). In contrast, four samples from the Okhotsk Sea (5, 6, B1, and G1) showed a primary abundance of cluster 2, consistent with the broad exposure of plutonic rocks along the Amur River basin^25,26^ draining to the Bering Sea (Fig. 1).

We then performed SEM-CL analysis of a dust sample collected in western Japan (Nagasaki, Fig. 1, Supplementary Table S1) in March 2010 and filtered seawater samples derived from depths of 10 and 20 m at station K2 (47° 00′ N, 160° 00′ E, in the North Pacific western subarctic gyre, Fig. 1) during 2003–2022 (Supplementary Table S1).

The CL spectra of quartz particles in K2 and dust samples showed EC1–EC6 compositions similar to clusters 1–3. We classified each particle into one of these clusters by calculating the sum of squared differences between the EC1–EC6 fractional areas of the CL spectrum for each quartz particle and those of clusters 1–3 (Supplementary Table S4) and then selecting the cluster with the minimum difference (Supplementary Table S6).

The dust sample exhibits a cluster composition close to that of the Asian deserts (Fig. 2b), which confirms that the CL analysis is useful for identifying Asian dust. The samples from K2 show a primary abundance of cluster 1, except for February 2003 and May 2004, in which cluster 3 is dominant (Fig. 2b). K2 samples generally lie around a line connecting the corner point of cluster 3 and the Gobi and/or Taklimakan Deserts, suggesting the mixture of at most three endmembers. Among the endmembers, cluster 3 represents volcanic quartz, possibly from the Kuril Islands, because the high abundance of blue luminescence—equivalent to EC5 and EC6—is characteristic of volcanic quartz^19^. In support of this interpretation, at least 10 eruptions of Chikurachki Volcano, an active stratovolcano located in the Northern Kurile Islands Chain^27^ (Fig. 1), have been reported after 2002 (https://volcano.si.edu). The eruptions are not synchronous to K2 samples, suggesting the lateral transport of volcanic materials within seawater rather than direct input from the atmosphere.

To check the mixing of at most three sources (the volcano and the Gobi and Taklimakan Deserts), we compared the abundance of cluster 1 with concentrations of quartz particles in seawater, determined using SEM-energy dispersive X-ray spectroscopy (EDS). Figure 2c shows the mixing line between volcanic quartz (cluster 1 abundance of 0% and particle concentrations of 50–200 L^−1^) and quartz from the Gobi or Taklimakan Desert (cluster 1 abundances of 41–62% and 54–67% (Supplementary Table S5), respectively, with variable particle concentrations).

Suitable fits to the logarithmic curves thus support the idea that quartz particles from K2 samples are a mixture of quartz from the Gobi and Taklimakan Deserts and of volcanic origin. In contrast, the contribution of volcanic quartz seems to be minor, with particle concentrations of less than 200 L^−1^ (solid black arrow in Fig. 2c). According to this interpretation, most K2 samples, excluding those of February 2003 and May 2004, are composed predominantly of Asian dust.

### Dust concentrations, deposition fluxes, and their seasonality

**Figure 3:**
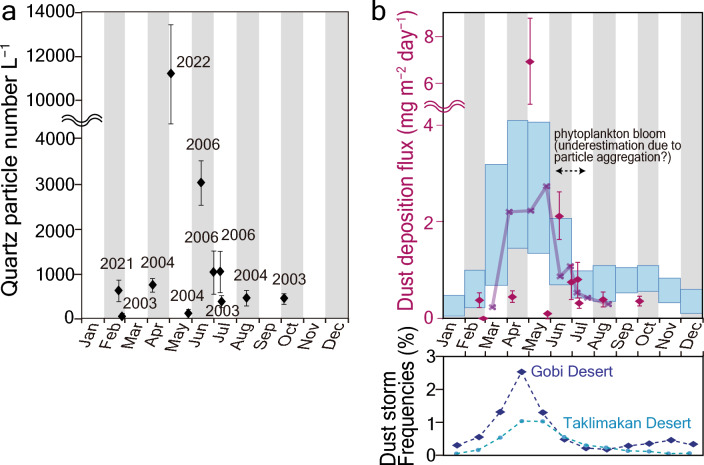
Seasonal variations in quartz particle concentrations originating from Asian deserts and dust-deposition flux at station K2. (**a**) Monthly changes in quartz particle concentrations from Asian deserts. (**b**) The deposition flux of Asian dust (pink diamonds) obtained in this study. Thick purple line shows three points moving average of the neighbouring dust deposition flux data ordered by month. Light-blue bars represent the monthly mean of dust deposition flux averaged during 2001–2020 (average ± σ of the 20-year variability, shown on a daily basis in the left column) as simulated using SPRINTARS^37–39^. The bottom panel shows the seasonality of dust storm frequencies defined as the percentage of reported dust storm occurrences among total observations at surface meteorological stations in the Gobi and Taklimakan Deserts averaged during 1998–2004^28^.

We calculated concentrations of quartz particles of dust origin for K2 samples (green dotted arrows in Fig. 2c) by extracting the estimated concentrations of volcanic quartz particles (50–200 particles L^−1^) and sorting them by month (Fig. 3a). The concentrations were highest in May 2022 (~ 11,000 particles L^−1^), which is consistent with the spring peak of dust-storm frequencies in the Asian deserts^28^ (Fig. 3b), followed by mid-June 2006 (~ 3000 particles L^−1^). Samples from late June 2006 and early July 2006 consisted of ~ 1000 particles L^−1^, and those in samples from April 2004, February 2021, August 2004, October 2003, and mid-July 2003 exceeded 400 particles L^−1^. Therefore, Asian dust is commonly supplied to the subarctic Pacific from spring to autumn.

We calculated the seasonal deposition flux of Asian dust at station K2 using quartz particle concentrations in seawater and the daily settling depth of particles. We calculated the settling velocity of the particles using Stokes’ law^29^ for each particle size (*De*) class ranging from 0.7 to 20 µm (− 0.2 < log_10_ *De* < 1.3; Supplementary Fig. S3), which we classified into 15 diameter classes at every Δlog_10_ *De* = 0.1. We applied the law to quartz particles because other factors influencing the settling speed of particles, in particular particle aggregation (as observed in the subtropical North Atlantic with high deposition of Sahara dust^30^) and particles involved in faecal pellets, are expected to be minor at station K2 during most of the seasons. This is because (1) dust deposition flux on the western subarctic Pacific is up to 10 times lower than that on the subtropical North Atlantic according to the results of multiple numerical simulations^31^, resulting in a lower ability to form aggregates; (2) aggregation rate related to dust deposition was reported to increase from the surface to the depth of deep chlorophyll max^30^ (ca. 60 m in summer at station K2)^32^, and therefore, the aggregation rate seems to be lesser at our sampling depths (10 and 20 m); and (3) the rate of particles integrated into faecal pellets of mesozooplankton may be small because of the low abundance of salps, and the *Neocalanus* species abundant at station K2 prefer to feed on larger particles (> 5 µm) compared to the most dust-particle sizes (< 5 µm) observed in this study (Supplementary Fig. S3)^33^. The exception is the phytoplankton bloom period in June and July at station K2^34–36^, when settling speed may increase following particle coagulation with phytoplankton and organic materials.

The calculated deposition fluxes of Asian dust had high values of 6.9 mg m^−2^ day^−1^ in early May, 2.1 mg m^−2^ day^−1^ in early June, and 0.3 mg m^−2^ day^−1^ in July (Fig. 3b, Table S7). A large variation was observed within the same month, e.g., from 0.1 to 6.9 mg m^−2^ day^−1^ in May. Such variations may reflect whether the sample was able to capture dust events. The profile of the running mean better represents the seasonal trend by averaging the cases with and without dust events (Fig. 3b). We compared seasonality to the dust frequency record in Asian deserts. The most significant peak in spring, followed by a decrease in summer, is a common feature in our dust deposition profile and dust storm frequency in the Gobi and Taklimakan Deserts (Fig. 3b)^28^, suggesting that the seasonality of dust deposition flux is primarily governed by the dust emission frequency in the source regions. Note that both profiles show the monthly halving trends from May to July. The similar decreases toward summer suggest that aggregation by phytoplankton bloom in summer may not cause a substantial underestimation of dust deposition fluxes.

We then compared our estimated values and the seasonality to the simulated monthly deposition flux of Asian dust at station K2 using the Model for Interdisciplinary Research on Climate coupled with the Spectral Radiation-Transport Model for Aerosol Species (MIROC-SPRINTARS, Fig. 3b, Supplementary Table S8, https://sprintars.riam.kyushu-u.ac.jp/archive.html)^37–39^. The seasonality we found is very similar to that of MIROC-SPRINTARS simulations (Fig. 3b). The overall average deposition flux was 1.0 ± 0.2 mg m^−2^ day^−1^ (0.37 ± 0.07 g m^−2^ year^−1^; n = 11, Supplementary Table S7), which is close to that of MIROC-SPRINTARS simulations, determined using monthly data from 2001 to 2020 (0.41 ± 0.08 g m^−2^ year^−1^, Supplementary Table S8). In addition, the estimated Asian dust deposition is slightly lower but consistent with the dust-deposition flux of 0.58 ± 0.15 g m^−2^ year^−1^, which was estimated based on the atmospheric dust concentration data at Shemya (52° 44′ N, 174° 06′ E, ca. 1200 km northeast from K2) in the Aleutian Islands using the scavenging ratio model^40^. Consequently, independent estimations of dust deposition fluxes using quartz particles in the upper mixed layer, numerical simulation, and atmospheric dust concentration in the Aleutian Islands result in ca. 0.4 g m^−2^ year^−1^, suggesting the reliability of such estimates.

### Dissolved iron supply from Asian dust and other potential sources

**Figure 4:**
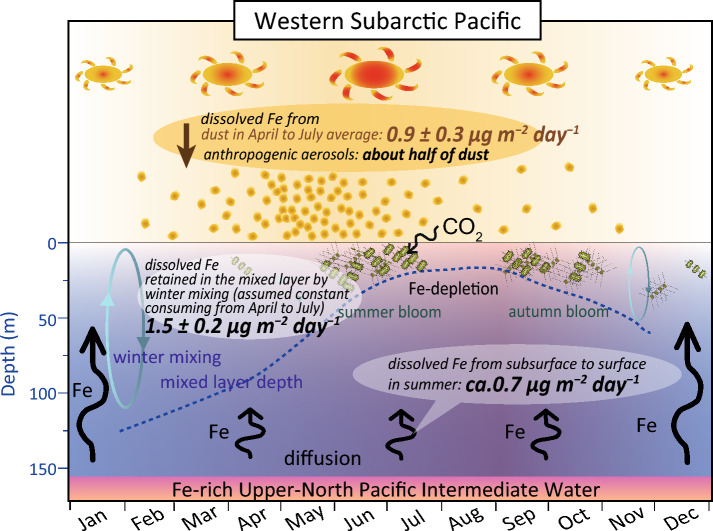
Seasonality of the iron supply from Asian dust and other potential sources, phytoplankton production, and other oceanic environments. This schematic illustration denotes the seasonality of phytoplankton production and its relation to the depth of mixed layer, ocean temperature, insolation, and dissolved iron supply from Asian dust, anthropogenic aerosol, and the intermediate water. The dissolved iron flux supplied from each potential source in spring and summer is also shown. Seasonality of phytoplankton bloom and depth of mixed layer are adapted from Refs.^34–36^.

We assessed the impact of seasonal dust deposition on phytoplankton production and the relative importance of dissolved iron supply from Asian dust by comparing the flux to those from other potential sources. The phytoplankton summer bloom at station K2 appears in early summer under increased light availability, high temperature, and a shallow mixed layer^34^ and lasts for 1–2 weeks until the phytoplankton exhaust the iron in the mixed layer^34–36^ (Fig. 4). The deposition flux of Asian dust exhibits a high value before and during the period of the phytoplankton summer bloom and then decreases until the bloom ends (Figs. 3b, 4). Such seasonality of dust supply illustrates its potential impact on the scale and duration of the phytoplankton summer bloom.

We estimated the flux of dissolved iron deposited at station K2 to examine the relative importance of dissolved iron supply from Asian dust and other sources. We used an iron content of 5.3% ± 0.3% within Asian dust, as determined from multiple Asian dust samples collected in Korea^41^. Iron solubility in Asian dusts is the other critical component for this estimate, which varies from ca. < 1 to 20%^42–44^, mainly depending on particle size^44^ and possible contamination of anthropogenic aerosols in the aerosol samples used for solubility measurements^7,45^. We assumed an iron solubility of conservative 1.1 ± 0.4%, based on iron solubilities for a pH 4.5 buffer solution resembling wet deposition using the > 2.5 µm (aerodynamic diameter) fraction of aerosol samples (n = 16) collected at the subtropical North Pacific during a dust event^43^. These values eliminate the effects of anthropogenic aerosols because such aerosols are concentrated in the finer (< 2.5 µm) fraction^7^. Using such values, the seasonal dissolved iron supplied by Asian dust is 0.05–4.0 µg m^−2^ day^−1^ (Table S7).

To evaluate the abundance of bioavailable iron supplied from Asian dust relative to that from other potential sources, such as intermediate water and anthropogenic aerosols, we calculated those fluxes for April to July, covering the phytoplankton bloom and the preceding period. Volcanic ash also contributes dissolved iron supply to the subarctic Pacific^8–11^, but our CL data do not support the direct atmospheric deposition of volcanic ash during our sampling periods. The dissolved-iron flux from Asian dust averaged from April to July was 0.9 ± 0.3 µg m^−2^ day^−1^. In contrast, approximately 0.7 µg m^−2^ day^−1^ of dissolved iron was supplied from the intermediate water to the ocean surface by eddy diffusion and vertical advection at station K2 in summer calculated from the vertical profile of dissolved iron (Table S9, Supplementary Information). In addition, iron from the intermediate water is retained in the mixed layer by deep winter mixing^12^ and consumed by phytoplankton production during the following season. The amount was ca. 1.5 ± 0.2 µg m^−2^ day^−1^, as calculated by the seasonal mixed layer depth and vertical profile of dissolved iron at station K2 in summer^12^, assuming constant consumption from April to July (Supplementary Information, Table S9).

Consequently, the iron supply via intermediate water circulation is estimated to be approximately two times larger than that from the Asian dust. However, there is a possibility that the difference becomes smaller when we precisely evaluate the underestimation of summer dust deposition flux due to particle aggregation. The amount of soluble iron supplied by anthropogenic aerosols in summer was estimated to be approximately half of that supplied by Asian dust according to the iron isotope values of the marine aerosols in this region^7^. According to the estimations, dissolved iron input from the atmosphere (Asian dust plus anthropogenic aerosols) is about two-thirds of that from the intermediate water; thus, both dissolved iron sources appear to be essential for phytoplankton production in the western subarctic Pacific.

The fate of iron from Asian dust after deposition needs more study into aspects such as the conditional stability of iron-binding organic ligands that determine the solubility and bioavailability of dissolved iron^46^. Accelerated stratification associated with recent and future surface ocean warming reduces nutrient supply from the deeper part of the ocean to the euphotic zone^47^. In such cases, iron supply from the atmosphere will increase in importance. Therefore, future changes in the supply of dust and anthropogenic aerosols are important issues that require attention.

The present study demonstrates that physicochemically stable quartz particles are useful tracers of dust transport and deposition processes and are important means of quantitative evaluation. The SEM-CL analysis on single quartz particles in particular is a novel approach for identifying Asian dust from volcanic ash and coastal sources, which had not been distinguished before^16,17^. Since quartz is also a major component of dust in other deserts, such as the Sahara^48^, our method has the potential to calculate precise dust-deposition fluxes to the world’s oceans. However, further fundamental observations are needed to evaluate how particle aggregation affects particle sinking speeds in the upper mixed layer for broader applications.

## Methods

### Sample collection

We collected in situ filtration samples from depths of 10 and 20 m at station K2 during seven cruises in 2003, 2004, and 2006 with filtration quantities of 7–30 L (Supplementary Table S1). We performed in situ filtration using pumping systems (McLane Inc. Large Volume Pump WTS-6-1-142 V) containing glass-fibre filters with pore sizes of 0.7 μm. In addition, we collected 1–12 L of water samples at station K2 from depths of 10 and 20 m using Niskin bottle samplers during cruises in 2021 and 2022, respectively (Supplementary Table S1). These water samples were filtered using Supor membrane disc filters with a pore size of 0.45 μm.

For comparison, we also collected samples representing the potential sources of Asian deserts, the Bering Sea, and the Okhotsk Sea (Supplementary Table S1). As the reference for Asian dust, we collected four samples from the Gobi Desert in Mongolia and three mountain loess samples from the northern foot of the Kunlun Mountains. Nagashima et al*.*^20^ also used the same samples for CL analysis, but we measured them again because we used an improved SEM-CL system in this study.

As the reference for quartz particles from the Okhotsk Sea, we collected two in situ filtration samples using pumping systems (McLane Inc. Large Volume Pump WTS-6-1-142 V) containing Nucleopore filters with pore sizes of 1.0 μm at stations close to the mouth of the Amur River (station G2) and in the southern part of the sea (station B1)^49^ (Fig. 1, Supplementary Table S1). These samples were obtained from the salinity range of 26.7–27.0 σθ^49^ that is characteristic of the dense shelf water of the north-western continental shelf^50^, which is the source of the southward-floating Okhotsk Sea Intermediate Water entering the subarctic Pacific^50^. In addition, we used a bucket to collect two filtered surface-water (0 m) samples from stations 5 and 6 in the central part of the Okhotsk Sea^51^ (Fig. 1). We also collected three filtered surface-water (0 m) samples from stations 23, 32, and 33 on the eastern continental shelf of the Bering Sea^51^ (Fig. 1) as the reference for quartz particles from this sea. These filtrations were conducted using Nuclepore filters with a pore size of 0.4 μm. The influence of different pore sizes (from 0.4 to 1.0 μm) of filters used for filtration on the results of SEM-CL measurements was small because we used quartz particles mostly larger than 1.0 μm both from potential sources and station K2.

Dust samples were collected on a ADVANTEC PF-040 Teflon filter using a High-volume virtual-impactor air sampler (Kimoto Electric Co., Ltd., AS-9), which was installed at Nagasaki University, Nagasaki, Japan, during a huge dust event during 20–21 March, 2010.

### Pre-treatment for SEM-CL analysis

The particles on filter samples were washed off from the filter to the 70-cc plastic tube by subjecting to ultrasonic waves for a maximum of 60 s with ca. 10 cc of water, then treated with 10% hydrogen peroxide solution to remove organic matter and 2 mol L^−1^ of sodium carbonate solution to remove opal to condense mineral particles^52,53^. Filtration of residuals were conducted again with isopore membrane filters with a pore size of 0.4 μm (φ = 13 mm, filtration area of 12 mm^2^). These filters were dried and pasted on aluminium disks and coated with a ~ 2-nm carbon film to prevent charge build-up on the surface during electron irradiation.

The mineral particles of desert samples were embedded in a small dimple (φ 2 mm) on a brass disk with non-luminescent epoxy resin and were polished and mirror-finished using 1-μm diamond powder. The prepared samples were also coated with ~ 2-nm carbon.

### SEM-CL measurement

Preceding the SEM-CL analysis, we identified quartz particles using an SEM (FEI Quanta 450 FEG) equipped for EDS (EDAX, Octane Elite 30) under operating conditions of accelerating voltage of 15 kV, spot size of 5, and collection time of 10 s/particle.

We performed SEM-CL analyses by improving the method of Nagashima et al*.*^20^. The measurements were conducted for 44–447 quartz particles per sample identified by SEM–EDS analysis. The numbers of particles used for the measurements were adequate to identify Asian dust particles (Supporting Information, Fig. S5). We measured CL spectra from 350 to 830 nm in 3.3-nm steps using an SEM combined with a CL system (Gatan, Mono CL4 Swift). The CL spectra were excited using a continuous electron beam at normal incidence, with accelerating voltage of 15 keV at room temperature, spot size of 5, and irradiated area of 0.1–4 μm^2^ depending on the particle size of quartz, and collected using a paraboloidal mirror collector. A grating monochromator dispersed the CL, using settings of 150 l/mm, blazed 500 nm, a focal length of 0.3 m, F of 4.2, a limit of 0.5 nm resolution, and a slit width of 1 nm at the inlet and 3 nm at the outlet. The dispersed CL was recorded by a charge-coupled device (CCD) camera. As the CCD camera could detect the CL intensity at multiple wavelengths at the same time, the measurement time in this study (2–5 s per particle) was much shorter than the time required (8 min per particle) in Nagashima et al*.*^20^, in which they used a photomultiplier tube to detect CL spectra. The reduced measurement times minimise changes in the CL spectrum during a measurement, which frequently happens for quartz particles smaller than ca. 5 μm (Figs. S2 and S3 of Nagashima et al.^20^).

After making the measurements, we corrected all spectra for the total instrumental response using the response correction curve provided by Gatan Inc. and presented the results in energy units (eV).

### CL data analysis

To identify the type and intensity of emission components shown as Gaussian curves in energy units^21^, we separated each CL spectrum into six Gaussian emission components (EC1–EC6), respectively centred at 1.75, 1.9, 2.0, 2.2, 2.7, and 2.9–3.2 eV (Supplementary Fig. S1, Table S2). The position of the centre of EC6 was not fixed because its precise nature is yet to be resolved. These ECs are the same as those identified in Nagashima et al.^20^, except for EC4, which we newly identified to decrease the fitting residuals (Supplementary Fig. S1). We then calculated the fractional areas of EC1–EC6 for all the measured CL spectra. We performed a hierarchical cluster analysis based on Ward’s method^54^ using the calculated fractional areas of EC1–EC6 for quartz particles from the potential sources (Asian deserts and Bering and Okhotsk Seas) using Origin 2015 software. Finally, we calculated the relative compositions of the clusters identified for each sample based on the number of quartz particles constituting each cluster (Fig. 2, Supplementary Table S5). Such clustering and following processes were also previously reported^20^.

### Estimation of diameters and concentrations of quartz particles

We measured the equivalent diameter (*De*) of each quartz particle for K2 samples, defined as the diameter of a circle with the equivalent area, by calculating the particle area from the SEM image using ImageJ software (https://imagej.nih.gov/ij/, 1997–2018).

We counted quartz particle numbers for 10–50 randomly selected areas (each 0.024 mm^2^) in each filter, equivalent to 2–10% of the filter area (12 mm^2^). The total quartz particle number among selected areas was then converted to the number equivalent to each filter area. The calculated quartz-particle number per filter was used to calculate quartz particle concentration within seawater (number/L) divided by the filtered water amount.

The 95% confidence interval of quartz particle concentration was ± 16–40% of the value, varying depending on the unevenly-distributed quartz particles on the filter and total measurement area used for quartz particle counting.

### Calculation of deposition flux of Asian dust

We calculated the accumulated volume of quartz particles contained in the daily settling depth of seawater, then converted the volume into weight (density of quartz = 2.65 g cm^−3^)^55^. We considered the weight to represent the daily deposition flux of quartz in Asian dust. Assuming the quartz content within the Asian dust at 21 ± 4 weight%^41^, we calculated the deposition flux of Asian dust using the following equation:

1 $$\mathrm{Asian\, dust}-\mathrm{deposition\, flux} \left(\mathrm{mg}{\mathrm{ m}}^{-2} {\mathrm{day}}^{-1}\right)=\frac{2.65 \times {10}^{-6}}{21}\times QC \times \sum_{i = 1}^{15}\left(Ri\times Vi \times \frac{4}{3}\pi {(\frac{Di}{2})}^{3}\right),$$

where *i* indexes each *De* class, *QC* is the concentration of quartz originating from dust in seawater (particle number L^−1^), *Ri* is the relative ratio of particle numbers in *De* class *i* to all particles, *Vi* is the settling velocity (cm day^−1^), and *Di* (µm) is the log-scale centre diameter of *De* class *i*. We calculated *QC* by subtracting 50–200 particles L^−1^ (estimated particle numbers of volcanic quartz, Fig. 2a) from each sample’s total number of quartz particles. The particle number ratio and settling velocity for each *De* class is detailed in the following subsections.

### Calculation of particle number ratio for each particle diameter class

First, we calculated the count frequency distribution for each *De* using the *De* of all quartz particles in K2 samples from 10 and 20 m depths (Supplementary Fig. S3), excluding two samples that had a larger contribution of volcanic quartz (samples from February 2003 and May 2004, Fig. 2b). A frequency distribution was calculated for each diameter class (log_10_ *De* ranges between − 0.2 and 1.3, and 15 diameter classes were determined every Δlog_10_ *De* = 0.1). We smoothed the distributions of these samples by fitting them to two Gaussian distributions to eliminate the extreme distribution tailedness caused by large *De* classes (log_10_ *De* > 0.9) with frequencies of less than 1% (Supplementary Fig. S4).

### Calculation of settling velocity of quartz particles

We calculated the settling velocity *Vi* (cm day^−1^) of the quartz particles in the seawater of station K2 for each *De* class *i* based on Stokes’ law, using the following equation modified after Lamb^29^.

$$Vi=3600\times {24\times 10}^{-8}\frac{({\rho }_{p}- {\rho }_{f})}{ 18\mu } g {Di}^{2}$$

where *g* is the gravitational acceleration (980 cm s^−2^), *Di* (µm) is the log-scale centre diameter of *De* class *i, ρ*_*p*_ and *ρ*_*f*_ are the mass densities of the quartz particle (2.65 g cm^−3^) and seawater (1.025 g cm^−3^), respectively, and μ (g cm^−1^ s^−1^) is the dynamic viscosity of seawater. We calculated the dynamic viscosity of seawater by adopting a previously described equation^56^ with the sea surface temperature at station K2 of each season^36^: 0.0128 g cm^−1^ s^−1^ in August, 0.0135 g cm^−1^ s^−1^ in October, 0.0139 g cm^−1^ s^−1^ in July, 0.0147 g cm^−1^ s^−1^ in June, 0.0166 g cm^−1^ s^−1^ in May, and 0.0177 g cm^−1^ s^−1^ in February and April.

### Supplementary Information

Supplementary Information.

## Data Availability

All CL datasets are available in Supplementary Tables S3 and S6. Dust-deposition flux and dissolved iron flux calculated for K2 samples are available in Supplementary Table S7. Simulated dust-deposition flux is available in Supplementary Table S8.
