# Supplementary material for: Asian dust-deposition flux to the subarctic Pacific estimated using single quartz particles
Source: Sci Rep. 2023 Sep 29;13:15424. doi: 10.1038/s41598-023-41201-6 (PMC10541437; doi:10.1038/s41598-023-41201-6)
Supplement: Supplementary file 1 — Supplementary Information. [file 41598_2023_41201_MOESM1_ESM.pdf]

Supporting Information for

Asian dust-deposition flux to the subarctic Pacific estimated using single quartz particles

Kana Nagashima[1]*, Hajime Kawakami[2], Koji Sugie[1], Tetsuichi Fujiki[1], Jun Nishioka[3], Yoko Iwamoto[4], Toshihiko Takemura[5], Takuma Miyakawa[1], Fumikazu Taketani[1], Maki Noguchi Aita[1]

[1] Research Institute for Global Change, Japan Agency for Marine-Earth Science and Technology, Yokosuka, Japan

[2] Research Institute for Value-Added-Information Generation, Japan Agency for Marine-Earth Science and Technology, Yokohama, Japan

[3] Institute of Low Temperature Science, Hokkaido University, Sapporo, Japan

[4] Graduate School of Integrated Sciences for Life, Hiroshima University, Higashi-Hiroshima, Japan

[5] Research Institute for Applied Mechanics, Kyushu University, Fukuoka, Japan

Contents of this file

Representativeness of the limited particle numbers used for analyses

Estimation of dissolved iron flux transported upward from the subsurface ocean

Supplementary references

Figures S1–S5

Tables S1–S9

Representativeness of the limited particle numbers used for analyses

We tested the representativeness of the small number (ca. 40–440) of particles for the large population of particles. We calculated theoretical confidence intervals using the numbers of quartz particles (n = 10, 40, 100, 200, 400, and 1000) for the cluster composition assumed for Asian deserts as follows: cluster 1 = 60%, cluster 2 = 25%, cluster 3 = 15%.

The calculated ranges of confidence intervals decrease with the number of quartz particles (Fig. S5). When n = 40, the 68% (95%) confidence interval is 51–66% (44–74%) for cluster 1, 18–31% (11–38%) for cluster 2, and 8–20% (4–26%) for cluster 3. When n = 400, the 68% (95%) confidence interval is 57–62% (55–65%) for cluster 1, 23–27% (21–29%) for cluster 2, and 13–17% (12–19%) for cluster 3. Similarly, the 95% confidence intervals were calculated for all samples considering the particle number of each sample used for SEM–CL analysis (Table S5). The intervals are sufficient to discuss the contribution of Asian dust to K2 samples based on particle concentration data (Fig. 2c). Therefore, our measurements using a limited number of particles could represent a large population of particles within the uncertainties acceptable in this study.

Estimation of dissolved iron flux transported upward from the subsurface ocean

We estimated the dissolved iron flux transported upward from the subsurface ocean to the surface waters by vertical advection and eddy diffusion using the dataset of vertical concentrations of dissolved iron at station K2 during summer cruises (KH-12-4)[1]. We used simple one-dimensional models for the calculations, as follows[2,3]:

$$\text{Upward dissolved Fe flux by advection (ug m}^{-2}\text{ day}^{-1}) = \text{W} \times \text{R} \times 55.85$$

$$\text{Upward dissolved Fe flux by diffusion (ug m}^{-2}\text{ day}^{-1}) = \text{Kz} \times \text{dFe/dZ} \times 55.85$$

where W is the vertical velocity (here we adapted a value of 0.012 m day^{-1})[3], R is the mean concentration of dissolved iron in the vertical gradient in the subsurface layer (μmol m^{-3}), Kz is the coefficient of eddy diffusive (here, we adapted a value of 2 m^2 day^{-1})[4], dFe/dZ is the gradient in dissolved iron with depth (μmol m^{-4}), and 55.85 is the atomic weight of iron to convert from molar to gram units.

We also calculated the inventory of dissolved iron raised to the surface waters by winter mixing using the following equation[2] based on the isopycnal mixing model[5].

$$\text{Quantity of dissolved iron transported by winter mixing (ug m}^{-2}\text{)}$$

$$= (\text{C1} - \text{C2}) \times \text{D1} \times 55.85$$

where C1 is the summer dissolved iron concentration at the maximum depth of the winter mixed layer (μmol m^{-3}), C2 is dissolved iron concentration in the summer mixed layer (μmol m^{-3}), and D1 is the depth (m) of the summer mixed layer. Assuming that such iron retained in the mixed layer is consumed by phytoplankton production from spring to mid-summer, we divided the dissolved iron quantities by 120 days (April to July) and calculated the averaged daily supply of dissolved-iron related to the winter mixing. All the parameters for the calculations and the estimation results are summarized in Table S9.

References

1. Nishioka, J. & Obata, H. Dissolved iron distribution in the western and central subarctic Pacific: HNLC water formation and biogeochemical processes. *Limnol. Oceanogr.* **62**, 2004–2022 (2017).
2. Nishioka, J. et al. Iron supply to the western subarctic Pacific: Importance of iron export from the Sea of Okhotsk. *J. Geophys. Res. Oceans* **112**, C10012, https://doi.org/10.1029/2006JC004055 (2007).
3. Martin, J. H., Gordon, R. M., Fitzwater, S. & Broenkow, W. W. VERTEX: phytoplankton/iron studies in the Gulf of Alaska, *Deep Sea Res. Part A* **36***,* 649–680 (1989).
4. Nishioka, J. et al. Subpolar marginal seas fuel the North Pacific through the intermediate water at the termination of the global ocean circulation. *Proc. Natl. Acad. Sci. USA* **117**, 12665–12673 (2020).
5. Sarmiento, J. L., Thiele, G., Key, R. M. & Moore, W. S. Oxygen and nitrate new production and remineralization in the North Atlantic subtropical gyre. *J. Geophys. Res. Oceans* **95**, 18,303–18,315 (1990).
6. Nagashima, K. *et al*. Composition of Asian dust from cathodoluminescence spectral analysis of single quartz grains. *Geology* **45**, 879–882 (2017).

7. Stevens-Kalceff, M. A. Cathodoluminescence microcharacterization of point defects in α-quartz. *Mineral. Mag.* **73**, 585–605 (2009).

8. Götze, J., Plötze, M. & Habermann, D. Origin, spectral characteristics and practical applications of the cathodoluminescence (CL) of quartz—A review. *Mineral Petrol.* **71**, 225–250 (2001).

9. Fujiki, T. et al. Seasonal cycle of phytoplankton community structure and photophysiological state in the western subarctic gyre of the North Pacific. *Limnol. Oceanogr.* **59**, 887–900 (2014).

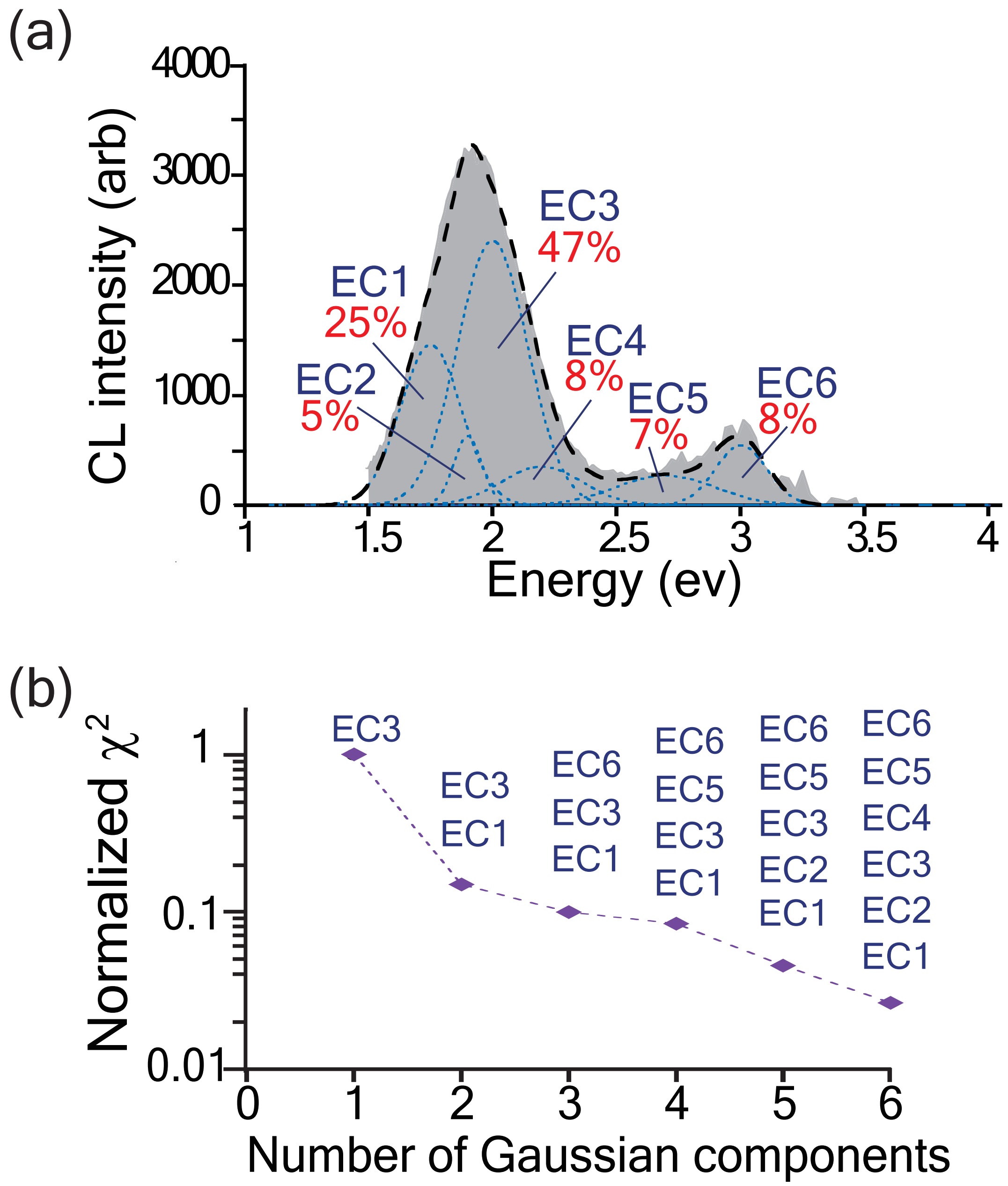

Fig. S1: CL spectrum and separation into six Gaussian components.

(a) An example of typical CL spectrum (the gray area) fitted by six Gaussian components (blue dotted lines) and the sum of those six components (black dotted lines). (b) Fitting residuals when fitted by one to six Gaussian components, assessed by the χ^2 factor normalized by the value fitted by one Gaussian component. To decrease the fitting residuals, we newly adopted emission component (EC) 4 in addition to the five components (EC1, 2, 3, 5, and 6 in this study) used in Ref.6.

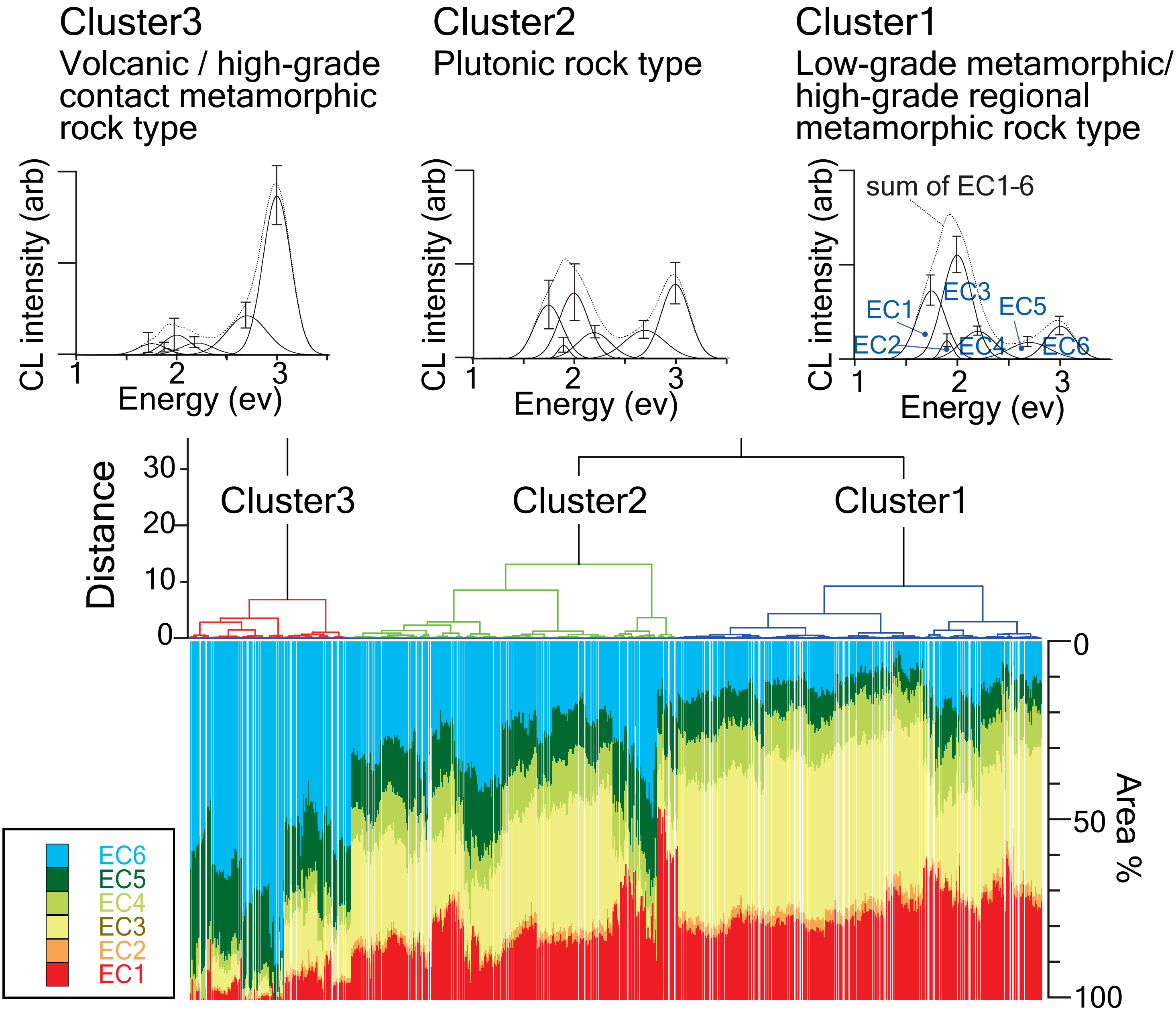

Fig. S2: Cluster analysis result.

Cluster analysis tree diagram obtained using the fractional areas of emission component (EC) 1–6 for quartz grains from the Taklimakan Desert, Gobi Desert, Bering Sea, and Okhotsk Sea (color bars at the bottom). Also shown, at the top of this panel are the relative abundances of EC1–6 for the average of quartz particles in each cluster (solid lines) when the total area of EC1–EC6 (dotted lines) is set to the same value.

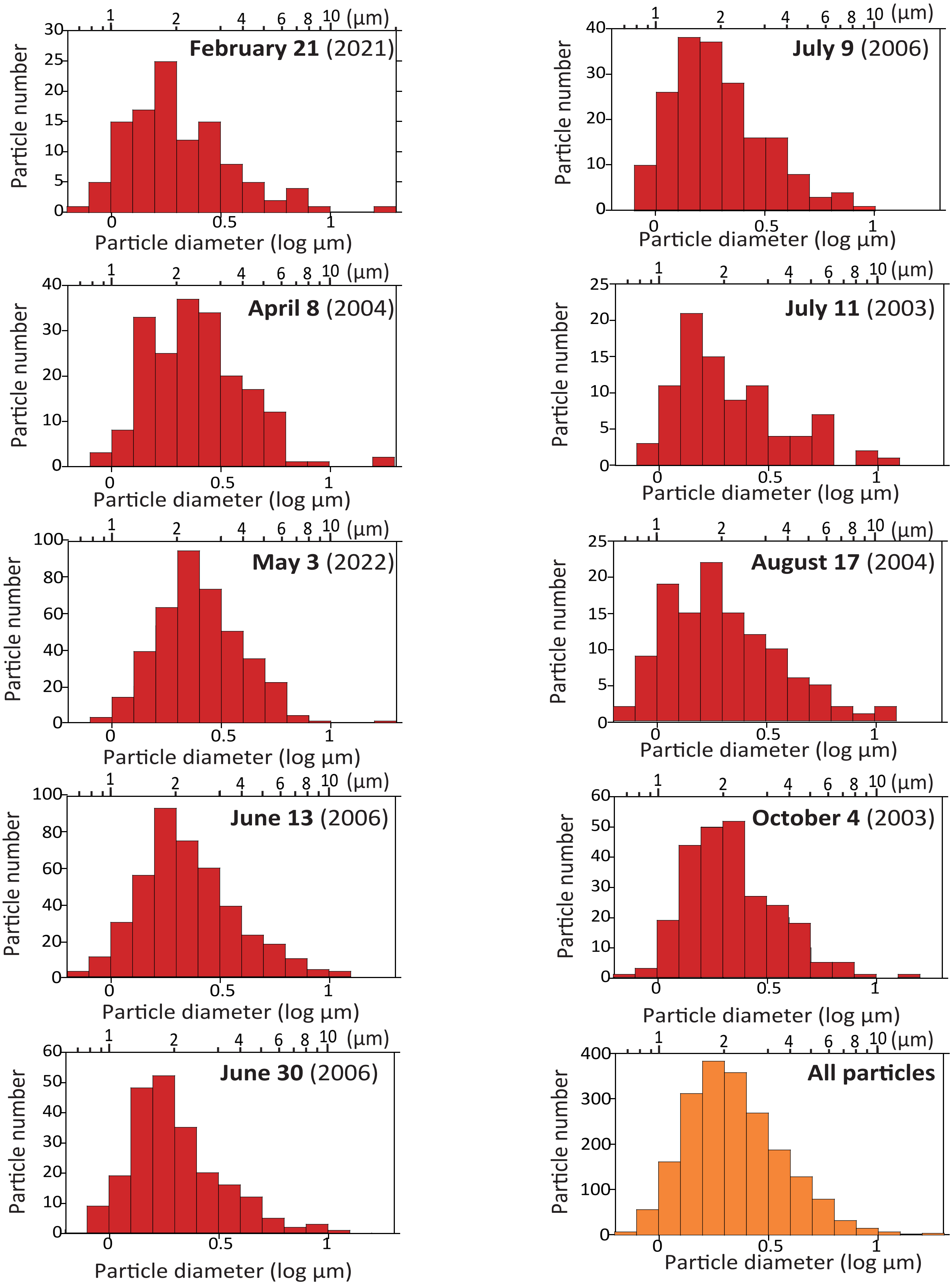

Fig. S3: Histograms showing quartz particle numbers of each particle size class for station K2 samples.

Particle numbers of each particle size (De) class for the station K2 samples derived from 10 and 20 m depths. The samples with larger contribution of volcanic quartz (samples of February 2003 and May 2004) were excluded.

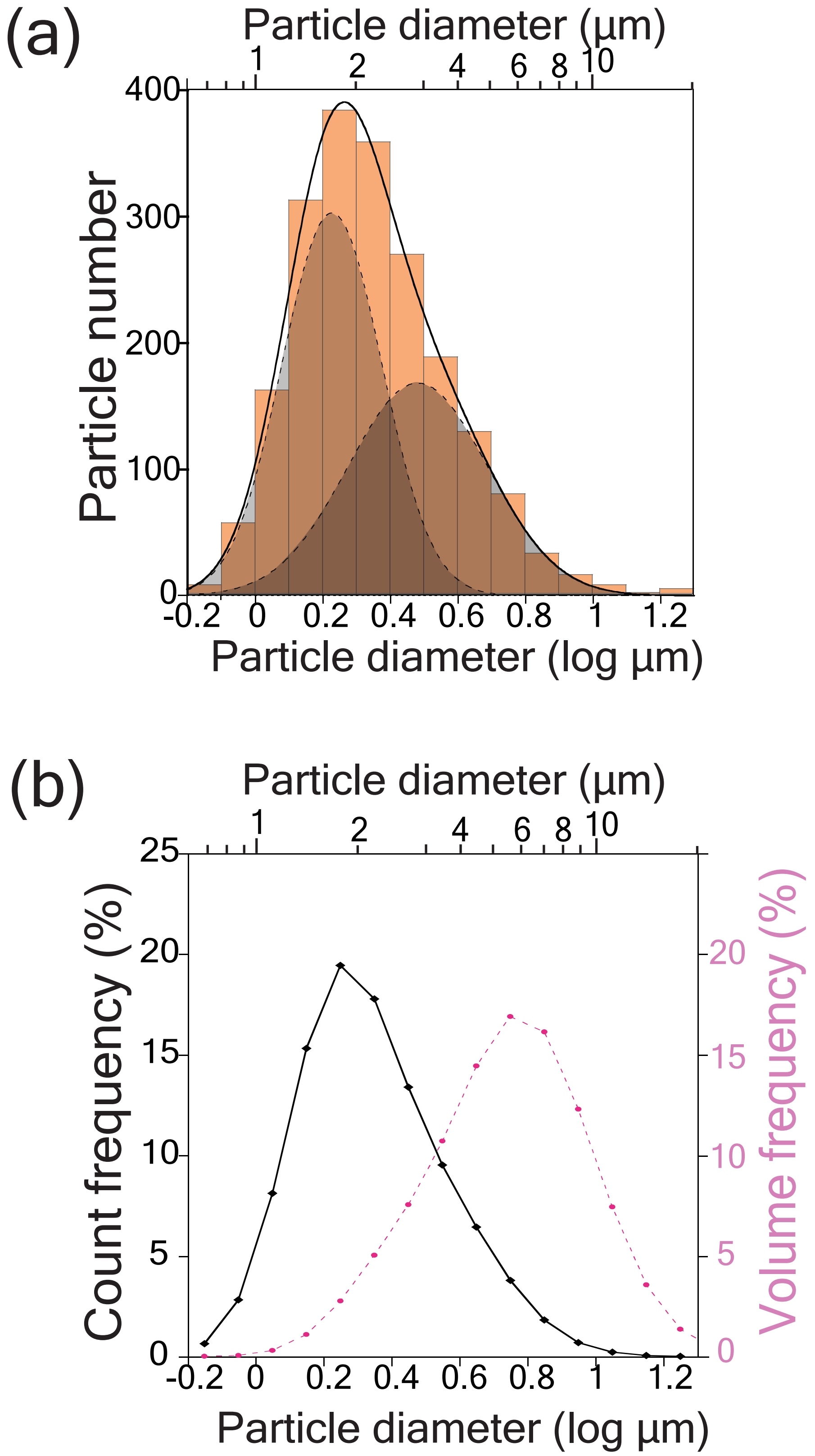

Fig. S4: Calculation of count frequency of quartz particle sizes for dust-dominant K2 samples.

(a) A histogram of particle size (De) classes of dust-dominant samples from St. K2 at 10 and 20 m depths (orange vertical bars) fitted by two Gaussian components (dotted lines) and the sum of those two components (black line). (b) The count frequencies of particle size classes (black line) calculated by the sum of two Gaussian components in (a). The volume frequency of particle size classes (pink dotted line) converted from the count frequency of particle size classes is also shown.

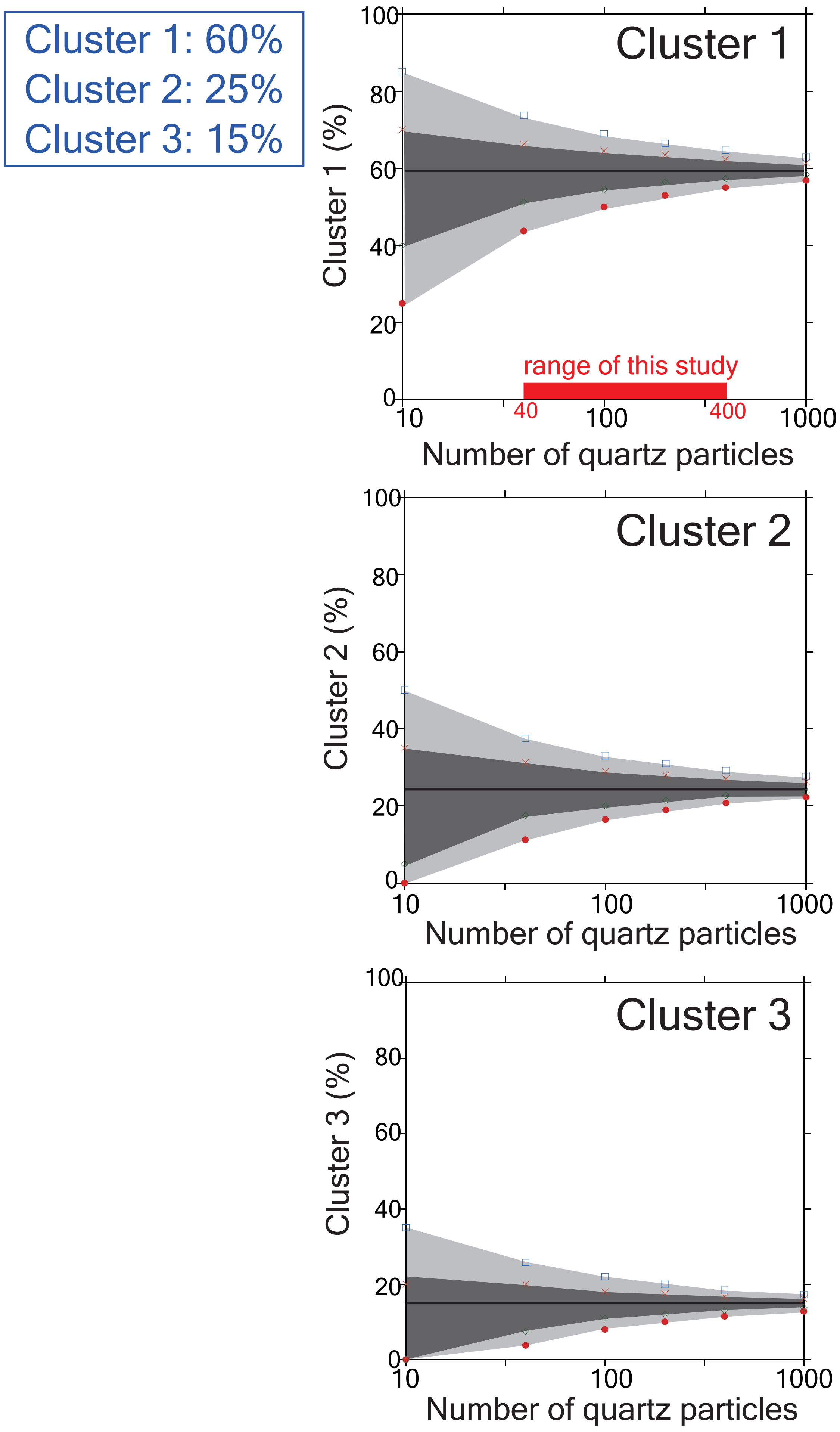

Fig. S5: An example of estimation errors for cluster compositions with number of quartz particles.

The dark and light gray shades show the 68% and 95% confidence intervals, respectively.

Table S1. List of sampling locations and cruises.

Station (cruise)	Location	Latitude (deg North)	Longitude (deg East)	Sample type	Sampling depth	Sampling day	Data DOI
B1 (Kh-07)	Okhotsk Sea	49.50	148.25	in situ filtration	290 m	August 2007	-
G2 (Kh-07)	Okhotsk Sea	55.00	141.00	in situ filtration	90 m	September 2007	-
5 (MR06-04)	Okhotsk Sea	54.24	149.26	Water	0 m	August 2006	10.17596/0001798
6 (MR06-04)	Okhotsk Sea	53.28	150.08	Water	0 m	August 2006	10.17596/0001798
23 (MR06-04)	Bering Sea	60.78	-179.47	Water	0 m	September 2006	10.17596/0001799
32 (MR06-04)	Bering Sea	57.00	-167.50	Water	0 m	September 2006	10.17596/0001799
33 (MR06-04)	Bering Sea	55.77	-166.22	Water	0 m	September 2006	10.17596/0001799
K2 (MR03-K01)	Western subarctic Pacific	47.00	160.00	in situ filtration	10 m, 20 m	25 February 2003	10.17596/0001783
K2 (NT03-07)	Western subarctic Pacific	47.00	160.00	in situ filtration	10 m, 20 m	11 July 2003	10.17596/0000370
K2 (KR03-11)	Western subarctic Pacific	47.00	160.00	in situ filtration	10 m, 20 m	4 October 2003	10.17596/0001006
K2 (MR04-02)	Western subarctic Pacific	47.00	160.00	in situ filtration	10 m, 20 m	8 April 2004	10.17596/0001785
K2 (NT04-05)	Western subarctic Pacific	47.00	160.00	in situ filtration	10 m	27 May 2004	10.17596/0000388
K2 (MR04-04)	Western subarctic Pacific	47.00	160.00	in situ filtration	10 m, 20 m	17 August 2004	10.17596/0001786
K2 (MR06-03, Leg.1)	Western subarctic Pacific	47.00	160.00	in situ filtration	10 m, 20 m	13 June 2006	10.17596/0001796
K2 (MR06-03, Leg.2)	Western subarctic Pacific	47.00	160.00	in situ filtration	10 m, 20 m	30 June 2006	10.17596/0001797
K2 (MR06-03, Leg.2)	Western subarctic Pacific	47.00	160.00	in situ filtration	10 m, 20 m	9 July 2006	10.17596/0001797
K2 (MR21-01)	Western subarctic Pacific	47.00	160.00	Water	10 m,	21 February 2021	10.17596/0002308
K2 (MR22-03)	Western subarctic Pacific	47.00	160.00	Water	10 m, 20 m	3 May 2022	10.17596/0002457
TK1-1	Kunlun Mt. (Taklimakan)	36.42	81.97	Mountain loess	top	-	-
TK1-2	Kunlun Mt. (Taklimakan)	36.42	81.97	Mountain loess	9 m below the top	-	-
TK2	Kunlun Mt. (Taklimakan)	37.02	79.75	Mountain loess	top	-	-
GD1	Gobi	43.77	102.25	Sand dune	surface	-	-
GD2	Gobi	45.93	106.34	Desert	surface	-	-
GD3	Gobi	45.16	101.52	Desert	surface	-	-
GD4'	Gobi	44.346–44.388	105.257–105.276	Desert (dry up pool)	surface	-	-
Nagasaki	Western Japan	32.74	129.87	Dust	-	20–21 March 2010	-

*We combined two samples obtained in the neighbouring area (GD4 and GD5 in Ref. 6).

Table S2. Six emission components (EC) determined for quartz particles in the Asian deserts, Okhotsk Sea, and Bering Sea samples.

	EC1	EC2	EC3	EC4	EC5	EC6
Center position (full width half maximum) (eV)	1.75*	1.9 (0.13)*	2.0 (0.33)*	2.2 (0.38)*	2.7 (0.47)*	2.9–3.2†
Related impurity/imperfection	Substitutional Fe^{3+} impurity centre*	Non-bridging oxygen hole centre*	Non-bridging oxygen hole centre*	Self-trapped exciton*	Self-trapped exciton or Ti impurity*	Al^{3+} impurity centre* or imperfection centre§

* Ref. (7) (with slight modification for centre position/full width half maximum).

† Not fixed because the precise nature of the centre has not yet been solved.

§ Ref. (8) and references therein.

Table S3. Relative abundances of emission components (EC1–EC6) and determined clusters for quartz particles from potential sources.

Station, Location	Quartz particle ID	Fractional area of each Gaussian curve (%)						Cluster
		EC1 (1.75 eV)	EC2 (1.9 eV)	EC3 (2.0 eV)	EC4 (2.2 eV)	EC5 (2.7 eV)	EC6 (2.9–3.2 eV)	
B1, Okhotsk Sea	B1_1	26.7	2.0	34.4	13.8	8.8	14.3	1
B1, Okhotsk Sea	B1_2	21.9	1.9	17.0	11.1	15.8	32.3	2
B1, Okhotsk Sea	B1_3	35.5	2.2	26.2	13.4	10.1	12.7	1
B1, Okhotsk Sea	B1_4	8.9	1.4	17.1	7.2	19.5	45.8	3
B1, Okhotsk Sea	B1_5	29.5	1.6	33.6	11.8	10.8	12.7	1
B1, Okhotsk Sea	B1_6	19.7	2.1	26.1	12.1	13.1	27.0	2
B1, Okhotsk Sea	B1_7	25.7	2.0	31.5	13.3	11.8	15.7	1
B1, Okhotsk Sea	B1_8	30.1	2.2	32.6	11.1	10.2	13.8	1
B1, Okhotsk Sea	B1_9	28.5	1.3	32.9	14.0	9.9	13.4	1
B1, Okhotsk Sea	B1_10	22.9	1.1	21.2	12.8	16.9	25.2	2
B1, Okhotsk Sea	B1_11	30.9	0.2	21.6	12.9	13.9	20.5	1
B1, Okhotsk Sea	B1_12	34.6	0.0	10.1	13.8	17.8	23.7	2
B1, Okhotsk Sea	B1_13	29.0	0.0	4.4	12.6	19.5	34.5	2
B1, Okhotsk Sea	B1_14	36.1	0.0	0.0	21.7	18.6	23.6	2
B1, Okhotsk Sea	B1_15	15.2	1.4	16.4	9.2	22.5	35.2	2
B1, Okhotsk Sea	B1_16	3.5	0.3	0.0	10.1	35.7	50.4	3
B1, Okhotsk Sea	B1_17	29.3	1.9	37.4	12.1	8.5	10.7	1
B1, Okhotsk Sea	B1_18	36.8	1.8	19.2	12.7	11.1	18.4	1
B1, Okhotsk Sea	B1_19	26.9	0.9	17.8	11.7	16.1	26.7	2
B1, Okhotsk Sea	B1_20	28.2	2.1	35.2	13.1	9.0	12.4	1
B1, Okhotsk Sea	B1_21	31.5	0.8	13.5	14.1	15.2	24.9	2
B1, Okhotsk Sea	B1_22	24.9	0.0	2.7	13.2	21.5	37.7	2
B1, Okhotsk Sea	B1_23	35.9	1.1	23.0	13.1	11.1	15.8	1
B1, Okhotsk Sea	B1_24	22.9	2.1	26.1	11.8	14.4	22.7	2
B1, Okhotsk Sea	B1_25	30.5	1.0	28.4	13.6	10.8	15.8	1
B1, Okhotsk Sea	B1_26	33.2	2.1	30.6	13.1	9.5	11.5	1
B1, Okhotsk Sea	B1_27	31.4	1.2	22.4	13.2	13.0	18.8	1
B1, Okhotsk Sea	B1_28	17.1	0.6	8.2	9.9	20.5	43.8	2
B1, Okhotsk Sea	B1_29	16.8	0.3	1.6	10.2	25.7	45.4	2
B1, Okhotsk Sea	B1_30	7.8	1.8	0.0	11.1	32.0	47.2	3
B1, Okhotsk Sea	B1_31	27.8	1.9	30.8	14.2	10.4	14.8	1
B1, Okhotsk Sea	B1_32	30.5	1.4	35.4	13.9	8.1	10.7	1
B1, Okhotsk Sea	B1_33	15.3	2.0	27.2	8.9	13.7	33.1	2
B1, Okhotsk Sea	B1_34	20.1	2.0	31.8	10.7	11.3	24.1	2
B1, Okhotsk Sea	B1_35	25.7	1.9	26.2	13.7	13.9	18.6	1
B1, Okhotsk Sea	B1_36	23.8	2.9	41.1	12.9	8.7	10.7	1
B1, Okhotsk Sea	B1_37	36.9	0.6	29.1	13.0	7.9	12.4	1
B1, Okhotsk Sea	B1_38	26.4	0.7	12.9	12.3	19.5	28.2	2
B1, Okhotsk Sea	B1_39	15.8	1.1	2.5	14.7	30.2	35.8	2
B1, Okhotsk Sea	B1_40	32.9	0.0	0.0	10.3	22.9	34.0	2
B1, Okhotsk Sea	B1_41	29.9	2.3	38.6	12.1	7.6	9.5	1
B1, Okhotsk Sea	B1_42	28.1	2.5	34.8	11.3	9.0	14.3	1
B1, Okhotsk Sea	B1_43	50.8	0.0	12.1	13.1	10.6	13.5	2
B1, Okhotsk Sea	B1_44	26.5	2.2	41.0	12.2	7.3	10.8	1
B1, Okhotsk Sea	B1_45	18.0	1.3	28.2	10.4	14.9	27.2	2
B1, Okhotsk Sea	B1_46	0.9	0.4	2.1	2.4	21.3	72.9	3
B1, Okhotsk Sea	B1_47	25.8	0.0	4.5	11.0	19.9	38.8	2
B1, Okhotsk Sea	B1_48	26.6	2.0	30.9	12.9	10.2	17.4	1
B1, Okhotsk Sea	B1_49	26.4	2.3	25.6	13.0	13.4	19.3	1
B1, Okhotsk Sea	B1_50	25.6	1.3	25.1	11.7	14.6	21.7	2
B1, Okhotsk Sea	B1_51	22.4	2.4	33.8	12.0	12.1	17.4	1
B1, Okhotsk Sea	B1_52	17.3	0.3	8.4	10.4	24.2	39.3	2
B1, Okhotsk Sea	B1_53	23.0	2.3	39.5	12.0	9.3	13.8	1
B1, Okhotsk Sea	B1_54	32.5	3.0	33.9	11.6	9.0	10.1	1
B1, Okhotsk Sea	B1_55	39.4	0.0	27.9	12.7	8.2	11.8	1
B1, Okhotsk Sea	B1_56	22.1	0.8	23.7	13.0	13.3	27.2	2
B1, Okhotsk Sea	B1_57	39.8	0.3	25.3	12.9	9.8	11.9	1
B1, Okhotsk Sea	B1_58	31.2	0.0	9.1	12.3	19.4	28.0	2
B1, Okhotsk Sea	B1_59	26.5	1.2	22.1	10.8	16.2	23.2	2
B1, Okhotsk Sea	B1_60	28.7	2.1	37.2	13.5	7.9	10.6	1
G2, Okhotsk Sea	G2_1	37.6	2.0	22.3	16.2	9.4	12.6	1
G2, Okhotsk Sea	G2_2	53.9	0.0	10.2	10.4	12.1	13.3	2
G2, Okhotsk Sea	G2_3	29.5	0.0	3.7	17.4	20.5	28.9	2
G2, Okhotsk Sea	G2_4	46.3	0.0	15.0	12.9	12.5	13.4	2
G2, Okhotsk Sea	G2_5	30.4	0.6	21.0	15.6	13.9	18.5	1
G2, Okhotsk Sea	G2_6	20.6	0.0	13.4	11.4	19.3	35.2	2
G2, Okhotsk Sea	G2_7	39.2	0.3	11.9	16.5	14.6	17.6	2
G2, Okhotsk Sea	G2_8	17.6	0.6	15.3	9.3	17.5	39.7	2
G2, Okhotsk Sea	G2_9	22.1	1.1	20.8	13.1	17.2	25.7	2
G2, Okhotsk Sea	G2_10	2.7	0.4	5.1	4.0	22.2	65.6	3
G2, Okhotsk Sea	G2_11	32.0	0.0	6.9	11.2	19.5	30.4	2
G2, Okhotsk Sea	G2_12	4.3	0.3	5.4	4.1	22.8	63.1	3
G2, Okhotsk Sea	G2_13	41.8	0.0	14.8	14.3	14.2	14.9	2
G2, Okhotsk Sea	G2_14	32.9	0.0	8.3	12.4	18.2	28.2	2
G2, Okhotsk Sea	G2_15	40.8	0.1	14.4	14.3	13.8	16.6	2
G2, Okhotsk Sea	G2_16	33.5	1.4	30.8	13.0	10.1	11.2	1
G2, Okhotsk Sea	G2_17	33.2	1.0	16.9	16.1	14.7	18.0	1
G2, Okhotsk Sea	G2_18	37.9	0.0	11.6	11.5	15.5	23.5	2
G2, Okhotsk Sea	G2_19	15.1	1.3	19.9	9.4	17.0	37.2	2
G2, Okhotsk Sea	G2_20	18.2	1.6	23.5	13.9	16.2	26.5	2
G2, Okhotsk Sea	G2_21	31.3	1.2	23.7	13.2	14.4	16.1	1
G2, Okhotsk Sea	G2_22	34.0	1.4	20.3	15.5	13.9	14.9	1

G2, Okhotsk Sea	G2_23	41.6	0.6	12.2	14.3	13.0	18.4	2
G2, Okhotsk Sea	G2_24	34.0	0.0	17.6	13.0	15.7	19.6	1
G2, Okhotsk Sea	G2_25	24.7	1.6	23.8	12.1	15.2	22.6	2
G2, Okhotsk Sea	G2_26	50.9	0.0	1.7	17.7	14.7	15.0	2
G2, Okhotsk Sea	G2_27	31.5	1.8	30.6	13.5	11.2	11.5	1
G2, Okhotsk Sea	G2_28	25.5	2.1	34.1	14.0	11.4	13.0	1
G2, Okhotsk Sea	G2_29	28.1	0.0	1.4	11.7	27.2	31.6	2
G2, Okhotsk Sea	G2_30	27.0	1.3	22.6	13.1	13.4	22.6	2
G2, Okhotsk Sea	G2_31	27.6	1.1	26.1	13.6	13.5	18.2	1
G2, Okhotsk Sea	G2_32	38.8	0.5	19.2	11.7	12.9	16.9	1
G2, Okhotsk Sea	G2_33	27.7	1.7	24.6	11.9	12.8	21.3	2
G2, Okhotsk Sea	G2_34	23.6	1.4	12.2	12.9	17.9	32.0	2
G2, Okhotsk Sea	G2_35	26.9	1.2	13.7	13.9	17.3	27.1	2
G2, Okhotsk Sea	G2_36	29.0	0.0	1.9	10.6	20.0	38.4	2
G2, Okhotsk Sea	G2_37	0.1	0.0	0.0	2.5	28.1	69.3	3
G2, Okhotsk Sea	G2_38	30.3	0.1	2.5	6.5	22.5	38.0	2
G2, Okhotsk Sea	G2_39	39.6	0.1	7.8	16.1	15.6	20.8	2
G2, Okhotsk Sea	G2_40	24.4	0.2	8.7	9.7	21.0	35.9	2
G2, Okhotsk Sea	G2_41	34.4	1.4	27.9	15.0	10.7	10.6	1
G2, Okhotsk Sea	G2_42	20.0	0.7	4.4	10.1	21.0	43.8	2
G2, Okhotsk Sea	G2_43	5.1	0.4	7.2	4.9	22.0	60.5	3
G2, Okhotsk Sea	G2_44	12.3	0.5	8.6	7.8	19.5	51.3	3
G2, Okhotsk Sea	G2_45	11.6	0.1	0.0	5.1	23.0	60.2	3
G2, Okhotsk Sea	G2_46	53.2	0.0	12.7	8.2	10.6	15.2	2
G2, Okhotsk Sea	G2_47	5.4	0.1	4.1	4.2	23.0	63.3	3
G2, Okhotsk Sea	G2_48	46.8	0.0	7.4	12.9	19.4	13.6	2
G2, Okhotsk Sea	G2_49	33.1	1.0	21.4	14.6	13.7	16.3	1
G2, Okhotsk Sea	G2_50	38.1	0.0	11.3	15.2	18.1	17.2	2
G2, Okhotsk Sea	G2_51	42.0	0.0	5.2	15.5	16.0	21.3	2
G2, Okhotsk Sea	G2_52	39.9	0.5	9.5	14.1	15.1	20.8	2
G2, Okhotsk Sea	G2_53	23.4	0.2	0.7	9.1	22.0	44.6	2
G2, Okhotsk Sea	G2_54	34.5	0.0	0.0	12.3	21.6	31.5	2
G2, Okhotsk Sea	G2_55	5.9	0.3	4.8	4.8	22.9	61.3	3
G2, Okhotsk Sea	G2_56	22.7	2.1	36.0	11.8	11.5	15.9	1
G2, Okhotsk Sea	G2_57	51.0	0.0	18.6	10.2	9.8	10.4	2
G2, Okhotsk Sea	G2_58	42.2	0.0	10.2	12.3	15.4	19.9	2
G2, Okhotsk Sea	G2_59	13.0	1.1	18.4	9.2	18.7	39.6	2
G2, Okhotsk Sea	G2_60	33.0	1.1	22.7	15.1	12.2	16.0	1
5, Okhotsk Sea	5_1	12.6	1.9	26.2	12.4	16.9	30.0	2
5, Okhotsk Sea	5_2	19.7	2.9	45.1	13.7	8.2	10.4	1
5, Okhotsk Sea	5_3	16.6	2.3	35.4	11.5	11.1	23.1	2
5, Okhotsk Sea	5_4	16.6	2.5	37.4	13.8	12.1	17.6	2
5, Okhotsk Sea	5_5	0.0	0.0	0.0	7.9	37.0	55.0	3
5, Okhotsk Sea	5_6	13.8	2.4	30.9	12.8	16.0	24.1	2
5, Okhotsk Sea	5_7	5.5	1.2	9.8	7.7	22.9	52.9	3
5, Okhotsk Sea	5_8	18.4	2.6	41.9	14.2	10.1	12.7	1
5, Okhotsk Sea	5_9	26.5	1.0	28.2	17.1	10.6	16.6	1
5, Okhotsk Sea	5_10	11.9	1.8	18.9	11.7	19.6	36.1	2
5, Okhotsk Sea	5_11	17.9	2.6	37.5	15.0	12.3	14.8	1
5, Okhotsk Sea	5_12	18.8	2.6	40.4	14.7	10.3	13.2	1
5, Okhotsk Sea	5_13	11.8	2.6	27.1	10.4	14.3	33.9	2
5, Okhotsk Sea	5_14	0.0	0.6	0.0	2.8	19.9	76.6	3
5, Okhotsk Sea	5_15	19.8	0.1	24.6	10.1	9.7	35.8	2
5, Okhotsk Sea	5_16	9.8	1.0	10.2	8.9	19.2	50.9	3
5, Okhotsk Sea	5_17	17.2	2.4	35.8	14.8	12.3	17.4	2
5, Okhotsk Sea	5_18	3.7	1.6	11.5	2.3	4.3	76.7	3
5, Okhotsk Sea	5_19	17.6	2.6	41.2	14.3	10.8	13.5	1
5, Okhotsk Sea	5_20	6.6	1.5	17.4	5.0	9.7	59.8	3
5, Okhotsk Sea	5_21	18.2	3.0	41.0	13.4	11.3	13.0	1
5, Okhotsk Sea	5_22	0.7	0.6	3.3	3.1	22.6	69.7	3
5, Okhotsk Sea	5_23	15.6	2.7	36.5	13.8	12.1	19.4	2
5, Okhotsk Sea	5_24	2.2	0.9	1.2	10.0	28.7	57.0	3
5, Okhotsk Sea	5_25	13.0	2.4	30.7	11.9	13.2	28.8	2
5, Okhotsk Sea	5_26	6.4	2.2	15.6	10.2	21.9	43.7	3
5, Okhotsk Sea	5_27	1.7	1.0	6.0	4.3	22.7	64.3	3
5, Okhotsk Sea	5_28	14.3	2.5	34.2	10.9	11.1	26.9	2
5, Okhotsk Sea	5_29	15.2	2.2	25.5	10.2	12.5	34.5	2
5, Okhotsk Sea	5_30	16.6	2.3	31.5	14.1	16.3	19.1	2
5, Okhotsk Sea	5_31	15.8	2.2	31.9	12.2	14.3	23.6	2
5, Okhotsk Sea	5_32	14.0	2.5	25.4	9.9	13.9	34.4	2
5, Okhotsk Sea	5_33	15.8	2.4	38.3	14.7	12.3	16.5	2
5, Okhotsk Sea	5_34	17.3	2.9	38.3	15.6	12.3	13.6	1
5, Okhotsk Sea	5_35	25.4	3.6	43.6	12.6	6.8	8.0	1
5, Okhotsk Sea	5_36	0.9	0.8	3.8	2.6	5.4	86.4	3
5, Okhotsk Sea	5_37	0.6	0.6	3.2	2.9	21.9	70.8	3
5, Okhotsk Sea	5_38	9.7	1.4	19.4	9.3	18.3	42.0	2
5, Okhotsk Sea	5_39	18.5	2.7	38.9	13.4	11.3	15.2	1
5, Okhotsk Sea	5_40	14.7	2.6	32.4	13.0	15.5	21.8	2
5, Okhotsk Sea	5_41	28.3	1.1	22.8	9.6	10.5	27.8	2
5, Okhotsk Sea	5_42	15.0	2.9	25.4	11.8	14.0	30.9	2
5, Okhotsk Sea	5_43	19.0	2.8	39.8	16.0	9.4	13.0	1
5, Okhotsk Sea	5_44	15.3	2.6	35.1	13.5	14.8	18.8	2
5, Okhotsk Sea	5_45	16.4	2.4	35.7	14.3	13.6	17.7	2
5, Okhotsk Sea	5_46	1.8	0.9	6.5	4.4	22.7	63.8	3
5, Okhotsk Sea	5_47	18.2	2.6	28.5	14.4	13.7	22.6	2
6, Okhotsk Sea	6_1	2.9	0.9	8.7	4.9	20.3	62.5	3
6, Okhotsk Sea	6_2	2.5	0.8	7.1	4.2	20.9	64.5	3
6, Okhotsk Sea	6_3	17.0	1.8	27.7	14.0	17.1	22.4	2
6, Okhotsk Sea	6_4	1.1	0.7	4.7	3.4	21.8	68.2	3

6, Okhotsk Sea	6_5	0.0	0.2	0.0	9.2	38.3	52.3	3
6, Okhotsk Sea	6_6	13.5	2.5	30.3	11.7	15.2	26.8	2
6, Okhotsk Sea	6_7	17.5	2.8	39.8	13.3	11.2	15.4	1
6, Okhotsk Sea	6_8	17.6	2.7	38.3	12.6	11.4	17.3	2
6, Okhotsk Sea	6_9	17.0	2.3	31.2	12.9	14.7	21.9	2
6, Okhotsk Sea	6_10	16.9	2.6	38.2	13.6	11.9	16.9	2
6, Okhotsk Sea	6_11	14.6	2.4	34.4	13.9	14.7	20.0	2
6, Okhotsk Sea	6_12	38.3	0.0	22.5	16.0	8.3	14.9	1
6, Okhotsk Sea	6_13	17.3	1.1	25.6	12.3	14.5	29.3	2
6, Okhotsk Sea	6_14	20.3	1.7	30.8	14.0	13.3	19.8	2
6, Okhotsk Sea	6_15	18.8	2.9	38.4	14.1	12.1	13.7	1
6, Okhotsk Sea	6_16	14.5	2.6	30.5	11.3	14.7	26.4	2
6, Okhotsk Sea	6_17	11.0	1.7	12.6	15.0	25.0	34.7	2
6, Okhotsk Sea	6_18	7.1	1.4	16.3	9.0	20.6	45.7	3
6, Okhotsk Sea	6_19	10.9	0.7	0.0	22.9	36.1	29.5	2
6, Okhotsk Sea	6_20	1.1	0.7	4.8	3.5	21.5	68.3	3
6, Okhotsk Sea	6_21	9.2	2.1	23.1	9.8	17.4	38.5	2
6, Okhotsk Sea	6_22	13.6	2.2	26.1	14.3	19.6	24.2	2
6, Okhotsk Sea	6_23	14.9	2.1	20.9	13.5	20.1	28.5	2
6, Okhotsk Sea	6_24	1.4	0.7	5.0	4.0	22.3	66.4	3
6, Okhotsk Sea	6_25	8.7	1.8	19.3	11.6	24.9	33.9	2
6, Okhotsk Sea	6_26	11.0	0.7	12.4	7.4	17.0	51.5	3
6, Okhotsk Sea	6_27	16.5	2.4	35.7	12.5	12.8	20.2	2
6, Okhotsk Sea	6_28	14.6	2.7	32.8	12.4	12.9	24.6	2
6, Okhotsk Sea	6_29	20.0	2.6	43.3	14.0	9.0	11.0	1
6, Okhotsk Sea	6_30	14.7	2.8	34.2	13.8	12.5	22.1	2
6, Okhotsk Sea	6_31	15.9	1.6	27.1	12.2	15.8	27.4	2
6, Okhotsk Sea	6_32	20.2	2.7	41.7	14.5	10.1	10.9	1
6, Okhotsk Sea	6_33	8.3	1.4	19.1	8.0	18.5	44.7	3
6, Okhotsk Sea	6_34	19.7	2.7	41.7	13.7	9.8	12.3	1
6, Okhotsk Sea	6_35	5.2	1.6	14.2	5.5	16.5	56.9	3
6, Okhotsk Sea	6_36	17.3	2.5	40.7	13.5	10.7	15.2	1
6, Okhotsk Sea	6_37	5.1	1.5	14.9	6.9	20.7	50.9	3
6, Okhotsk Sea	6_38	7.9	1.7	19.8	7.0	17.0	46.6	3
6, Okhotsk Sea	6_39	14.2	2.2	30.6	11.2	14.3	27.4	2
6, Okhotsk Sea	6_40	0.7	0.6	2.7	3.6	23.1	69.3	3
6, Okhotsk Sea	6_41	11.6	2.5	23.8	14.7	19.5	28.0	2
6, Okhotsk Sea	6_42	9.0	1.7	20.7	8.8	17.9	41.9	2
6, Okhotsk Sea	6_43	16.9	2.5	36.5	14.6	13.5	16.0	2
6, Okhotsk Sea	6_44	10.5	1.6	18.9	11.8	16.7	40.5	2
6, Okhotsk Sea	6_45	17.0	0.0	0.0	5.1	33.9	44.0	3
6, Okhotsk Sea	6_46	18.3	2.6	41.1	13.5	9.1	15.4	1
6, Okhotsk Sea	6_47	13.7	1.8	24.1	12.7	17.9	29.7	2
6, Okhotsk Sea	6_48	16.2	2.5	38.8	13.4	11.5	17.6	2
6, Okhotsk Sea	6_49	7.7	1.6	19.8	6.9	16.4	47.7	3
6, Okhotsk Sea	6_50	0.0	0.0	0.0	0.0	10.0	90.0	3
23, Bering Sea	23_1	1.7	0.0	0.0	4.3	30.5	63.4	3
23, Bering Sea	23_2	2.2	1.1	8.1	3.9	22.2	62.6	3
23, Bering Sea	23_3	12.3	2.1	30.1	9.3	15.1	31.1	2
23, Bering Sea	23_4	4.8	1.6	14.6	6.4	22.2	50.4	3
23, Bering Sea	23_5	0.3	0.0	0.0	6.6	34.6	58.6	3
23, Bering Sea	23_6	6.2	1.1	13.6	5.7	20.9	52.5	3
23, Bering Sea	23_7	0.0	0.7	1.5	1.7	14.8	81.4	3
23, Bering Sea	23_8	20.7	2.9	39.5	12.7	9.8	14.4	1
23, Bering Sea	23_9	3.9	1.2	10.8	4.4	21.4	58.3	3
23, Bering Sea	23_10	14.2	2.3	28.5	10.8	16.8	27.3	2
23, Bering Sea	23_11	21.2	3.0	44.4	12.0	8.7	10.7	1
23, Bering Sea	23_12	1.0	0.0	0.0	2.5	11.2	85.4	3
23, Bering Sea	23_13	22.5	2.6	40.5	10.9	9.4	14.1	1
23, Bering Sea	23_14	20.9	3.5	46.9	13.0	6.6	9.0	1
23, Bering Sea	23_15	4.7	1.7	10.2	8.5	23.9	51.0	3
23, Bering Sea	23_16	19.2	3.0	42.6	12.3	9.8	13.1	1
23, Bering Sea	23_17	23.3	2.5	41.5	13.0	8.6	11.1	1
23, Bering Sea	23_18	13.2	2.4	32.8	10.8	17.7	23.0	2
23, Bering Sea	23_19	12.3	3.0	28.8	8.0	9.7	38.2	2
23, Bering Sea	23_20	20.7	2.3	36.9	12.8	12.2	15.1	1
23, Bering Sea	23_21	17.4	2.4	30.9	15.8	13.7	19.6	2
23, Bering Sea	23_22	9.4	1.9	11.8	8.4	20.2	48.2	3
23, Bering Sea	23_23	23.4	2.7	35.9	12.9	10.3	14.8	1
23, Bering Sea	23_24	10.9	1.5	18.3	10.8	21.7	36.8	2
23, Bering Sea	23_25	37.0	0.0	28.3	12.5	9.8	12.3	1
23, Bering Sea	23_26	0.0	0.0	0.0	3.0	21.6	75.5	3
23, Bering Sea	23_27	35.0	0.4	18.7	9.6	13.8	22.5	1
23, Bering Sea	23_28	17.8	2.2	33.7	12.9	14.9	18.5	2
23, Bering Sea	23_29	8.1	1.8	21.8	7.1	19.8	41.5	2
23, Bering Sea	23_30	19.6	2.3	37.5	11.7	12.7	16.2	1
23, Bering Sea	23_31	4.6	0.5	6.7	4.7	22.9	60.6	3
23, Bering Sea	23_32	16.9	2.8	40.1	12.1	11.4	16.7	1
23, Bering Sea	23_33	19.4	2.7	36.3	13.5	13.6	14.5	1
23, Bering Sea	23_34	16.6	2.7	36.9	11.7	10.8	21.3	2
23, Bering Sea	23_35	0.0	0.0	0.0	7.3	34.5	58.2	3
23, Bering Sea	23_36	21.7	2.4	39.2	12.2	11.4	13.2	1
23, Bering Sea	23_37	18.3	2.2	34.9	11.9	13.0	19.7	2
23, Bering Sea	23_38	13.2	2.7	20.8	12.6	20.3	30.4	2
23, Bering Sea	23_39	23.1	3.0	45.2	13.7	6.1	8.8	1
23, Bering Sea	23_40	17.7	2.4	36.3	12.8	12.0	18.8	2
23, Bering Sea	23_41	1.9	1.0	6.3	3.6	23.1	64.2	3
23, Bering Sea	23_42	24.7	1.3	16.3	12.5	16.6	28.6	2
23, Bering Sea	23_43	21.2	2.9	43.8	14.0	7.9	10.2	1

23, Bering Sea	23_44	0.0	0.0	0.0	6.0	33.8	60.2	3
23, Bering Sea	23_45	18.3	2.9	39.7	13.8	11.4	13.9	1
23, Bering Sea	23_46	12.4	2.5	26.7	10.6	16.3	31.6	2
23, Bering Sea	23_47	16.7	3.3	28.4	11.5	13.7	26.4	2
23, Bering Sea	23_48	17.8	2.9	37.5	11.7	11.3	18.9	2
23, Bering Sea	23_49	2.5	1.1	2.7	9.5	27.0	57.1	3
32, Bering Sea	32_1	16.7	1.3	15.0	14.5	19.1	33.4	2
32, Bering Sea	32_2	23.1	0.6	8.3	13.5	19.3	35.2	2
32, Bering Sea	32_3	21.5	1.0	22.2	11.9	17.1	26.3	2
32, Bering Sea	32_4	23.5	2.1	34.1	12.1	12.3	15.8	1
32, Bering Sea	32_5	19.5	2.6	36.8	13.9	11.5	15.8	1
32, Bering Sea	32_6	22.4	1.0	15.1	14.0	19.4	28.1	2
32, Bering Sea	32_7	1.6	0.0	0.0	2.0	23.8	72.5	3
32, Bering Sea	32_8	20.9	1.1	18.0	15.4	17.5	27.3	2
32, Bering Sea	32_9	12.1	1.4	20.7	9.5	17.0	39.2	2
32, Bering Sea	32_10	0.1	0.0	0.0	7.1	36.6	56.2	3
32, Bering Sea	32_11	4.1	1.1	10.6	4.6	20.0	59.6	3
32, Bering Sea	32_12	8.4	0.0	0.0	8.7	36.8	46.1	3
32, Bering Sea	32_13	9.0	1.1	14.7	7.3	18.5	49.4	3
32, Bering Sea	32_14	0.4	0.0	0.0	2.8	23.9	72.8	3
32, Bering Sea	32_15	17.8	0.9	17.4	10.0	17.7	36.2	2
32, Bering Sea	32_16	30.3	1.1	26.8	13.7	12.3	15.8	1
32, Bering Sea	32_17	36.4	0.0	0.0	11.2	22.3	30.1	2
32, Bering Sea	32_18	28.5	1.2	30.3	15.5	11.2	13.4	1
32, Bering Sea	32_19	16.0	1.3	22.5	11.5	17.0	31.6	2
32, Bering Sea	32_20	23.0	2.3	36.7	14.5	10.2	13.3	1
32, Bering Sea	32_21	25.9	1.9	29.9	14.7	12.0	15.6	1
32, Bering Sea	32_22	28.1	1.9	33.6	13.8	10.8	11.8	1
32, Bering Sea	32_23	20.1	1.7	29.2	11.3	13.4	24.3	2
32, Bering Sea	32_24	30.2	1.5	33.7	13.0	10.4	11.3	1
32, Bering Sea	32_25	18.8	1.1	25.3	12.3	16.4	26.1	2
32, Bering Sea	32_26	23.9	2.3	38.2	13.9	10.5	11.2	1
32, Bering Sea	32_27	25.4	0.4	21.5	17.3	16.3	19.2	2
32, Bering Sea	32_28	16.0	2.3	27.0	12.7	15.8	26.1	2
32, Bering Sea	32_29	17.4	1.8	25.9	11.5	13.9	29.5	2
32, Bering Sea	32_30	24.3	2.1	19.7	13.4	15.7	24.8	2
32, Bering Sea	32_31	17.6	1.8	29.6	11.9	12.7	26.4	2
32, Bering Sea	32_32	20.9	1.8	33.1	11.7	10.4	22.0	2
32, Bering Sea	32_33	35.7	0.0	0.2	18.4	17.3	28.4	2
32, Bering Sea	32_34	24.2	1.3	28.7	14.6	13.0	18.1	1
32, Bering Sea	32_35	27.6	1.0	29.7	14.0	11.9	15.8	1
32, Bering Sea	32_36	27.0	1.2	29.5	16.8	11.4	14.1	1
32, Bering Sea	32_37	6.4	0.5	6.0	5.9	23.4	57.9	3
32, Bering Sea	32_38	2.9	0.0	0.0	5.1	23.2	68.8	3
32, Bering Sea	32_39	23.7	1.6	29.3	14.4	12.9	18.1	1
32, Bering Sea	32_40	26.4	1.0	30.1	14.3	13.0	15.1	1
32, Bering Sea	32_41	22.5	1.3	31.6	14.1	13.5	17.0	1
32, Bering Sea	32_42	15.4	2.2	23.7	8.6	14.3	35.7	2
32, Bering Sea	32_43	19.5	3.1	39.7	14.3	9.7	13.7	1
32, Bering Sea	32_44	21.2	2.3	33.7	12.9	11.7	18.2	1
32, Bering Sea	32_45	13.7	0.3	7.3	7.5	21.6	49.6	3
32, Bering Sea	32_46	25.7	0.9	29.2	14.6	12.1	17.5	1
32, Bering Sea	32_47	2.6	0.0	5.0	3.3	23.1	66.1	3
32, Bering Sea	32_48	23.0	1.2	19.9	14.7	16.9	24.3	2
32, Bering Sea	32_49	18.7	0.4	20.7	11.7	16.8	31.6	2
32, Bering Sea	32_50	22.0	1.8	30.2	13.8	14.3	17.9	1
32, Bering Sea	32_51	22.3	1.9	23.2	13.4	17.1	22.1	2
32, Bering Sea	32_52	6.0	0.0	6.9	4.6	24.1	58.5	3
32, Bering Sea	32_53	32.9	0.0	10.0	12.1	17.1	27.9	2
32, Bering Sea	32_54	24.9	1.4	33.0	13.6	12.2	15.0	1
33, Bering Sea	33_1	0.0	0.0	0.0	3.1	18.8	78.0	3
33, Bering Sea	33_2	0.0	0.0	0.0	0.0	9.5	90.5	3
33, Bering Sea	33_3	17.3	2.5	39.3	12.8	11.5	16.6	1
33, Bering Sea	33_4	18.0	2.9	39.3	13.1	12.7	14.1	1
33, Bering Sea	33_5	0.0	0.0	0.0	2.8	24.3	72.8	3
33, Bering Sea	33_6	5.7	0.8	10.1	6.1	21.8	55.4	3
33, Bering Sea	33_7	18.1	2.8	40.4	12.7	11.1	14.9	1
33, Bering Sea	33_8	1.8	0.8	5.7	4.0	21.7	65.9	3
33, Bering Sea	33_9	18.9	1.6	23.4	15.6	17.8	22.7	2
33, Bering Sea	33_10	17.3	2.6	37.1	13.9	13.5	15.6	2
33, Bering Sea	33_11	16.2	2.6	35.8	13.0	14.2	18.3	2
33, Bering Sea	33_12	16.2	2.6	34.4	13.5	15.6	17.7	2
33, Bering Sea	33_13	2.5	2.3	4.9	7.4	26.2	56.6	3
33, Bering Sea	33_14	3.6	0.7	5.4	4.1	21.5	64.7	3
33, Bering Sea	33_15	16.3	2.3	34.9	12.9	13.2	20.4	2
33, Bering Sea	33_16	3.0	0.6	4.8	4.5	21.8	65.3	3
33, Bering Sea	33_17	21.2	3.2	43.9	14.1	9.2	8.4	1
33, Bering Sea	33_18	18.9	2.9	38.6	14.4	11.1	14.2	1
33, Bering Sea	33_19	21.9	3.1	36.0	12.6	11.4	15.1	1
33, Bering Sea	33_20	14.5	2.9	29.2	12.2	16.6	24.6	2
33, Bering Sea	33_21	21.8	1.8	22.1	13.7	13.2	27.4	2
33, Bering Sea	33_22	15.6	2.3	25.7	11.3	16.1	29.0	2
33, Bering Sea	33_23	24.4	3.4	43.6	12.5	7.2	8.9	1
33, Bering Sea	33_24	4.8	1.3	14.9	5.2	18.5	55.3	3
33, Bering Sea	33_25	24.7	2.2	37.4	12.8	9.7	13.2	1
33, Bering Sea	33_26	12.6	2.6	20.4	11.7	21.7	31.0	2
33, Bering Sea	33_27	2.8	1.2	8.5	4.0	22.5	61.0	3
33, Bering Sea	33_28	22.1	2.7	44.1	12.3	7.3	11.4	1
33, Bering Sea	33_29	24.1	3.2	42.5	12.9	7.6	9.8	1

33, Bering Sea	33_30	18.1	2.5	39.0	12.5	12.3	15.7	1
33, Bering Sea	33_31	14.1	2.2	28.7	8.7	14.5	31.7	2
33, Bering Sea	33_32	9.0	1.9	21.5	8.2	18.7	40.7	2
33, Bering Sea	33_33	17.1	2.1	21.9	12.8	21.0	25.2	2
33, Bering Sea	33_34	2.6	1.0	8.3	3.8	22.1	62.1	3
33, Bering Sea	33_35	22.6	3.0	43.7	12.0	8.1	10.7	1
33, Bering Sea	33_36	0.0	0.3	1.0	2.1	23.3	73.3	3
33, Bering Sea	33_37	0.0	0.0	0.0	0.0	10.6	89.4	3
33, Bering Sea	33_38	11.7	2.0	27.9	9.9	14.6	33.9	2
33, Bering Sea	33_39	21.3	2.9	39.3	14.9	8.8	12.7	1
33, Bering Sea	33_40	5.2	3.1	12.2	10.5	26.5	42.5	3
33, Bering Sea	33_41	21.6	3.0	43.6	13.5	7.5	10.7	1
33, Bering Sea	33_42	3.3	1.1	4.3	8.9	28.2	54.3	3
33, Bering Sea	33_43	21.6	2.8	43.3	13.8	8.1	10.4	1
33, Bering Sea	33_44	14.5	2.6	34.7	10.9	13.5	23.8	2
TK1-1, Kunlun Mt. (Taklimakan Desert)	TK1-1_1	30.5	3.7	41.1	10.3	7.3	7.1	1
TK1-1, Kunlun Mt. (Taklimakan Desert)	TK1-1_2	34.2	5.4	31.9	17.5	5.2	5.8	1
TK1-1, Kunlun Mt. (Taklimakan Desert)	TK1-1_3	15.9	2.8	35.7	8.6	11.2	25.8	2
TK1-1, Kunlun Mt. (Taklimakan Desert)	TK1-1_4	24.4	3.9	45.9	11.2	6.5	8.1	1
TK1-1, Kunlun Mt. (Taklimakan Desert)	TK1-1_5	24.7	3.3	42.6	9.6	8.1	11.8	1
TK1-1, Kunlun Mt. (Taklimakan Desert)	TK1-1_6	40.4	4.1	33.5	8.3	7.4	6.3	1
TK1-1, Kunlun Mt. (Taklimakan Desert)	TK1-1_7	29.0	3.9	44.2	11.2	5.1	6.7	1
TK1-1, Kunlun Mt. (Taklimakan Desert)	TK1-1_8	22.7	3.1	45.9	10.3	8.0	9.9	1
TK1-1, Kunlun Mt. (Taklimakan Desert)	TK1-1_9	32.2	3.9	37.2	11.3	6.4	9.0	1
TK1-1, Kunlun Mt. (Taklimakan Desert)	TK1-1_10	28.9	3.4	40.2	11.5	6.9	9.1	1
TK1-1, Kunlun Mt. (Taklimakan Desert)	TK1-1_11	27.7	3.8	43.1	10.8	6.2	8.3	1
TK1-1, Kunlun Mt. (Taklimakan Desert)	TK1-1_12	22.8	2.9	45.8	10.7	8.7	9.1	1
TK1-1, Kunlun Mt. (Taklimakan Desert)	TK1-1_13	22.5	3.2	46.2	10.7	7.5	9.9	1
TK1-1, Kunlun Mt. (Taklimakan Desert)	TK1-1_14	24.9	3.2	44.3	11.4	7.2	8.9	1
TK1-1, Kunlun Mt. (Taklimakan Desert)	TK1-1_15	7.0	1.6	15.7	5.6	18.8	51.3	3
TK1-1, Kunlun Mt. (Taklimakan Desert)	TK1-1_16	12.8	2.3	26.7	6.7	15.3	36.2	2
TK1-1, Kunlun Mt. (Taklimakan Desert)	TK1-1_17	27.8	3.8	39.1	11.3	8.0	10.1	1
TK1-1, Kunlun Mt. (Taklimakan Desert)	TK1-1_18	28.2	3.5	41.6	11.1	7.1	8.5	1
TK1-1, Kunlun Mt. (Taklimakan Desert)	TK1-1_19	29.1	3.7	42.1	10.6	6.0	8.6	1
TK1-1, Kunlun Mt. (Taklimakan Desert)	TK1-1_20	16.6	2.4	32.0	9.7	13.7	25.6	2
TK1-1, Kunlun Mt. (Taklimakan Desert)	TK1-1_21	22.8	3.3	43.2	11.6	7.9	11.2	1
TK1-1, Kunlun Mt. (Taklimakan Desert)	TK1-1_22	20.3	3.0	32.0	10.3	14.0	20.3	2
TK1-1, Kunlun Mt. (Taklimakan Desert)	TK1-1_23	12.8	2.2	21.6	6.5	16.1	40.7	2
TK1-1, Kunlun Mt. (Taklimakan Desert)	TK1-1_24	26.7	3.3	40.4	11.8	8.5	9.4	1
TK1-1, Kunlun Mt. (Taklimakan Desert)	TK1-1_25	12.1	2.2	23.1	7.1	17.0	38.5	2
TK1-1, Kunlun Mt. (Taklimakan Desert)	TK1-1_26	28.5	3.4	36.9	11.4	9.2	10.6	1
TK1-1, Kunlun Mt. (Taklimakan Desert)	TK1-1_27	21.5	3.3	39.9	11.9	13.0	10.4	1
TK1-1, Kunlun Mt. (Taklimakan Desert)	TK1-1_28	27.4	3.4	34.6	11.9	10.1	12.7	1
TK1-1, Kunlun Mt. (Taklimakan Desert)	TK1-1_29	28.9	3.6	37.9	9.7	7.4	12.5	1
TK1-1, Kunlun Mt. (Taklimakan Desert)	TK1-1_30	20.1	3.1	41.6	10.3	9.1	15.8	1
TK1-1, Kunlun Mt. (Taklimakan Desert)	TK1-1_31	22.3	3.4	46.1	10.6	7.9	9.6	1
TK1-1, Kunlun Mt. (Taklimakan Desert)	TK1-1_32	20.4	2.9	45.0	10.7	9.2	11.8	1
TK1-1, Kunlun Mt. (Taklimakan Desert)	TK1-1_33	0.8	0.7	0.9	0.0	7.5	90.1	3
TK1-1, Kunlun Mt. (Taklimakan Desert)	TK1-1_34	21.0	3.2	44.6	11.1	9.4	10.8	1
TK1-1, Kunlun Mt. (Taklimakan Desert)	TK1-1_35	21.8	3.3	46.8	10.0	8.0	10.0	1
TK1-1, Kunlun Mt. (Taklimakan Desert)	TK1-1_36	18.7	3.5	37.3	9.4	12.2	19.0	2
TK1-1, Kunlun Mt. (Taklimakan Desert)	TK1-1_37	20.8	3.6	43.1	10.8	9.8	11.8	1
TK1-1, Kunlun Mt. (Taklimakan Desert)	TK1-1_38	19.1	3.1	42.8	11.9	9.9	13.3	1
TK1-1, Kunlun Mt. (Taklimakan Desert)	TK1-1_39	20.7	1.4	23.3	17.3	13.7	23.6	2
TK1-1, Kunlun Mt. (Taklimakan Desert)	TK1-1_40	17.6	2.8	32.6	12.3	13.5	21.1	2
TK1-1, Kunlun Mt. (Taklimakan Desert)	TK1-1_41	1.0	0.0	0.0	8.8	41.7	48.5	3
TK1-1, Kunlun Mt. (Taklimakan Desert)	TK1-1_42	17.6	2.8	32.6	12.3	13.5	21.1	2
TK1-1, Kunlun Mt. (Taklimakan Desert)	TK1-1_43	21.9	2.7	46.4	15.8	6.0	7.1	1
TK1-1, Kunlun Mt. (Taklimakan Desert)	TK1-1_44	19.4	3.0	42.7	12.8	9.3	12.9	1
TK1-1, Kunlun Mt. (Taklimakan Desert)	TK1-1_45	4.4	1.4	12.6	5.0	20.1	56.4	3
TK1-1, Kunlun Mt. (Taklimakan Desert)	TK1-1_46	15.1	2.4	27.6	11.9	15.9	27.2	2
TK1-2, Kunlun Mt. (Taklimakan Desert)	TK1-2_1	3.1	1.8	8.7	8.9	25.3	52.2	3
TK1-2, Kunlun Mt. (Taklimakan Desert)	TK1-2_2	17.2	2.9	39.7	12.1	11.4	16.8	1
TK1-2, Kunlun Mt. (Taklimakan Desert)	TK1-2_3	26.8	3.1	45.7	10.9	6.7	6.7	1
TK1-2, Kunlun Mt. (Taklimakan Desert)	TK1-2_4	9.3	1.8	25.4	7.2	15.5	40.7	2
TK1-2, Kunlun Mt. (Taklimakan Desert)	TK1-2_5	22.3	3.0	38.3	10.7	10.0	15.6	1
TK1-2, Kunlun Mt. (Taklimakan Desert)	TK1-2_6	18.5	2.9	38.8	10.3	10.7	18.8	2
TK1-2, Kunlun Mt. (Taklimakan Desert)	TK1-2_7	26.7	3.2	46.0	10.0	6.4	7.7	1
TK1-2, Kunlun Mt. (Taklimakan Desert)	TK1-2_8	19.9	3.2	43.6	10.1	10.3	13.0	1
TK1-2, Kunlun Mt. (Taklimakan Desert)	TK1-2_9	21.4	3.1	44.1	10.8	9.3	11.3	1
TK1-2, Kunlun Mt. (Taklimakan Desert)	TK1-2_10	14.8	2.7	32.2	9.1	12.4	28.8	2
TK1-2, Kunlun Mt. (Taklimakan Desert)	TK1-2_11	24.6	3.3	49.5	10.2	6.3	6.1	1
TK1-2, Kunlun Mt. (Taklimakan Desert)	TK1-2_12	21.8	2.9	42.1	11.2	10.6	11.4	1
TK1-2, Kunlun Mt. (Taklimakan Desert)	TK1-2_13	6.1	0.9	9.2	5.9	23.1	54.8	3
TK1-2, Kunlun Mt. (Taklimakan Desert)	TK1-2_14	16.7	2.4	32.5	8.2	12.3	27.8	2
TK1-2, Kunlun Mt. (Taklimakan Desert)	TK1-2_15	21.8	2.7	40.6	10.0	7.9	16.9	1
TK1-2, Kunlun Mt. (Taklimakan Desert)	TK1-2_16	16.5	2.7	34.4	9.2	12.1	25.0	2
TK1-2, Kunlun Mt. (Taklimakan Desert)	TK1-2_17	19.0	3.1	45.1	11.8	8.4	12.6	1
TK1-2, Kunlun Mt. (Taklimakan Desert)	TK1-2_18	23.9	3.4	48.2	11.4	7.2	5.9	1
TK1-2, Kunlun Mt. (Taklimakan Desert)	TK1-2_19	22.3	3.1	42.2	9.0	8.3	15.1	1
TK1-2, Kunlun Mt. (Taklimakan Desert)	TK1-2_20	22.4	3.4	45.2	9.1	7.8	12.0	1
TK1-2, Kunlun Mt. (Taklimakan Desert)	TK1-2_21	31.3	3.7	44.2	11.2	3.9	5.7	1
TK1-2, Kunlun Mt. (Taklimakan Desert)	TK1-2_22	23.9	3.3	39.3	11.5	12.1	9.9	1
TK1-2, Kunlun Mt. (Taklimakan Desert)	TK1-2_23	4.7	3.4	13.0	15.4	25.6	37.9	2
TK1-2, Kunlun Mt. (Taklimakan Desert)	TK1-2_24	22.4	3.6	31.1	14.3	13.7	15.0	1
TK1-2, Kunlun Mt. (Taklimakan Desert)	TK1-2_25	21.1	3.8	46.5	9.8	7.1	11.6	1
TK1-2, Kunlun Mt. (Taklimakan Desert)	TK1-2_26	12.0	2.1	29.9	9.3	16.0	30.7	2
TK1-2, Kunlun Mt. (Taklimakan Desert)	TK1-2_27	19.3	2.9	41.8	10.6	11.4	14.1	1
TK1-2, Kunlun Mt. (Taklimakan Desert)	TK1-2_28	18.4	2.9	39.5	12.4	13.2	13.6	1

TK1-2, Kunlun Mt. (Taklimakan Desert)	TK1-2_29	6.6	1.6	17.7	6.1	19.3	48.8	3
TK1-2, Kunlun Mt. (Taklimakan Desert)	TK1-2_30	17.0	2.9	41.5	10.5	9.1	19.0	2
TK1-2, Kunlun Mt. (Taklimakan Desert)	TK1-2_31	20.8	3.4	42.5	12.3	10.3	10.8	1
TK1-2, Kunlun Mt. (Taklimakan Desert)	TK1-2_32	22.8	3.2	47.0	12.1	7.7	7.3	1
TK1-2, Kunlun Mt. (Taklimakan Desert)	TK1-2_33	9.3	1.7	30.4	3.2	7.2	48.2	2
TK1-2, Kunlun Mt. (Taklimakan Desert)	TK1-2_34	21.7	2.6	42.7	12.0	10.5	10.5	1
TK1-2, Kunlun Mt. (Taklimakan Desert)	TK1-2_35	18.3	3.4	39.7	11.1	12.0	15.4	1
TK1-2, Kunlun Mt. (Taklimakan Desert)	TK1-2_36	29.2	0.3	21.2	11.6	13.8	23.8	2
TK1-2, Kunlun Mt. (Taklimakan Desert)	TK1-2_37	22.1	3.1	47.9	11.8	7.3	7.8	1
TK1-2, Kunlun Mt. (Taklimakan Desert)	TK1-2_38	19.0	3.4	39.0	11.0	11.9	15.7	1
TK1-2, Kunlun Mt. (Taklimakan Desert)	TK1-2_39	16.6	3.0	32.1	12.0	15.4	20.9	2
TK1-2, Kunlun Mt. (Taklimakan Desert)	TK1-2_40	15.6	2.6	33.3	8.3	13.8	26.5	2
TK1-2, Kunlun Mt. (Taklimakan Desert)	TK1-2_41	12.8	5.5	43.2	6.3	3.0	29.2	2
TK1-2, Kunlun Mt. (Taklimakan Desert)	TK1-2_42	17.8	2.8	39.6	9.9	11.1	18.7	2
TK1-2, Kunlun Mt. (Taklimakan Desert)	TK1-2_43	4.1	1.4	11.7	5.0	21.1	56.7	3
TK1-2, Kunlun Mt. (Taklimakan Desert)	TK1-2_44	22.3	4.0	39.6	11.5	11.0	11.5	1
TK1-2, Kunlun Mt. (Taklimakan Desert)	TK1-2_45	25.4	3.5	49.9	9.9	5.5	5.8	1
TK1-2, Kunlun Mt. (Taklimakan Desert)	TK1-2_46	29.2	5.5	43.6	13.3	6.0	2.5	1
TK1-2, Kunlun Mt. (Taklimakan Desert)	TK1-2_47	20.7	3.1	44.5	11.2	8.6	12.0	1
TK1-2, Kunlun Mt. (Taklimakan Desert)	TK1-2_48	23.3	3.3	45.4	11.4	8.5	8.1	1
TK1-2, Kunlun Mt. (Taklimakan Desert)	TK1-2_49	18.3	2.8	40.9	10.8	11.6	15.6	1
TK1-2, Kunlun Mt. (Taklimakan Desert)	TK1-2_50	20.2	3.1	46.2	11.0	8.9	10.6	1
TK2, Kunlun Mt. (Taklimakan Desert)	TK2_1	22.0	1.9	29.7	8.1	14.1	24.1	2
TK2, Kunlun Mt. (Taklimakan Desert)	TK2_2	15.7	2.3	31.9	9.3	14.0	26.8	2
TK2, Kunlun Mt. (Taklimakan Desert)	TK2_3	20.8	2.9	37.4	12.8	12.7	13.4	1
TK2, Kunlun Mt. (Taklimakan Desert)	TK2_4	24.3	3.6	45.0	12.3	7.7	6.9	1
TK2, Kunlun Mt. (Taklimakan Desert)	TK2_5	14.6	2.6	29.5	13.1	18.3	22.0	2
TK2, Kunlun Mt. (Taklimakan Desert)	TK2_6	23.8	3.5	46.7	11.9	7.0	7.1	1
TK2, Kunlun Mt. (Taklimakan Desert)	TK2_7	16.8	3.1	35.0	12.0	13.0	20.0	2
TK2, Kunlun Mt. (Taklimakan Desert)	TK2_8	24.9	3.3	44.6	12.3	7.1	7.9	1
TK2, Kunlun Mt. (Taklimakan Desert)	TK2_9	17.1	3.1	36.4	14.0	15.2	14.2	2
TK2, Kunlun Mt. (Taklimakan Desert)	TK2_10	10.4	2.0	24.4	9.7	16.4	37.1	2
TK2, Kunlun Mt. (Taklimakan Desert)	TK2_11	13.1	2.0	26.9	9.0	17.6	31.4	2
TK2, Kunlun Mt. (Taklimakan Desert)	TK2_12	20.8	3.1	42.1	11.8	10.3	11.8	1
TK2, Kunlun Mt. (Taklimakan Desert)	TK2_13	18.2	3.5	33.6	12.7	13.5	18.4	2
TK2, Kunlun Mt. (Taklimakan Desert)	TK2_14	30.9	4.1	42.6	9.6	5.6	7.2	1
TK2, Kunlun Mt. (Taklimakan Desert)	TK2_15	30.1	3.6	34.8	8.2	7.6	15.8	1
TK2, Kunlun Mt. (Taklimakan Desert)	TK2_16	28.5	3.0	39.6	11.8	6.0	11.0	1
TK2, Kunlun Mt. (Taklimakan Desert)	TK2_17	31.3	2.9	32.1	14.6	7.3	11.8	1
TK2, Kunlun Mt. (Taklimakan Desert)	TK2_18	22.0	3.4	43.0	9.6	8.8	13.1	1
TK2, Kunlun Mt. (Taklimakan Desert)	TK2_19	29.5	3.0	35.6	17.2	8.3	6.4	1
TK2, Kunlun Mt. (Taklimakan Desert)	TK2_20	14.4	3.4	28.1	7.2	14.4	32.5	2
TK2, Kunlun Mt. (Taklimakan Desert)	TK2_21	28.3	4.2	45.2	8.6	6.3	7.3	1
TK2, Kunlun Mt. (Taklimakan Desert)	TK2_22	15.3	3.1	25.5	6.9	15.1	34.0	2
TK2, Kunlun Mt. (Taklimakan Desert)	TK2_23	30.8	4.3	45.9	8.7	5.6	4.6	1
TK2, Kunlun Mt. (Taklimakan Desert)	TK2_24	30.2	3.8	44.7	8.6	2.3	10.3	1
TK2, Kunlun Mt. (Taklimakan Desert)	TK2_25	14.4	3.5	32.5	6.4	8.8	34.3	2
TK2, Kunlun Mt. (Taklimakan Desert)	TK2_26	24.8	4.9	47.1	7.9	7.4	7.9	1
TK2, Kunlun Mt. (Taklimakan Desert)	TK2_27	25.8	3.5	45.7	10.2	6.3	8.6	1
TK2, Kunlun Mt. (Taklimakan Desert)	TK2_28	21.5	2.9	34.8	11.3	10.5	19.1	2
TK2, Kunlun Mt. (Taklimakan Desert)	TK2_29	22.8	2.9	33.2	12.0	11.4	17.7	1
TK2, Kunlun Mt. (Taklimakan Desert)	TK2_30	37.5	4.1	32.1	10.4	7.8	8.1	1
TK2, Kunlun Mt. (Taklimakan Desert)	TK2_31	25.0	3.9	43.9	9.9	8.5	8.8	1
TK2, Kunlun Mt. (Taklimakan Desert)	TK2_32	9.2	1.8	19.2	5.7	19.4	44.8	3
TK2, Kunlun Mt. (Taklimakan Desert)	TK2_33	10.5	1.8	18.1	5.7	16.3	47.6	3
TK2, Kunlun Mt. (Taklimakan Desert)	TK2_34	14.1	2.9	23.6	9.5	16.9	33.0	2
TK2, Kunlun Mt. (Taklimakan Desert)	TK2_35	22.4	3.1	37.8	7.1	7.3	22.3	2
TK2, Kunlun Mt. (Taklimakan Desert)	TK2_36	28.7	3.4	46.6	9.9	4.4	7.0	1
TK2, Kunlun Mt. (Taklimakan Desert)	TK2_37	39.5	4.8	33.6	9.4	6.2	6.5	1
TK2, Kunlun Mt. (Taklimakan Desert)	TK2_38	29.2	3.1	38.4	11.9	7.8	9.7	1
TK2, Kunlun Mt. (Taklimakan Desert)	TK2_39	22.9	3.3	45.1	9.5	9.4	9.8	1
TK2, Kunlun Mt. (Taklimakan Desert)	TK2_40	19.0	2.7	35.0	8.6	12.6	22.1	2
TK2, Kunlun Mt. (Taklimakan Desert)	TK2_41	17.1	2.8	34.8	7.3	11.2	26.9	2
TK2, Kunlun Mt. (Taklimakan Desert)	TK2_42	19.5	3.1	35.6	8.0	11.7	22.1	2
TK2, Kunlun Mt. (Taklimakan Desert)	TK2_43	24.8	3.2	46.0	10.0	8.6	7.5	1
TK2, Kunlun Mt. (Taklimakan Desert)	TK2_44	0.2	1.7	9.7	0.0	2.2	86.1	3
TK2, Kunlun Mt. (Taklimakan Desert)	TK2_45	17.7	2.7	35.4	8.8	12.6	22.7	2
TK2, Kunlun Mt. (Taklimakan Desert)	TK2_46	27.0	4.3	45.1	10.0	4.6	9.1	1
TK2, Kunlun Mt. (Taklimakan Desert)	TK2_47	25.6	3.6	41.4	9.1	9.6	10.8	1
TK2, Kunlun Mt. (Taklimakan Desert)	TK2_48	13.6	2.4	27.6	6.0	16.3	34.1	2
GD1, Gobi Desert	GD1_1	11.5	1.9	24.7	8.9	18.3	34.8	2
GD1, Gobi Desert	GD1_2	20.7	2.7	31.4	10.9	12.5	21.8	2
GD1, Gobi Desert	GD1_3	19.6	2.4	31.6	10.2	10.0	26.2	2
GD1, Gobi Desert	GD1_4	17.4	2.4	35.4	8.2	12.7	23.8	2
GD1, Gobi Desert	GD1_5	15.9	1.9	32.6	10.7	12.9	25.9	2
GD1, Gobi Desert	GD1_6	22.4	2.5	39.2	10.7	8.1	17.1	1
GD1, Gobi Desert	GD1_7	31.8	1.6	44.7	9.8	4.8	7.3	1
GD1, Gobi Desert	GD1_8	36.1	0.9	25.1	11.9	10.6	15.5	1
GD1, Gobi Desert	GD1_9	18.6	3.3	27.7	9.7	12.2	28.5	2
GD1, Gobi Desert	GD1_10	23.9	2.6	38.8	11.2	8.4	15.2	1
GD1, Gobi Desert	GD1_11	36.5	3.0	33.9	11.4	6.3	9.0	1
GD1, Gobi Desert	GD1_12	26.5	3.3	46.4	9.7	5.3	8.8	1
GD1, Gobi Desert	GD1_13	9.7	3.9	21.6	8.7	19.2	37.0	2
GD1, Gobi Desert	GD1_14	29.2	2.5	36.0	13.0	6.0	13.4	1
GD1, Gobi Desert	GD1_15	25.3	2.8	45.6	9.8	6.0	10.4	1
GD1, Gobi Desert	GD1_16	25.4	3.7	37.2	11.5	8.3	13.9	1
GD1, Gobi Desert	GD1_17	14.0	2.2	24.1	7.4	15.3	37.0	2
GD1, Gobi Desert	GD1_18	22.2	3.2	45.8	14.1	6.3	8.5	1
GD1, Gobi Desert	GD1_19	27.8	2.3	37.9	9.6	6.7	15.7	1

GD1, Gobi Desert	GD1_20	29.7	2.0	39.7	11.4	4.8	12.4	1
GD1, Gobi Desert	GD1_21	14.7	2.2	30.9	6.2	14.0	32.1	2
GD1, Gobi Desert	GD1_22	22.2	3.3	44.1	9.8	5.7	15.0	1
GD1, Gobi Desert	GD1_23	26.4	3.1	48.3	9.9	4.5	7.8	1
GD1, Gobi Desert	GD1_24	27.1	3.0	46.1	10.7	3.5	9.6	1
GD1, Gobi Desert	GD1_25	26.0	1.7	18.8	9.1	12.3	32.1	2
GD1, Gobi Desert	GD1_26	25.3	2.6	42.7	10.0	4.9	14.5	1
GD1, Gobi Desert	GD1_27	5.0	1.1	9.5	3.9	19.6	61.0	3
GD1, Gobi Desert	GD1_28	13.1	2.2	29.6	8.7	16.7	29.7	2
GD1, Gobi Desert	GD1_29	14.6	3.2	24.8	10.8	15.5	31.2	2
GD1, Gobi Desert	GD1_30	20.7	3.6	49.5	8.6	6.0	11.6	1
GD1, Gobi Desert	GD1_31	19.5	3.2	9.3	13.5	26.7	27.9	2
GD1, Gobi Desert	GD1_32	21.2	3.5	38.4	10.1	10.7	16.2	1
GD1, Gobi Desert	GD1_33	21.2	3.5	45.7	10.7	8.3	10.7	1
GD1, Gobi Desert	GD1_34	21.2	2.7	43.9	10.2	7.6	14.3	1
GD1, Gobi Desert	GD1_35	24.6	3.4	45.4	9.4	5.9	11.3	1
GD1, Gobi Desert	GD1_36	19.8	2.6	29.9	13.5	14.2	20.0	2
GD1, Gobi Desert	GD1_37	3.2	2.0	6.1	9.0	26.1	53.5	3
GD1, Gobi Desert	GD1_38	21.2	2.7	38.6	13.7	10.1	13.7	1
GD1, Gobi Desert	GD1_39	22.1	2.9	46.3	14.3	5.7	8.8	1
GD1, Gobi Desert	GD1_40	10.9	2.3	17.0	10.3	19.0	40.5	2
GD1, Gobi Desert	GD1_41	27.3	3.5	22.6	10.6	13.0	23.1	2
GD1, Gobi Desert	GD1_42	22.2	2.7	38.5	13.3	9.9	13.3	1
GD1, Gobi Desert	GD1_43	17.7	1.5	12.5	10.8	22.8	34.7	2
GD1, Gobi Desert	GD1_44	0.7	0.6	0.0	6.4	24.2	68.2	3
GD1, Gobi Desert	GD1_45	22.4	7.7	17.2	9.3	10.7	32.7	2
GD1, Gobi Desert	GD1_46	23.6	3.1	43.2	13.7	5.5	10.9	1
GD1, Gobi Desert	GD1_47	0.2	0.5	0.0	4.9	23.7	70.7	3
GD1, Gobi Desert	GD1_48	0.0	0.1	0.0	4.1	23.1	72.7	3
GD1, Gobi Desert	GD1_49	22.9	2.7	38.8	12.2	8.7	14.7	1
GD1, Gobi Desert	GD1_50	13.5	3.8	43.2	8.1	0.5	30.9	2
GD2, Gobi Desert	GD2_1	15.2	2.4	37.7	11.9	10.3	22.4	2
GD2, Gobi Desert	GD2_2	26.2	1.8	8.6	7.7	18.6	37.1	2
GD2, Gobi Desert	GD2_3	1.3	0.8	5.2	3.2	20.3	69.1	3
GD2, Gobi Desert	GD2_4	1.3	0.0	4.9	2.4	20.6	70.8	3
GD2, Gobi Desert	GD2_5	53.3	0.0	2.1	13.0	12.7	19.0	2
GD2, Gobi Desert	GD2_6	10.9	0.9	7.7	6.7	23.8	50.1	3
GD2, Gobi Desert	GD2_7	27.6	3.9	43.5	11.5	4.7	8.8	1
GD2, Gobi Desert	GD2_8	18.7	2.4	36.4	11.9	9.0	21.5	2
GD2, Gobi Desert	GD2_9	16.5	2.8	39.5	12.9	10.3	18.0	2
GD2, Gobi Desert	GD2_10	0.4	0.7	1.5	4.4	19.1	74.0	3
GD2, Gobi Desert	GD2_11	4.9	1.6	15.0	6.1	15.5	56.9	3
GD2, Gobi Desert	GD2_12	10.2	2.3	25.8	8.8	14.9	38.1	2
GD2, Gobi Desert	GD2_13	23.5	3.1	45.5	14.5	6.9	6.4	1
GD2, Gobi Desert	GD2_14	20.6	2.8	46.2	12.4	7.0	11.0	1
GD2, Gobi Desert	GD2_15	22.9	3.6	38.5	11.2	10.2	13.6	1
GD2, Gobi Desert	GD2_16	8.2	5.8	16.3	6.4	24.4	38.8	3
GD2, Gobi Desert	GD2_17	6.1	4.1	27.7	11.4	19.8	30.9	2
GD2, Gobi Desert	GD2_18	17.6	2.9	45.7	11.5	7.5	14.8	1
GD2, Gobi Desert	GD2_19	23.2	2.8	41.0	12.1	7.7	13.2	1
GD2, Gobi Desert	GD2_20	33.2	3.5	32.7	11.9	7.4	11.3	1
GD2, Gobi Desert	GD2_21	18.6	2.7	36.9	11.8	11.1	19.0	2
GD2, Gobi Desert	GD2_22	1.2	0.7	3.7	3.4	22.5	68.4	3
GD2, Gobi Desert	GD2_23	21.9	3.2	36.5	12.4	8.7	17.3	1
GD2, Gobi Desert	GD2_24	17.9	2.7	39.2	12.4	6.7	21.1	2
GD2, Gobi Desert	GD2_25	24.9	2.6	46.1	12.4	5.1	8.9	1
GD2, Gobi Desert	GD2_26	26.9	0.0	0.0	4.6	19.1	49.3	2
GD2, Gobi Desert	GD2_27	21.7	2.7	42.6	13.5	6.2	13.3	1
GD2, Gobi Desert	GD2_28	6.1	1.6	12.9	6.6	18.0	54.8	3
GD2, Gobi Desert	GD2_29	16.5	3.3	39.4	11.4	9.6	19.7	2
GD2, Gobi Desert	GD2_30	35.7	3.3	41.1	10.2	3.0	6.6	1
GD2, Gobi Desert	GD2_31	25.9	3.0	41.8	13.3	6.4	9.6	1
GD2, Gobi Desert	GD2_32	26.4	4.2	13.4	8.1	13.7	34.1	2
GD2, Gobi Desert	GD2_33	42.9	0.0	7.6	13.9	13.1	22.6	2
GD2, Gobi Desert	GD2_34	8.1	1.9	19.7	6.4	12.6	51.3	3
GD2, Gobi Desert	GD2_35	20.5	3.1	47.1	11.8	6.3	11.2	1
GD2, Gobi Desert	GD2_36	14.8	2.6	33.0	9.6	11.7	28.3	2
GD2, Gobi Desert	GD2_37	4.2	1.2	11.5	4.8	19.3	59.1	3
GD2, Gobi Desert	GD2_38	23.8	2.9	43.4	12.8	6.1	11.1	1
GD2, Gobi Desert	GD2_39	30.5	2.6	30.3	12.4	5.5	18.7	1
GD2, Gobi Desert	GD2_40	27.0	3.3	27.3	14.2	7.7	20.4	1
GD2, Gobi Desert	GD2_41	14.5	2.1	29.4	9.3	13.2	31.6	2
GD2, Gobi Desert	GD2_42	18.8	2.6	38.5	11.6	9.1	19.4	2
GD2, Gobi Desert	GD2_43	3.0	1.1	9.5	3.4	19.2	63.9	3
GD2, Gobi Desert	GD2_44	4.6	1.2	12.9	4.4	18.0	58.9	3
GD2, Gobi Desert	GD2_45	4.8	1.0	7.5	4.6	21.1	60.9	3
GD2, Gobi Desert	GD2_46	21.8	3.2	23.3	7.3	11.0	33.4	2
GD2, Gobi Desert	GD2_47	14.3	2.2	29.7	9.0	14.2	30.6	2
GD2, Gobi Desert	GD2_48	1.1	0.7	3.5	3.0	21.6	70.0	3
GD2, Gobi Desert	GD2_49	11.0	1.8	24.7	8.7	14.3	39.6	2
GD2, Gobi Desert	GD2_50	26.9	3.0	44.4	13.6	5.2	6.9	1
GD2, Gobi Desert	GD2_51	24.2	3.4	47.5	12.5	4.1	8.4	1
GD2, Gobi Desert	GD2_52	24.7	2.9	31.1	21.6	12.4	7.2	1
GD2, Gobi Desert	GD2_53	4.6	0.5	0.0	2.7	13.9	78.3	3
GD2, Gobi Desert	GD2_54	19.9	3.1	40.3	12.0	9.7	14.9	1
GD2, Gobi Desert	GD2_55	28.6	3.7	31.1	14.1	7.7	14.9	1
GD2, Gobi Desert	GD2_56	31.9	4.0	42.3	11.0	7.2	3.7	1
GD2, Gobi Desert	GD2_57	23.3	2.8	42.3	13.7	7.2	10.7	1
GD2, Gobi Desert	GD2_58	19.7	3.2	33.7	12.1	10.9	20.4	2

GD2, Gobi Desert	GD2_59	28.0	3.4	51.6	9.7	2.1	5.2	1
GD2, Gobi Desert	GD2_60	32.9	1.3	39.1	14.6	3.6	8.5	1
GD3, Gobi Desert	GD3_1	8.8	3.1	32.8	5.1	13.7	36.6	2
GD3, Gobi Desert	GD3_2	28.0	3.0	34.0	10.4	11.7	12.9	1
GD3, Gobi Desert	GD3_3	4.7	1.3	13.5	5.2	18.3	57.1	3
GD3, Gobi Desert	GD3_4	9.1	7.6	44.6	14.7	0.0	24.1	2
GD3, Gobi Desert	GD3_5	22.1	2.8	42.5	11.4	8.9	12.3	1
GD3, Gobi Desert	GD3_6	21.6	3.5	36.0	13.1	9.6	16.2	1
GD3, Gobi Desert	GD3_7	21.2	3.0	46.6	10.8	7.4	11.0	1
GD3, Gobi Desert	GD3_8	26.6	3.4	48.4	9.6	6.0	6.1	1
GD3, Gobi Desert	GD3_9	21.8	3.2	42.1	9.3	9.8	13.7	1
GD3, Gobi Desert	GD3_10	18.4	3.3	29.4	16.1	13.3	19.5	2
GD3, Gobi Desert	GD3_11	21.9	2.9	43.8	9.1	9.1	13.4	1
GD3, Gobi Desert	GD3_12	28.1	4.2	44.9	8.2	6.9	7.8	1
GD3, Gobi Desert	GD3_13	23.0	3.5	45.9	11.9	7.2	8.6	1
GD3, Gobi Desert	GD3_14	16.1	2.5	33.7	7.5	11.8	28.4	2
GD3, Gobi Desert	GD3_15	21.1	3.3	47.2	11.7	7.7	9.0	1
GD3, Gobi Desert	GD3_16	21.8	3.4	44.2	10.5	9.0	11.1	1
GD3, Gobi Desert	GD3_17	23.0	3.1	44.2	9.9	9.3	10.6	1
GD3, Gobi Desert	GD3_18	25.9	3.6	41.2	10.0	7.7	11.6	1
GD3, Gobi Desert	GD3_19	23.6	3.7	30.3	9.3	11.3	21.8	2
GD3, Gobi Desert	GD3_20	21.5	2.4	35.1	9.8	10.3	20.9	2
GD3, Gobi Desert	GD3_21	21.1	3.4	47.2	11.2	8.0	9.1	1
GD3, Gobi Desert	GD3_22	6.4	1.8	14.0	7.1	21.6	49.1	3
GD3, Gobi Desert	GD3_23	20.7	3.0	45.7	9.8	8.8	12.0	1
GD3, Gobi Desert	GD3_24	19.7	2.8	42.6	11.6	10.4	12.9	1
GD3, Gobi Desert	GD3_25	20.5	2.8	46.8	10.7	7.0	12.1	1
GD3, Gobi Desert	GD3_26	19.0	2.8	43.0	10.7	10.0	14.5	1
GD3, Gobi Desert	GD3_27	18.0	2.9	39.9	11.0	11.7	16.5	1
GD3, Gobi Desert	GD3_28	19.5	3.2	38.6	11.1	10.4	17.1	1
GD3, Gobi Desert	GD3_29	15.8	2.3	34.4	7.7	14.2	25.5	2
GD3, Gobi Desert	GD3_30	22.2	3.2	47.5	10.3	7.2	9.7	1
GD3, Gobi Desert	GD3_31	21.8	3.2	44.4	11.1	7.0	12.5	1
GD3, Gobi Desert	GD3_32	22.7	3.1	44.0	12.3	8.4	9.4	1
GD3, Gobi Desert	GD3_33	23.6	3.1	46.2	10.0	7.0	10.0	1
GD3, Gobi Desert	GD3_34	20.6	3.1	44.2	12.5	8.4	11.2	1
GD3, Gobi Desert	GD3_35	5.5	1.5	14.9	4.9	18.6	54.5	3
GD3, Gobi Desert	GD3_36	16.1	2.7	38.4	11.5	12.6	18.7	2
GD3, Gobi Desert	GD3_37	23.7	2.8	39.2	12.9	9.9	11.5	1
GD3, Gobi Desert	GD3_38	4.9	1.4	14.9	3.8	18.2	56.8	3
GD3, Gobi Desert	GD3_39	19.1	2.7	38.3	11.6	11.8	16.5	1
GD3, Gobi Desert	GD3_40	6.8	1.4	16.8	5.3	17.8	51.9	3
GD3, Gobi Desert	GD3_41	15.4	3.0	37.2	10.3	11.4	22.7	2
GD3, Gobi Desert	GD3_42	13.4	2.6	28.1	9.0	13.8	33.0	2
GD3, Gobi Desert	GD3_43	6.4	3.1	11.3	7.6	24.2	47.4	3
GD3, Gobi Desert	GD3_44	19.1	3.0	42.7	10.9	9.1	15.2	1
GD3, Gobi Desert	GD3_45	20.0	3.1	40.5	9.7	11.0	15.7	1
GD3, Gobi Desert	GD3_46	8.8	1.9	21.8	6.2	16.5	44.8	3
GD3, Gobi Desert	GD3_47	17.4	2.7	37.0	9.2	11.9	21.7	2
GD3, Gobi Desert	GD3_48	23.5	3.4	46.7	9.5	8.0	9.0	1
GD4', Gobi Desert	GD4'_1	6.8	1.7	16.5	5.1	17.9	52.0	3
GD4', Gobi Desert	GD4'_2	19.3	3.1	43.6	11.1	10.2	12.7	1
GD4', Gobi Desert	GD4'_3	0.1	0.8	3.2	0.0	1.3	94.6	3
GD4', Gobi Desert	GD4'_4	5.3	1.8	13.4	7.4	20.7	51.4	3
GD4', Gobi Desert	GD4'_5	0.6	0.2	0.0	8.1	37.2	53.9	3
GD4', Gobi Desert	GD4'_6	12.9	2.1	18.8	5.9	16.9	43.3	2
GD4', Gobi Desert	GD4'_7	28.7	3.4	39.4	11.0	6.7	10.8	1
GD4', Gobi Desert	GD4'_8	20.1	3.0	39.6	9.0	7.8	20.4	2
GD4', Gobi Desert	GD4'_9	24.2	3.0	43.3	11.5	8.2	9.7	1
GD4', Gobi Desert	GD4'_10	16.3	2.8	27.8	8.1	11.1	33.8	2
GD4', Gobi Desert	GD4'_11	16.7	2.6	35.5	10.6	13.8	20.8	2
GD4', Gobi Desert	GD4'_12	16.5	2.4	13.9	7.2	18.0	42.0	2
GD4', Gobi Desert	GD4'_13	29.7	3.3	39.2	12.0	6.7	9.1	1
GD4', Gobi Desert	GD4'_14	5.8	0.6	0.0	4.8	30.3	58.5	3
GD4', Gobi Desert	GD4'_15	13.4	2.1	29.9	6.9	9.0	38.7	2
GD4', Gobi Desert	GD4'_16	11.9	2.0	25.9	7.5	12.9	39.7	2
GD4', Gobi Desert	GD4'_17	17.6	2.8	36.7	9.8	11.5	21.6	2
GD4', Gobi Desert	GD4'_18	17.9	2.9	40.6	9.0	10.6	18.9	2
GD4', Gobi Desert	GD4'_19	22.3	2.8	43.6	10.2	7.4	13.7	1
GD4', Gobi Desert	GD4'_20	6.8	1.5	16.5	5.1	18.7	51.4	3
GD4', Gobi Desert	GD4'_21	19.7	2.7	43.1	10.3	8.8	15.3	1
GD4', Gobi Desert	GD4'_22	11.1	1.9	25.3	6.4	14.5	40.9	2
GD4', Gobi Desert	GD4'_23	2.0	0.7	0.5	4.8	15.5	76.6	3
GD4', Gobi Desert	GD4'_24	42.6	5.2	38.1	7.3	1.3	5.5	1
GD4', Gobi Desert	GD4'_25	30.4	4.0	33.9	11.4	10.1	10.2	1
GD4', Gobi Desert	GD4'_26	25.0	3.6	43.7	10.1	7.5	10.0	1
GD4', Gobi Desert	GD4'_27	22.8	3.6	47.0	10.7	7.0	8.9	1
GD4', Gobi Desert	GD4'_28	22.8	3.3	40.4	11.6	8.8	13.0	1
GD4', Gobi Desert	GD4'_29	13.3	2.2	32.9	8.6	9.7	33.4	2
GD4', Gobi Desert	GD4'_30	16.5	2.6	35.0	12.0	15.6	18.3	2
GD4', Gobi Desert	GD4'_31	4.7	1.3	14.0	4.7	18.3	57.1	3
GD4', Gobi Desert	GD4'_32	20.2	2.8	42.4	10.4	10.1	14.0	1
GD4', Gobi Desert	GD4'_33	17.3	2.2	29.5	11.9	12.1	27.0	2
GD4', Gobi Desert	GD4'_34	1.0	0.8	4.3	3.3	23.1	67.6	3
GD4', Gobi Desert	GD4'_35	16.8	2.6	34.1	13.9	14.7	18.0	2
GD4', Gobi Desert	GD4'_36	6.2	2.0	14.7	7.5	18.2	51.5	3
GD4', Gobi Desert	GD4'_37	5.7	1.7	17.2	6.3	18.5	50.7	3
GD4', Gobi Desert	GD4'_38	15.9	1.7	20.6	11.3	16.9	33.7	2
GD4', Gobi Desert	GD4'_39	24.7	2.0	44.1	14.4	6.0	8.8	1

GD4', Gobi Desert	GD4'_40	12.6	0.9	9.8	9.5	21.2	45.9	3
GD4', Gobi Desert	GD4'_41	16.8	2.1	35.0	11.3	11.3	23.5	2
GD4', Gobi Desert	GD4'_42	14.9	2.5	23.8	14.8	18.9	25.1	2
GD4', Gobi Desert	GD4'_43	5.1	1.5	14.5	5.4	18.8	54.7	3
GD4', Gobi Desert	GD4'_44	20.1	2.8	37.7	11.3	10.5	17.5	1
GD4', Gobi Desert	GD4'_45	20.8	2.8	41.5	13.6	9.1	12.1	1
GD4', Gobi Desert	GD4'_46	20.0	3.0	39.6	11.6	10.4	15.4	1
GD4', Gobi Desert	GD4'_47	19.4	3.6	45.8	10.0	7.5	13.7	1
GD4', Gobi Desert	GD4'_48	28.1	3.4	46.0	8.9	6.3	7.4	1
GD4', Gobi Desert	GD4'_49	2.3	1.1	8.7	3.2	19.6	65.0	3
GD4', Gobi Desert	GD4'_50	13.0	3.4	28.9	12.1	15.4	27.1	2
GD4', Gobi Desert	GD4'_51	2.3	1.5	4.7	10.3	17.4	63.8	3
GD4', Gobi Desert	GD4'_52	24.3	4.1	37.3	13.2	7.3	13.8	1
GD4', Gobi Desert	GD4'_53	37.4	3.6	26.7	10.1	0.0	22.2	1
GD4', Gobi Desert	GD4'_54	20.7	1.9	20.6	5.8	7.5	43.4	2
GD4', Gobi Desert	GD4'_55	22.8	2.0	2.6	3.0	3.0	66.7	3
GD4', Gobi Desert	GD4'_56	30.7	4.2	24.5	14.5	10.5	15.6	1
GD4', Gobi Desert	GD4'_57	33.1	3.2	27.0	10.0	7.2	19.5	1
GD4', Gobi Desert	GD4'_58	24.8	3.3	50.2	7.5	4.4	9.9	1
GD4', Gobi Desert	GD4'_59	27.5	3.7	42.0	9.2	6.0	11.6	1
GD4', Gobi Desert	GD4'_60	8.6	4.4	37.3	0.0	3.4	46.3	2
GD4', Gobi Desert	GD4'_61	24.3	3.3	31.6	11.3	12.6	16.9	1
GD4', Gobi Desert	GD4'_62	27.7	4.2	48.0	6.7	5.5	7.9	1
GD4', Gobi Desert	GD4'_63	20.1	2.7	20.5	11.8	8.6	36.3	2
GD4', Gobi Desert	GD4'_64	9.1	2.0	17.0	8.3	9.3	54.3	3

Table S4. Emission component (EC) ratio determined for cluster 1–3.

	EC1	EC2	EC3	EC4	EC5	EC6
Cluster 1	25 ± 5%	3 ± 1%	39 ± 7%	12 ± 2%	9 ± 3%	12 ± 4%
Cluster 2	20 ± 9%	2 ± 1%	25 ± 11%	11 ± 3%	15 ± 4%	27 ± 8%
Cluster 3	4 ± 4%	1 ± 1%	8 ± 6%	5 ± 2%	21 ± 7%	61 ± 11%

Table S5.Cluster compositions of samples from the Asian deserts, Okhotsk Sea, Bering Sea, Japan, and station K2.

Station	Location	Measured particle number (n)	Cluster 1 (%)	Cluster 2 (%)	Cluster 3 (%)
TK1-1	Kunlun Mt. (Taklimakan)	46	67 (52–79)*	24 (13–38)	9 (3–20)
TK1-2	Kunlun Mt. (Taklimakan)	50	64 (50–75)	28 (17–41)	8 (3–19)
TK2	Kunlun Mt. (Taklimakan)	48	54 (40–67)	40 (26–53)	6 (2–17)
GD1	Gobi	50	52 (38–65)	38 (25–51)	10 (4–21)
GD2	Gobi	60	42 (30–54)	33 (22–46)	25 (15–37)
GD3	Gobi	48	62 (48–75)	23 (13–36)	15 (7–27)
GD4'	Gobi	64	41 (29–53)	33 (22–45)	26 (17–38)
B1	Okhotsk Sea	60	50 (37–62)	43 (31–56)	7 (2–16)
G2	Okhotsk Sea	60	25 (15–37)	62 (48–73)	13 (6–24)
5	Okhotsk Sea	47	23 (13–37)	49 (35–62)	28 (16–41)
6	Okhotsk Sea	50	16 (8–28)	52 (38–65)	32 (20–49)
23	Bering Sea	49	34 (22–48)	33 (21–46)	33 (21–46)
32	Bering Sea	54	37 (25–50)	43 (30–56)	20 (11–33)
33	Bering Sea	44	34 (21–49)	32 (19–46)	34 (22–50)
Station, sampling day, depth	Location	Measured particle number (n)	Cluster 1%	Cluster 2%	Cluster 3%
K2, 25 February 2003, 10 and 20m	Western subarctic Pacific	121	18 (12–26)	21 (14–29)	61 (51–69)
K2, 11 July 2003, 10 and 20m	Western subarctic Pacific	88	47 (36–57)	28 (19–38)	25 (16–35)
K2, 4 October 2003, 10 and 20m	Western subarctic Pacific	255	40 (33–46)	24 (18–30)	36 (30–43)
K2, 8 April 2004, 10 and 20m	Western subarctic Pacific	198	53 (42–61)	30 (23–36)	17 (12–22)
K2, 27 May 2004, 10m	Western subarctic Pacific	62	29 (18–41)	19 (11–31)	52 (39–63)
K2, 17 August 2004, 10 and 20m	Western subarctic Pacific	120	48 (38–56)	28 (20–37)	24 (17–33)
K2, 13 June 2006, 10 and 20m	Western subarctic Pacific	419	43 (38–48)	33 (28–37)	24 (19–28)
K2, 30 June 2006, 10 and 20m	Western subarctic Pacific	226	57 (50–63)	30 (24–36)	13 (9–18)
K2, 9 July 2006, 10 and 20m	Western subarctic Pacific	190	57 (50–64)	29 (22–36)	14 (9–19)
K2, 21 February 2021, 10m	Western subarctic Pacific	105	41 (31–51)	36 (27–46)	23 (9–31)
K2, 3 May 2022, 10 and 20m	Western subarctic Pacific	447	40 (35–44)	43 (38–47)	17 (14–21)
Nagasaki, 20–21 March 2010	Japan	73	59 (47–70)	34 (24–46)	7 (3–15)

*Numbers in parentheses show the 95% confidence interval.

Table S6. Relative abundances of emission components (EC1–EC6) and determined clusters for quartz particles from station K2 and Japan.

Station, Location	Quartz particle ID	Fractional area of each Gaussian curve (%)						Cluster
		EC1 (1.75 eV)	EC2 (1.9 eV)	EC3 (2.0 eV)	EC4 (2.2 eV)	EC5 (2.7 eV)	EC6 (2.9–3.2	
K2, 25 February 2003	2003Feb_1	17.7	2.8	41.1	12.6	10.7	15.1	1
K2, 25 February 2003	2003Feb_2	18.7	2.3	28.5	11.8	13.4	25.1	2
K2, 25 February 2003	2003Feb_3	21.3	2.6	44.0	14.4	7.6	10.1	1
K2, 25 February 2003	2003Feb_4	11.0	2.1	11.2	9.8	19.0	46.8	3
K2, 25 February 2003	2003Feb_5	14.4	2.1	30.4	14.2	13.1	25.8	2
K2, 25 February 2003	2003Feb_6	11.8	1.7	30.2	13.3	12.6	30.4	2
K2, 25 February 2003	2003Feb_7	15.1	2.2	24.0	11.3	14.3	33.2	2
K2, 25 February 2003	2003Feb_8	17.2	2.2	23.6	10.6	12.8	33.5	2
K2, 25 February 2003	2003Feb_9	5.6	1.0	9.8	9.8	21.1	52.8	3
K2, 25 February 2003	2003Feb_10	12.2	2.2	19.5	14.5	23.0	28.5	2
K2, 25 February 2003	2003Feb_11	0.1	0.2	0.0	3.8	22.1	73.8	3
K2, 25 February 2003	2003Feb_12	0.7	0.5	0.0	5.2	22.7	70.9	3
K2, 25 February 2003	2003Feb_13	21.8	2.6	34.7	13.9	9.0	17.9	1
K2, 25 February 2003	2003Feb_14	3.6	0.9	1.8	8.4	24.3	60.9	3
K2, 25 February 2003	2003Feb_15	3.0	0.8	2.2	7.5	22.4	64.1	3
K2, 25 February 2003	2003Feb_16	22.3	2.6	32.6	13.9	9.3	19.4	1
K2, 25 February 2003	2003Feb_17	4.5	0.8	8.9	6.8	19.7	59.2	3
K2, 25 February 2003	2003Feb_18	14.9	1.8	26.2	12.1	12.6	32.3	2
K2, 25 February 2003	2003Feb_19	2.3	0.7	2.2	6.1	23.1	65.5	3
K2, 25 February 2003	2003Feb_20	6.0	1.6	13.8	8.6	18.1	51.9	3
K2, 25 February 2003	2003Feb_21	19.4	2.5	34.4	12.9	10.2	20.6	1
K2, 25 February 2003	2003Feb_22	12.1	2.2	25.1	9.2	13.9	37.5	2
K2, 25 February 2003	2003Feb_23	17.5	2.4	37.9	14.2	9.8	18.3	1
K2, 25 February 2003	2003Feb_24	6.3	0.0	0.0	5.8	33.0	54.9	3
K2, 25 February 2003	2003Feb_25	7.4	2.3	16.4	9.9	19.4	44.6	3
K2, 25 February 2003	2003Feb_26	19.5	2.5	41.3	13.7	8.4	14.6	1
K2, 25 February 2003	2003Feb_27	14.9	2.1	26.9	11.1	14.7	30.2	2
K2, 25 February 2003	2003Feb_28	0.3	0.2	0.0	5.0	23.6	70.9	3
K2, 25 February 2003	2003Feb_29	17.0	2.5	35.5	13.8	10.1	21.2	1
K2, 25 February 2003	2003Feb_30	4.6	0.6	2.6	8.5	23.0	60.7	3
K2, 25 February 2003	2003Feb_31	19.7	2.1	22.7	10.2	13.5	31.8	2
K2, 25 February 2003	2003Feb_32	0.0	0.1	0.0	2.1	21.5	76.2	3
K2, 25 February 2003	2003Feb_33	15.5	1.6	28.0	10.9	12.1	31.9	2
K2, 25 February 2003	2003Feb_34	6.6	1.8	12.7	11.1	22.1	45.7	3
K2, 25 February 2003	2003Feb_35	0.5	0.3	0.0	6.1	26.5	66.6	3
K2, 25 February 2003	2003Feb_36	14.4	2.3	27.0	12.0	14.4	29.8	2
K2, 25 February 2003	2003Feb_37	7.9	1.4	18.3	9.5	18.7	44.2	3
K2, 25 February 2003	2003Feb_38	14.9	2.2	35.8	13.9	12.7	20.5	1
K2, 25 February 2003	2003Feb_39	11.8	1.5	27.7	11.2	13.4	34.5	2
K2, 25 February 2003	2003Feb_40	0.9	1.1	0.0	17.0	38.5	42.5	3
K2, 25 February 2003	2003Feb_41	0.5	0.1	0.0	4.4	23.8	71.2	3
K2, 25 February 2003	2003Feb_42	1.4	1.5	0.0	15.5	37.4	44.1	3
K2, 25 February 2003	2003Feb_43	10.0	2.0	15.8	7.6	24.0	40.7	2
K2, 25 February 2003	2003Feb_44	1.1	1.1	0.0	11.1	31.6	55.1	3
K2, 25 February 2003	2003Feb_45	5.1	1.8	18.4	5.6	20.3	48.9	3
K2, 25 February 2003	2003Feb_46	8.4	2.0	21.5	8.3	20.1	39.8	2
K2, 25 February 2003	2003Feb_47	13.8	7.3	11.0	5.0	33.0	29.9	2
K2, 25 February 2003	2003Feb_48	21.7	3.3	41.1	12.0	10.9	11.0	1
K2, 25 February 2003	2003Feb_49	5.8	1.1	16.7	8.3	14.3	53.9	3
K2, 25 February 2003	2003Feb_50	22.0	3.0	28.0	16.4	9.9	20.6	2
K2, 25 February 2003	2003Feb_51	6.3	2.6	11.7	11.4	20.6	47.4	3
K2, 25 February 2003	2003Feb_52	12.5	2.1	19.7	19.5	15.3	30.8	2
K2, 25 February 2003	2003Feb_53	0.0	2.1	3.0	16.8	22.0	56.2	3
K2, 25 February 2003	2003Feb_54	22.4	2.3	41.9	16.5	6.4	10.5	1
K2, 25 February 2003	2003Feb_55	13.0	2.5	22.6	21.0	15.0	25.9	2
K2, 25 February 2003	2003Feb_56	0.0	0.7	0.5	3.5	19.2	76.1	3
K2, 25 February 2003	2003Feb_57	0.0	0.0	0.0	2.2	13.1	84.7	3
K2, 25 February 2003	2003Feb_58	8.2	1.8	11.3	12.3	19.8	46.6	3
K2, 25 February 2003	2003Feb_59	14.5	2.6	30.0	18.3	16.3	18.3	2
K2, 25 February 2003	2003Feb_60	3.4	1.1	7.4	7.3	18.3	62.5	3
K2, 25 February 2003	2003Feb_61	29.9	2.1	32.3	20.8	5.9	8.9	1
K2, 25 February 2003	2003Feb_62	24.5	2.6	38.6	19.7	5.6	8.9	1
K2, 25 February 2003	2003Feb_63	21.1	2.5	39.8	17.3	7.9	11.4	1
K2, 25 February 2003	2003Feb_64	0.0	0.0	0.0	1.8	23.9	74.3	3
K2, 25 February 2003	2003Feb_65	18.9	2.4	34.5	13.8	8.0	22.4	1
K2, 25 February 2003	2003Feb_66	5.8	2.4	9.2	17.4	20.9	44.4	3
K2, 25 February 2003	2003Feb_67	24.9	1.6	42.9	19.8	4.4	6.4	1
K2, 25 February 2003	2003Feb_68	0.0	0.2	0.0	4.0	30.8	65.0	3
K2, 25 February 2003	2003Feb_69	0.0	0.0	0.0	1.0	12.7	86.3	3
K2, 25 February 2003	2003Feb_70	0.0	0.8	0.0	3.7	19.6	75.8	3
K2, 25 February 2003	2003Feb_71	14.3	2.9	26.0	16.1	15.9	24.8	2
K2, 25 February 2003	2003Feb_72	18.8	2.9	20.5	16.8	18.3	22.7	2
K2, 25 February 2003	2003Feb_73	22.2	2.3	42.5	16.8	5.8	10.3	1
K2, 25 February 2003	2003Feb_74	0.5	0.1	0.0	3.9	21.1	74.4	3
K2, 25 February 2003	2003Feb_75	0.4	1.1	0.0	8.4	26.3	63.8	3
K2, 25 February 2003	2003Feb_76	1.7	0.0	0.0	0.0	20.8	77.5	3
K2, 25 February 2003	2003Feb_77	7.3	2.3	17.9	9.8	18.8	43.9	3
K2, 25 February 2003	2003Feb_78	20.8	3.5	43.8	13.1	6.2	12.6	1
K2, 25 February 2003	2003Feb_79	16.6	2.2	32.4	13.4	11.4	24.1	2
K2, 25 February 2003	2003Feb_80	2.5	0.7	0.0	8.1	22.9	65.8	3

K2, 25 February 2003	2003Feb_81	1.7	1.4	1.9	8.8	22.4	63.9	3
K2, 25 February 2003	2003Feb_82	7.3	2.3	17.9	9.8	18.8	43.9	3
K2, 25 February 2003	2003Feb_83	2.2	0.7	0.0	8.3	25.2	63.5	3
K2, 25 February 2003	2003Feb_84	16.5	2.6	29.3	12.2	11.5	27.8	2
K2, 25 February 2003	2003Feb_85	0.7	0.2	0.0	5.5	24.0	69.6	3
K2, 25 February 2003	2003Feb_86	0.5	0.1	0.0	5.7	23.6	70.0	3
K2, 25 February 2003	2003Feb_87	19.0	2.5	34.3	15.0	10.4	18.9	1
K2, 25 February 2003	2003Feb_88	3.0	0.6	0.0	7.5	21.9	66.9	3
K2, 25 February 2003	2003Feb_89	6.9	1.4	8.0	7.6	20.3	55.8	3
K2, 25 February 2003	2003Feb_90	0.3	0.1	0.0	4.7	21.7	73.3	3
K2, 25 February 2003	2003Feb_91	0.9	0.3	0.0	5.1	21.6	72.0	3
K2, 25 February 2003	2003Feb_92	0.2	0.0	0.0	2.4	9.0	88.4	3
K2, 25 February 2003	2003Feb_93	12.0	2.4	25.5	11.5	14.6	33.9	2
K2, 25 February 2003	2003Feb_94	0.2	0.0	0.0	2.9	19.2	77.6	3
K2, 25 February 2003	2003Feb_95	24.6	3.2	32.1	17.0	8.5	14.6	1
K2, 25 February 2003	2003Feb_96	0.6	0.0	0.0	4.1	20.8	74.4	3
K2, 25 February 2003	2003Feb_97	0.3	0.3	0.0	9.5	36.5	53.4	3
K2, 25 February 2003	2003Feb_98	0.2	0.2	0.0	3.8	21.3	74.6	3
K2, 25 February 2003	2003Feb_99	0.2	0.0	0.0	4.0	21.8	73.9	3
K2, 25 February 2003	2003Feb_100	0.8	0.5	0.0	10.2	36.7	51.8	3
K2, 25 February 2003	2003Feb_101	26.0	2.9	40.5	14.6	5.8	10.1	1
K2, 25 February 2003	2003Feb_102	0.2	0.5	0.0	10.7	40.3	48.3	3
K2, 25 February 2003	2003Feb_103	17.7	2.7	36.7	14.6	10.9	17.4	1
K2, 25 February 2003	2003Feb_104	1.4	0.2	0.0	4.9	20.2	73.3	3
K2, 25 February 2003	2003Feb_105	1.6	1.2	1.1	7.4	23.9	64.8	3
K2, 25 February 2003	2003Feb_106	4.5	0.0	0.0	3.3	22.7	69.5	3
K2, 25 February 2003	2003Feb_107	2.4	0.4	0.0	7.5	23.8	66.0	3
K2, 25 February 2003	2003Feb_108	0.4	0.8	0.0	11.6	40.2	47.1	3
K2, 25 February 2003	2003Feb_109	0.1	0.0	0.0	3.7	20.8	75.4	3
K2, 25 February 2003	2003Feb_110	1.6	2.7	1.9	17.8	31.4	44.5	3
K2, 25 February 2003	2003Feb_111	1.4	0.5	0.0	5.9	23.1	69.1	3
K2, 25 February 2003	2003Feb_112	1.0	0.4	0.0	5.2	23.1	70.3	3
K2, 25 February 2003	2003Feb_113	0.9	0.2	0.0	4.6	21.7	72.6	3
K2, 25 February 2003	2003Feb_114	5.4	1.2	8.3	7.0	18.2	59.9	3
K2, 25 February 2003	2003Feb_115	0.2	0.0	0.0	4.4	21.5	73.9	3
K2, 25 February 2003	2003Feb_116	0.2	0.0	0.0	3.8	21.0	75.0	3
K2, 25 February 2003	2003Feb_117	3.9	2.2	2.3	11.8	31.3	48.6	3
K2, 25 February 2003	2003Feb_118	1.4	0.1	0.0	4.3	22.4	71.7	3
K2, 25 February 2003	2003Feb_119	0.7	0.0	0.0	3.7	23.5	72.1	3
K2, 25 February 2003	2003Feb_120	1.8	2.0	0.2	7.8	28.4	59.8	3
K2, 25 February 2003	2003Feb_121	0.1	0.4	0.0	4.7	22.0	72.9	3
K2, 11 July 2003	2003July_1	35.0	1.1	6.7	10.2	22.7	24.2	2
K2, 11 July 2003	2003July_2	21.3	3.1	42.1	11.9	8.8	12.9	1
K2, 11 July 2003	2003July_3	0.0	0.0	0.0	3.6	22.3	74.1	3
K2, 11 July 2003	2003July_4	17.5	3.1	36.4	13.3	12.2	17.5	1
K2, 11 July 2003	2003July_5	22.4	3.0	39.8	12.6	9.4	12.8	1
K2, 11 July 2003	2003July_6	0.1	0.9	4.0	2.3	22.9	69.8	3
K2, 11 July 2003	2003July_7	23.7	2.5	38.9	16.6	6.7	11.6	1
K2, 11 July 2003	2003July_8	24.8	3.2	43.8	13.7	5.8	8.7	1
K2, 11 July 2003	2003July_9	21.9	2.9	30.9	14.7	12.1	17.5	1
K2, 11 July 2003	2003July_10	23.0	3.1	47.0	13.1	6.2	7.7	1
K2, 11 July 2003	2003July_11	3.5	1.0	11.9	5.0	19.7	58.8	3
K2, 11 July 2003	2003July_12	23.5	3.0	46.9	15.3	4.3	7.0	1
K2, 11 July 2003	2003July_13	20.3	3.3	40.2	13.0	8.9	14.3	1
K2, 11 July 2003	2003July_14	18.3	3.2	34.3	13.1	12.0	19.1	1
K2, 11 July 2003	2003July_15	10.1	2.9	22.4	11.0	18.1	35.5	2
K2, 11 July 2003	2003July_16	24.4	3.4	42.4	10.9	8.7	10.3	1
K2, 11 July 2003	2003July_17	12.7	2.9	19.8	9.0	17.8	37.8	2
K2, 11 July 2003	2003July_18	21.9	2.8	42.6	14.4	6.6	11.6	1
K2, 11 July 2003	2003July_19	20.6	3.2	33.9	12.0	9.2	21.0	1
K2, 11 July 2003	2003July_20	41.1	4.1	23.7	11.9	7.4	11.7	1
K2, 11 July 2003	2003July_21	21.2	3.0	37.5	12.9	8.1	17.3	1
K2, 11 July 2003	2003July_22	24.1	2.8	42.5	16.5	6.5	7.5	1
K2, 11 July 2003	2003July_23	22.0	3.5	40.2	15.4	7.7	11.3	1
K2, 11 July 2003	2003July_24	20.7	4.7	30.8	10.1	12.1	21.5	2
K2, 11 July 2003	2003July_25	15.4	2.4	33.7	10.8	11.9	25.7	2
K2, 11 July 2003	2003July_26	21.1	3.3	39.0	11.5	9.1	16.0	1
K2, 11 July 2003	2003July_27	3.4	1.4	12.2	4.2	15.9	62.9	3
K2, 11 July 2003	2003July_28	0.6	0.3	3.1	0.2	5.5	90.3	3
K2, 11 July 2003	2003July_29	23.4	2.5	41.7	12.9	7.7	11.8	1
K2, 11 July 2003	2003July_30	0.0	0.2	0.0	10.3	44.1	45.5	3
K2, 11 July 2003	2003July_31	17.6	3.5	12.1	10.6	20.3	35.9	2
K2, 11 July 2003	2003July_32	12.1	2.2	27.6	10.5	18.1	29.4	2
K2, 11 July 2003	2003July_33	24.2	2.7	38.3	16.3	8.7	9.8	1
K2, 11 July 2003	2003July_34	20.2	2.0	45.7	16.7	5.4	10.1	1
K2, 11 July 2003	2003July_35	14.8	2.4	32.4	9.6	14.0	26.8	2
K2, 11 July 2003	2003July_36	9.4	3.6	23.2	9.6	17.5	36.8	2
K2, 11 July 2003	2003July_37	9.2	1.7	25.1	7.7	14.2	42.1	2
K2, 11 July 2003	2003July_38	22.0	3.2	45.0	13.9	6.3	9.6	1
K2, 11 July 2003	2003July_39	19.2	2.8	37.6	12.1	8.8	19.5	1
K2, 11 July 2003	2003July_40	22.5	3.1	47.1	12.1	5.6	9.5	1
K2, 11 July 2003	2003July_41	12.8	3.5	28.9	11.1	14.8	28.9	2
K2, 11 July 2003	2003July_42	23.6	3.0	43.7	15.6	5.2	8.8	1
K2, 11 July 2003	2003July_43	4.0	1.4	12.6	4.3	20.9	56.9	3
K2, 11 July 2003	2003July_44	26.3	3.0	42.5	11.8	7.3	9.1	1
K2, 11 July 2003	2003July_45	14.0	2.3	30.6	8.6	13.0	31.5	2

K2, 11 July 2003	2003July_46	15.7	3.2	27.3	19.1	14.9	19.9	2
K2, 11 July 2003	2003July_47	6.2	2.1	7.4	9.1	24.2	50.9	3
K2, 11 July 2003	2003July_48	0.4	0.6	0.0	6.3	25.0	67.8	3
K2, 11 July 2003	2003July_49	0.0	0.4	0.0	8.5	35.4	55.6	3
K2, 11 July 2003	2003July_50	18.2	3.0	34.3	12.8	12.2	19.5	1
K2, 11 July 2003	2003July_51	0.0	0.3	0.0	5.8	24.1	69.8	3
K2, 11 July 2003	2003July_52	18.2	3.0	33.1	12.1	10.9	22.7	2
K2, 11 July 2003	2003July_53	27.9	3.3	26.2	14.5	9.9	18.1	2
K2, 11 July 2003	2003July_54	13.3	2.1	20.6	10.2	16.9	37.0	2
K2, 11 July 2003	2003July_55	1.2	1.7	1.0	12.8	33.2	50.1	3
K2, 11 July 2003	2003July_56	5.3	1.5	13.9	5.0	19.7	54.6	3
K2, 11 July 2003	2003July_57	0.0	0.1	0.0	4.2	23.3	72.4	3
K2, 11 July 2003	2003July_58	29.5	2.6	41.7	15.0	4.2	7.0	1
K2, 11 July 2003	2003July_59	0.4	0.7	0.0	5.6	24.9	68.4	3
K2, 11 July 2003	2003July_60	0.3	0.4	0.0	6.6	27.0	65.8	3
K2, 11 July 2003	2003July_61	0.0	0.2	0.0	4.7	23.8	71.3	3
K2, 11 July 2003	2003July_62	25.5	3.1	33.9	16.9	9.1	11.4	1
K2, 11 July 2003	2003July_63	19.5	3.2	33.1	11.9	10.6	21.8	2
K2, 11 July 2003	2003July_64	23.6	3.0	28.5	15.1	13.0	16.8	1
K2, 11 July 2003	2003July_65	20.1	4.1	33.9	12.8	12.1	17.1	1
K2, 11 July 2003	2003July_66	10.7	2.5	16.4	15.0	22.8	32.6	2
K2, 11 July 2003	2003July_67	23.9	2.3	32.3	14.5	10.3	16.7	1
K2, 11 July 2003	2003July_68	22.3	3.3	35.0	12.3	10.6	16.6	1
K2, 11 July 2003	2003July_69	26.5	1.9	28.2	15.6	11.0	16.8	1
K2, 11 July 2003	2003July_70	23.1	3.6	37.7	12.5	9.3	13.8	1
K2, 11 July 2003	2003July_71	20.8	3.1	26.3	12.9	14.0	23.0	2
K2, 11 July 2003	2003July_72	27.5	3.6	29.0	19.9	9.2	10.9	1
K2, 11 July 2003	2003July_73	2.3	1.1	7.8	3.9	22.9	62.1	3
K2, 11 July 2003	2003July_74	0.0	0.4	0.0	4.8	24.0	70.8	3
K2, 11 July 2003	2003July_75	12.7	2.1	20.4	10.2	12.6	41.9	2
K2, 11 July 2003	2003July_76	22.7	3.2	42.0	11.8	8.4	11.9	1
K2, 11 July 2003	2003July_77	0.0	0.1	0.0	6.5	34.1	59.4	3
K2, 11 July 2003	2003July_78	19.2	4.4	21.9	13.5	14.1	26.9	2
K2, 11 July 2003	2003July_79	21.1	2.6	33.5	13.2	10.3	19.2	1
K2, 11 July 2003	2003July_80	0.8	0.8	3.1	4.3	23.0	67.9	3
K2, 11 July 2003	2003July_81	18.1	2.5	21.4	13.6	16.4	28.0	2
K2, 11 July 2003	2003July_82	0.0	0.2	0.0	7.7	34.7	57.4	3
K2, 11 July 2003	2003July_83	9.4	2.2	15.5	10.0	20.5	42.4	2
K2, 11 July 2003	2003July_84	23.7	3.0	44.4	14.1	6.2	8.6	1
K2, 11 July 2003	2003July_85	12.7	2.2	27.8	9.3	15.0	33.0	2
K2, 11 July 2003	2003July_86	16.9	2.5	26.4	13.0	14.8	26.4	2
K2, 11 July 2003	2003July_87	19.4	3.3	40.4	12.3	9.3	15.2	1
K2, 11 July 2003	2003July_88	17.4	2.9	39.0	12.4	12.5	15.7	1
K2, 4 October 2003	2003Oct_1	33.4	0.4	18.4	17.7	11.3	18.8	2
K2, 4 October 2003	2003Oct_2	21.6	0.9	25.9	12.3	14.0	25.3	2
K2, 4 October 2003	2003Oct_3	16.3	2.5	21.2	13.2	17.7	29.2	2
K2, 4 October 2003	2003Oct_4	33.6	1.4	30.2	12.7	9.2	12.9	1
K2, 4 October 2003	2003Oct_5	27.6	2.6	38.3	10.9	9.9	10.8	1
K2, 4 October 2003	2003Oct_6	4.5	1.2	13.1	4.7	19.5	57.0	3
K2, 4 October 2003	2003Oct_7	5.0	1.3	14.0	5.6	20.2	53.9	3
K2, 4 October 2003	2003Oct_8	3.3	0.0	0.0	10.1	43.3	43.4	3
K2, 4 October 2003	2003Oct_9	14.1	2.3	30.0	8.8	14.1	30.7	2
K2, 4 October 2003	2003Oct_10	22.5	3.8	45.6	14.0	5.8	8.3	1
K2, 4 October 2003	2003Oct_11	19.4	3.2	38.3	12.9	9.0	17.2	1
K2, 4 October 2003	2003Oct_12	21.7	3.2	46.8	10.5	6.1	11.6	1
K2, 4 October 2003	2003Oct_13	1.9	1.0	7.3	3.4	21.1	65.3	3
K2, 4 October 2003	2003Oct_14	2.4	1.1	8.3	4.2	21.4	62.6	3
K2, 4 October 2003	2003Oct_15	20.0	2.8	40.2	12.8	9.7	14.6	1
K2, 4 October 2003	2003Oct_16	17.6	2.9	37.4	11.7	10.1	20.3	1
K2, 4 October 2003	2003Oct_17	0.0	0.1	0.0	4.0	23.3	72.6	3
K2, 4 October 2003	2003Oct_18	19.1	2.5	30.3	11.3	9.0	27.7	2
K2, 4 October 2003	2003Oct_19	8.0	1.8	19.6	6.0	18.0	46.6	3
K2, 4 October 2003	2003Oct_20	23.2	2.5	38.8	16.0	7.2	12.2	1
K2, 4 October 2003	2003Oct_21	23.2	3.2	38.2	11.9	8.8	14.7	1
K2, 4 October 2003	2003Oct_22	23.1	3.4	42.6	12.5	7.3	11.2	1
K2, 4 October 2003	2003Oct_23	21.1	2.9	41.6	14.5	8.0	11.9	1
K2, 4 October 2003	2003Oct_24	18.9	3.2	20.8	14.5	15.9	26.7	2
K2, 4 October 2003	2003Oct_25	17.8	2.3	34.4	11.1	11.3	23.0	2
K2, 4 October 2003	2003Oct_26	20.3	3.4	39.6	13.5	8.9	14.3	1
K2, 4 October 2003	2003Oct_27	0.0	0.0	0.0	3.3	20.7	76.0	3
K2, 4 October 2003	2003Oct_28	0.0	0.2	0.0	5.7	25.7	68.5	3
K2, 4 October 2003	2003Oct_29	0.0	0.1	0.0	3.7	22.0	74.3	3
K2, 4 October 2003	2003Oct_30	0.0	0.0	0.0	3.2	20.5	76.4	3
K2, 4 October 2003	2003Oct_31	0.0	0.4	0.0	3.6	20.9	75.2	3
K2, 4 October 2003	2003Oct_32	0.0	1.1	0.0	4.2	22.0	72.7	3
K2, 4 October 2003	2003Oct_33	26.8	2.9	35.5	16.8	7.7	10.4	1
K2, 4 October 2003	2003Oct_34	0.0	0.1	0.0	4.8	23.5	71.6	3
K2, 4 October 2003	2003Oct_35	0.0	0.0	0.0	4.8	23.3	71.9	3
K2, 4 October 2003	2003Oct_36	23.4	3.0	44.2	12.8	6.8	9.8	1
K2, 4 October 2003	2003Oct_37	0.0	0.4	0.0	5.3	24.5	69.7	3
K2, 4 October 2003	2003Oct_38	22.5	2.5	35.3	14.1	9.9	15.8	1
K2, 4 October 2003	2003Oct_39	1.5	0.5	1.0	10.2	39.9	46.9	3
K2, 4 October 2003	2003Oct_40	0.0	0.1	0.0	4.3	23.7	71.9	3
K2, 4 October 2003	2003Oct_41	1.7	0.8	7.0	3.1	20.9	66.6	3
K2, 4 October 2003	2003Oct_42	18.2	3.3	37.1	13.9	11.0	16.5	1
K2, 4 October 2003	2003Oct_43	0.0	0.0	0.0	2.6	19.9	77.5	3

K2, 4 October 2003	2003Oct_44	0.0	0.0	0.0	3.5	21.8	74.7	3
K2, 4 October 2003	2003Oct_45	0.0	0.3	0.0	3.9	22.9	72.9	3
K2, 4 October 2003	2003Oct_46	0.0	0.0	0.0	2.9	20.2	76.9	3
K2, 4 October 2003	2003Oct_47	0.0	0.1	0.0	4.6	24.2	71.1	3
K2, 4 October 2003	2003Oct_48	1.1	1.4	0.6	8.2	24.5	64.2	3
K2, 4 October 2003	2003Oct_49	0.0	0.9	0.0	5.4	23.4	70.3	3
K2, 4 October 2003	2003Oct_50	0.0	0.0	0.0	3.6	20.7	75.7	3
K2, 4 October 2003	2003Oct_51	23.0	3.3	42.5	11.6	8.1	11.5	1
K2, 4 October 2003	2003Oct_52	22.7	3.0	36.4	13.4	10.3	14.2	1
K2, 4 October 2003	2003Oct_53	0.0	0.6	0.0	5.7	24.4	69.4	3
K2, 4 October 2003	2003Oct_54	18.3	2.9	42.6	13.5	10.0	12.7	1
K2, 4 October 2003	2003Oct_55	14.0	2.6	23.6	11.4	18.1	30.3	2
K2, 4 October 2003	2003Oct_56	0.0	0.6	3.8	2.1	23.7	69.9	3
K2, 4 October 2003	2003Oct_57	30.4	1.8	18.0	11.9	14.0	23.9	2
K2, 4 October 2003	2003Oct_58	17.6	3.1	38.7	12.7	10.9	17.1	1
K2, 4 October 2003	2003Oct_59	21.3	3.0	28.8	13.8	10.3	22.8	2
K2, 4 October 2003	2003Oct_60	6.6	2.5	9.6	10.3	25.5	45.5	3
K2, 4 October 2003	2003Oct_61	24.7	3.3	46.0	12.9	5.4	7.8	1
K2, 4 October 2003	2003Oct_62	22.0	2.7	31.1	12.6	11.8	19.8	2
K2, 4 October 2003	2003Oct_63	0.0	0.2	0.0	4.8	24.4	70.5	3
K2, 4 October 2003	2003Oct_64	12.3	3.1	15.6	10.0	22.1	36.9	2
K2, 4 October 2003	2003Oct_65	17.0	2.6	36.9	11.8	10.9	20.9	1
K2, 4 October 2003	2003Oct_66	19.8	2.2	33.5	13.1	13.4	18.1	1
K2, 4 October 2003	2003Oct_67	24.6	3.2	42.9	12.0	7.1	10.2	1
K2, 4 October 2003	2003Oct_68	17.3	1.8	24.2	9.7	15.8	31.2	2
K2, 4 October 2003	2003Oct_69	24.9	3.1	38.4	11.0	9.1	13.6	1
K2, 4 October 2003	2003Oct_70	24.1	3.2	39.3	12.9	8.2	12.3	1
K2, 4 October 2003	2003Oct_71	0.0	0.1	0.0	4.9	24.6	70.4	3
K2, 4 October 2003	2003Oct_72	0.2	0.2	0.0	4.2	23.8	71.6	3
K2, 4 October 2003	2003Oct_73	9.3	1.4	20.5	6.1	17.0	45.6	3
K2, 4 October 2003	2003Oct_74	18.6	2.9	31.8	12.4	11.3	23.1	2
K2, 4 October 2003	2003Oct_75	18.8	3.4	43.7	11.5	7.2	15.4	1
K2, 4 October 2003	2003Oct_76	0.0	0.2	0.0	3.6	22.7	73.5	3
K2, 4 October 2003	2003Oct_77	13.9	2.3	31.1	9.3	13.0	30.4	2
K2, 4 October 2003	2003Oct_78	13.6	2.5	24.9	11.0	17.9	30.1	2
K2, 4 October 2003	2003Oct_79	1.1	0.8	4.2	2.7	21.6	69.5	3
K2, 4 October 2003	2003Oct_80	0.0	0.0	0.0	3.5	22.4	74.1	3
K2, 4 October 2003	2003Oct_81	0.6	0.5	0.0	5.4	24.5	69.0	3
K2, 4 October 2003	2003Oct_82	16.1	3.2	17.8	13.0	19.4	30.5	2
K2, 4 October 2003	2003Oct_83	0.0	0.2	0.0	5.6	24.8	69.4	3
K2, 4 October 2003	2003Oct_84	6.2	2.1	7.4	9.1	24.2	50.9	3
K2, 4 October 2003	2003Oct_85	0.4	0.6	0.0	6.3	25.0	67.8	3
K2, 4 October 2003	2003Oct_86	0.0	0.4	0.0	8.5	35.4	55.6	3
K2, 4 October 2003	2003Oct_87	18.2	3.0	34.3	12.8	12.2	19.5	1
K2, 4 October 2003	2003Oct_88	0.0	0.3	0.0	5.8	24.1	69.8	3
K2, 4 October 2003	2003Oct_89	18.2	3.0	33.1	12.1	10.9	22.7	2
K2, 4 October 2003	2003Oct_90	27.9	3.3	26.2	14.5	9.9	18.1	2
K2, 4 October 2003	2003Oct_91	13.3	2.1	20.6	10.2	16.9	37.0	2
K2, 4 October 2003	2003Oct_92	1.2	1.7	1.0	12.8	33.2	50.1	3
K2, 4 October 2003	2003Oct_93	5.3	1.5	13.9	5.0	19.7	54.6	3
K2, 4 October 2003	2003Oct_94	0.0	0.1	0.0	4.2	23.3	72.4	3
K2, 4 October 2003	2003Oct_95	29.5	2.6	41.7	15.0	4.2	7.0	1
K2, 4 October 2003	2003Oct_96	0.4	0.7	0.0	5.6	24.9	68.4	3
K2, 4 October 2003	2003Oct_97	0.3	0.4	0.0	6.6	27.0	65.8	3
K2, 4 October 2003	2003Oct_98	0.0	0.2	0.0	4.7	23.8	71.3	3
K2, 4 October 2003	2003Oct_99	25.5	3.1	33.9	16.9	9.1	11.4	1
K2, 4 October 2003	2003Oct_100	19.5	3.2	33.1	11.9	10.6	21.8	2
K2, 4 October 2003	2003Oct_101	23.6	3.0	28.5	15.1	13.0	16.8	1
K2, 4 October 2003	2003Oct_102	20.1	4.1	33.9	12.8	12.1	17.1	1
K2, 4 October 2003	2003Oct_103	10.7	2.5	16.4	15.0	22.8	32.6	2
K2, 4 October 2003	2003Oct_104	23.9	2.3	32.3	14.5	10.3	16.7	1
K2, 4 October 2003	2003Oct_105	22.3	3.3	35.0	12.3	10.6	16.6	1
K2, 4 October 2003	2003Oct_106	26.5	1.9	28.2	15.6	11.0	16.8	1
K2, 4 October 2003	2003Oct_107	23.1	3.6	37.7	12.5	9.3	13.8	1
K2, 4 October 2003	2003Oct_108	20.8	3.1	26.3	12.9	14.0	23.0	2
K2, 4 October 2003	2003Oct_109	27.5	3.6	29.0	19.9	9.2	10.9	1
K2, 4 October 2003	2003Oct_110	2.3	1.1	7.8	3.9	22.9	62.1	3
K2, 4 October 2003	2003Oct_111	0.0	0.4	0.0	4.8	24.0	70.8	3
K2, 4 October 2003	2003Oct_112	12.7	2.1	20.4	10.2	12.6	41.9	2
K2, 4 October 2003	2003Oct_113	22.7	3.2	42.0	11.8	8.4	11.9	1
K2, 4 October 2003	2003Oct_114	0.0	0.1	0.0	6.5	34.1	59.4	3
K2, 4 October 2003	2003Oct_115	19.2	4.4	21.9	13.5	14.1	26.9	2
K2, 4 October 2003	2003Oct_116	21.1	2.6	33.5	13.2	10.3	19.2	1
K2, 4 October 2003	2003Oct_117	0.8	0.8	3.1	4.3	23.0	67.9	3
K2, 4 October 2003	2003Oct_118	18.1	2.5	21.4	13.6	16.4	28.0	2
K2, 4 October 2003	2003Oct_119	0.0	0.2	0.0	7.7	34.7	57.4	3
K2, 4 October 2003	2003Oct_120	9.4	2.2	15.5	10.0	20.5	42.4	2
K2, 4 October 2003	2003Oct_121	23.7	3.0	44.4	14.1	6.2	8.6	1
K2, 4 October 2003	2003Oct_122	12.7	2.2	27.8	9.3	15.0	33.0	2
K2, 4 October 2003	2003Oct_123	16.9	2.5	26.4	13.0	14.8	26.4	2
K2, 4 October 2003	2003Oct_124	19.4	3.3	40.4	12.3	9.3	15.2	1
K2, 4 October 2003	2003Oct_125	17.4	2.9	39.0	12.4	12.5	15.7	1
K2, 4 October 2003	2003Oct_126	35.0	1.1	6.7	10.2	22.7	24.2	2
K2, 4 October 2003	2003Oct_127	21.3	3.1	42.1	11.9	8.8	12.9	1
K2, 4 October 2003	2003Oct_128	0.0	0.0	0.0	3.6	22.3	74.1	3
K2, 4 October 2003	2003Oct_129	17.5	3.1	36.4	13.3	12.2	17.5	1

K2, 4 October 2003	2003Oct_130	22.4	3.0	39.8	12.6	9.4	12.8	1
K2, 4 October 2003	2003Oct_131	0.1	0.9	4.0	2.3	22.9	69.8	3
K2, 4 October 2003	2003Oct_132	23.7	2.5	38.9	16.6	6.7	11.6	1
K2, 4 October 2003	2003Oct_133	24.8	3.2	43.8	13.7	5.8	8.7	1
K2, 4 October 2003	2003Oct_134	21.9	2.9	30.9	14.7	12.1	17.5	1
K2, 4 October 2003	2003Oct_135	23.0	3.1	47.0	13.1	6.2	7.7	1
K2, 4 October 2003	2003Oct_136	3.5	1.0	11.9	5.0	19.7	58.8	3
K2, 4 October 2003	2003Oct_137	23.5	3.0	46.9	15.3	4.3	7.0	1
K2, 4 October 2003	2003Oct_138	20.3	3.3	40.2	13.0	8.9	14.3	1
K2, 4 October 2003	2003Oct_139	18.3	3.2	34.3	13.1	12.0	19.1	1
K2, 4 October 2003	2003Oct_140	10.1	2.9	22.4	11.0	18.1	35.5	2
K2, 4 October 2003	2003Oct_141	24.4	3.4	42.4	10.9	8.7	10.3	1
K2, 4 October 2003	2003Oct_142	12.7	2.9	19.8	9.0	17.8	37.8	2
K2, 4 October 2003	2003Oct_143	21.9	2.8	42.6	14.4	6.6	11.6	1
K2, 4 October 2003	2003Oct_144	20.6	3.2	33.9	12.0	9.2	21.0	1
K2, 4 October 2003	2003Oct_145	41.1	4.1	23.7	11.9	7.4	11.7	1
K2, 4 October 2003	2003Oct_146	21.2	3.0	37.5	12.9	8.1	17.3	1
K2, 4 October 2003	2003Oct_147	24.1	2.8	42.5	16.5	6.5	7.5	1
K2, 4 October 2003	2003Oct_148	22.0	3.5	40.2	15.4	7.7	11.3	1
K2, 4 October 2003	2003Oct_149	20.7	4.7	30.8	10.1	12.1	21.5	2
K2, 4 October 2003	2003Oct_150	15.4	2.4	33.7	10.8	11.9	25.7	2
K2, 4 October 2003	2003Oct_151	21.1	3.3	39.0	11.5	9.1	16.0	1
K2, 4 October 2003	2003Oct_152	3.4	1.4	12.2	4.2	15.9	62.9	3
K2, 4 October 2003	2003Oct_153	0.6	0.3	3.1	0.2	5.5	90.3	3
K2, 4 October 2003	2003Oct_154	23.4	2.5	41.7	12.9	7.7	11.8	1
K2, 4 October 2003	2003Oct_155	0.0	0.2	0.0	10.3	44.1	45.5	3
K2, 4 October 2003	2003Oct_156	17.6	3.5	12.1	10.6	20.3	35.9	2
K2, 4 October 2003	2003Oct_157	12.1	2.2	27.6	10.5	18.1	29.4	2
K2, 4 October 2003	2003Oct_158	24.2	2.7	38.3	16.3	8.7	9.8	1
K2, 4 October 2003	2003Oct_159	20.2	2.0	45.7	16.7	5.4	10.1	1
K2, 4 October 2003	2003Oct_160	14.8	2.4	32.4	9.6	14.0	26.8	2
K2, 4 October 2003	2003Oct_161	9.4	3.6	23.2	9.6	17.5	36.8	2
K2, 4 October 2003	2003Oct_162	9.2	1.7	25.1	7.7	14.2	42.1	2
K2, 4 October 2003	2003Oct_163	22.0	3.2	45.0	13.9	6.3	9.6	1
K2, 4 October 2003	2003Oct_164	19.2	2.8	37.6	12.1	8.8	19.5	1
K2, 4 October 2003	2003Oct_165	22.5	3.1	47.1	12.1	5.6	9.5	1
K2, 4 October 2003	2003Oct_166	12.8	3.5	28.9	11.1	14.8	28.9	2
K2, 4 October 2003	2003Oct_167	23.6	3.0	43.7	15.6	5.2	8.8	1
K2, 4 October 2003	2003Oct_168	4.0	1.4	12.6	4.3	20.9	56.9	3
K2, 4 October 2003	2003Oct_169	26.3	3.0	42.5	11.8	7.3	9.1	1
K2, 4 October 2003	2003Oct_170	14.0	2.3	30.6	8.6	13.0	31.5	2
K2, 4 October 2003	2003Oct_171	15.7	3.2	27.3	19.1	14.9	19.9	2
K2, 4 October 2003	2003Oct_172	33.4	0.4	18.4	17.7	11.3	18.8	2
K2, 4 October 2003	2003Oct_173	21.6	0.9	25.9	12.3	14.0	25.3	2
K2, 4 October 2003	2003Oct_174	16.3	2.5	21.2	13.2	17.7	29.2	2
K2, 4 October 2003	2003Oct_175	33.6	1.4	30.2	12.7	9.2	12.9	1
K2, 4 October 2003	2003Oct_176	27.6	2.6	38.3	10.9	9.9	10.8	1
K2, 4 October 2003	2003Oct_177	4.5	1.2	13.1	4.7	19.5	57.0	3
K2, 4 October 2003	2003Oct_178	5.0	1.3	14.0	5.6	20.2	53.9	3
K2, 4 October 2003	2003Oct_179	3.3	0.0	0.0	10.1	43.3	43.4	3
K2, 4 October 2003	2003Oct_180	14.1	2.3	30.0	8.8	14.1	30.7	2
K2, 4 October 2003	2003Oct_181	22.5	3.8	45.6	14.0	5.8	8.3	1
K2, 4 October 2003	2003Oct_182	19.4	3.2	38.3	12.9	9.0	17.2	1
K2, 4 October 2003	2003Oct_183	21.7	3.2	46.8	10.5	6.1	11.6	1
K2, 4 October 2003	2003Oct_184	1.9	1.0	7.3	3.4	21.1	65.3	3
K2, 4 October 2003	2003Oct_185	2.4	1.1	8.3	4.2	21.4	62.6	3
K2, 4 October 2003	2003Oct_186	20.0	2.8	40.2	12.8	9.7	14.6	1
K2, 4 October 2003	2003Oct_187	17.6	2.9	37.4	11.7	10.1	20.3	1
K2, 4 October 2003	2003Oct_188	0.0	0.1	0.0	4.0	23.3	72.6	3
K2, 4 October 2003	2003Oct_189	19.1	2.5	30.3	11.3	9.0	27.7	2
K2, 4 October 2003	2003Oct_190	8.0	1.8	19.6	6.0	18.0	46.6	3
K2, 4 October 2003	2003Oct_191	23.2	2.5	38.8	16.0	7.2	12.2	1
K2, 4 October 2003	2003Oct_192	23.2	3.2	38.2	11.9	8.8	14.7	1
K2, 4 October 2003	2003Oct_193	23.1	3.4	42.6	12.5	7.3	11.2	1
K2, 4 October 2003	2003Oct_194	21.1	2.9	41.6	14.5	8.0	11.9	1
K2, 4 October 2003	2003Oct_195	18.9	3.2	20.8	14.5	15.9	26.7	2
K2, 4 October 2003	2003Oct_196	17.8	2.3	34.4	11.1	11.3	23.0	2
K2, 4 October 2003	2003Oct_197	20.3	3.4	39.6	13.5	8.9	14.3	1
K2, 4 October 2003	2003Oct_198	0.0	0.0	0.0	3.3	20.7	76.0	3
K2, 4 October 2003	2003Oct_199	0.0	0.2	0.0	5.7	25.7	68.5	3
K2, 4 October 2003	2003Oct_200	0.0	0.1	0.0	3.7	22.0	74.3	3
K2, 4 October 2003	2003Oct_201	0.0	0.0	0.0	3.2	20.5	76.4	3
K2, 4 October 2003	2003Oct_202	0.0	0.4	0.0	3.6	20.9	75.2	3
K2, 4 October 2003	2003Oct_203	0.0	1.1	0.0	4.2	22.0	72.7	3
K2, 4 October 2003	2003Oct_204	26.8	2.9	35.5	16.8	7.7	10.4	1
K2, 4 October 2003	2003Oct_205	0.0	0.1	0.0	4.8	23.5	71.6	3
K2, 4 October 2003	2003Oct_206	0.0	0.0	0.0	4.8	23.3	71.9	3
K2, 4 October 2003	2003Oct_207	23.4	3.0	44.2	12.8	6.8	9.8	1
K2, 4 October 2003	2003Oct_208	0.0	0.4	0.0	5.3	24.5	69.7	3
K2, 4 October 2003	2003Oct_209	22.5	2.5	35.3	14.1	9.9	15.8	1
K2, 4 October 2003	2003Oct_210	1.5	0.5	1.0	10.2	39.9	46.9	3
K2, 4 October 2003	2003Oct_211	0.0	0.1	0.0	4.3	23.7	71.9	3
K2, 4 October 2003	2003Oct_212	1.7	0.8	7.0	3.1	20.9	66.6	3
K2, 4 October 2003	2003Oct_213	18.2	3.3	37.1	13.9	11.0	16.5	1
K2, 4 October 2003	2003Oct_214	0.0	0.0	0.0	2.6	19.9	77.5	3
K2, 4 October 2003	2003Oct_215	17.2	2.4	27.7	13.3	13.3	26.1	2

K2, 4 October 2003	2003Oct_216	3.4	2.3	8.3	9.8	27.5	48.8	3
K2, 4 October 2003	2003Oct_217	1.4	2.0	0.0	10.7	36.1	49.8	3
K2, 4 October 2003	2003Oct_218	0.0	0.8	0.0	7.4	29.0	62.8	3
K2, 4 October 2003	2003Oct_219	4.0	1.3	10.9	5.0	18.9	60.0	3
K2, 4 October 2003	2003Oct_220	20.8	3.1	44.6	12.2	8.2	11.1	1
K2, 4 October 2003	2003Oct_221	22.2	2.9	41.0	12.6	8.5	13.0	1
K2, 4 October 2003	2003Oct_222	22.4	3.1	41.5	13.9	7.8	11.2	1
K2, 4 October 2003	2003Oct_223	15.8	2.5	33.0	13.1	15.7	20.0	2
K2, 4 October 2003	2003Oct_224	20.2	3.0	37.7	16.0	8.4	14.6	1
K2, 4 October 2003	2003Oct_225	23.0	2.9	45.0	12.8	6.2	10.0	1
K2, 4 October 2003	2003Oct_226	0.0	0.5	2.0	2.8	21.5	73.2	3
K2, 4 October 2003	2003Oct_227	24.6	1.9	31.4	16.3	12.1	13.7	1
K2, 4 October 2003	2003Oct_228	19.0	2.2	33.1	12.0	11.3	22.4	2
K2, 4 October 2003	2003Oct_229	12.0	1.9	28.6	9.2	13.9	34.4	2
K2, 4 October 2003	2003Oct_230	22.2	3.3	43.2	15.7	6.8	8.8	1
K2, 4 October 2003	2003Oct_231	10.2	2.2	18.3	14.0	20.0	35.3	2
K2, 4 October 2003	2003Oct_232	15.3	2.4	26.0	13.4	13.9	28.9	2
K2, 4 October 2003	2003Oct_233	17.9	2.6	41.7	14.6	8.8	14.4	1
K2, 4 October 2003	2003Oct_234	20.4	3.6	39.8	13.0	9.5	13.8	1
K2, 4 October 2003	2003Oct_235	14.7	2.4	26.4	9.5	13.9	33.1	2
K2, 4 October 2003	2003Oct_236	27.5	4.3	25.6	13.2	11.1	18.4	2
K2, 4 October 2003	2003Oct_237	6.1	2.5	7.5	12.5	26.4	45.1	3
K2, 4 October 2003	2003Oct_238	1.5	0.8	0.0	6.8	24.0	66.9	3
K2, 4 October 2003	2003Oct_239	23.7	3.0	37.2	13.3	8.8	13.9	1
K2, 4 October 2003	2003Oct_240	2.8	1.1	7.3	4.4	21.8	62.6	3
K2, 4 October 2003	2003Oct_241	11.2	0.3	4.8	7.7	23.6	52.4	3
K2, 4 October 2003	2003Oct_242	21.6	3.1	37.8	12.3	10.0	15.3	1
K2, 4 October 2003	2003Oct_243	2.2	1.1	8.1	3.9	21.3	63.4	3
K2, 4 October 2003	2003Oct_244	18.8	2.9	42.5	12.9	9.2	13.7	1
K2, 4 October 2003	2003Oct_245	19.6	3.1	25.6	14.0	12.9	24.8	2
K2, 4 October 2003	2003Oct_246	15.1	2.0	30.0	10.9	12.9	29.0	2
K2, 4 October 2003	2003Oct_247	8.1	1.5	17.0	10.7	19.2	43.5	3
K2, 4 October 2003	2003Oct_248	18.9	2.6	45.7	14.6	6.2	12.0	1
K2, 4 October 2003	2003Oct_249	24.8	3.2	42.1	11.3	7.5	11.0	1
K2, 4 October 2003	2003Oct_250	16.6	2.6	37.0	12.5	11.7	19.6	1
K2, 4 October 2003	2003Oct_251	3.0	1.3	8.5	5.6	21.6	60.1	3
K2, 4 October 2003	2003Oct_252	18.7	2.8	36.2	13.8	13.0	15.6	1
K2, 4 October 2003	2003Oct_253	20.0	2.5	36.2	13.4	10.5	17.5	1
K2, 4 October 2003	2003Oct_254	16.5	2.9	17.0	13.6	20.2	29.8	2
K2, 4 October 2003	2003Oct_255	13.4	1.8	27.2	8.6	13.7	35.3	2
K2, 8 April 2004	2004Apr_1	14.3	2.0	29.9	11.8	14.0	28.1	2
K2, 8 April 2004	2004Apr_2	22.3	3.0	43.7	14.1	7.0	9.8	1
K2, 8 April 2004	2004Apr_3	19.7	2.8	40.8	14.9	9.7	12.0	1
K2, 8 April 2004	2004Apr_4	23.5	3.3	44.7	12.5	5.7	10.3	1
K2, 8 April 2004	2004Apr_5	21.4	3.1	27.7	13.7	12.8	21.3	2
K2, 8 April 2004	2004Apr_6	22.4	3.6	31.5	13.9	13.6	14.9	1
K2, 8 April 2004	2004Apr_7	16.8	2.5	40.5	14.7	10.6	15.0	1
K2, 8 April 2004	2004Apr_8	15.8	2.6	15.8	11.2	18.1	36.5	2
K2, 8 April 2004	2004Apr_9	13.4	2.4	17.8	12.6	19.6	34.3	2
K2, 8 April 2004	2004Apr_10	19.5	2.6	38.1	13.9	10.0	15.9	1
K2, 8 April 2004	2004Apr_11	15.3	3.4	33.0	11.5	10.8	26.0	2
K2, 8 April 2004	2004Apr_12	6.6	1.8	7.8	13.1	23.5	47.2	3
K2, 8 April 2004	2004Apr_13	0.9	0.8	3.8	3.7	21.8	69.0	3
K2, 8 April 2004	2004Apr_14	21.8	3.0	38.9	11.7	9.1	15.5	1
K2, 8 April 2004	2004Apr_15	20.9	3.2	43.9	13.4	7.3	11.3	1
K2, 8 April 2004	2004Apr_16	9.5	4.9	12.2	9.6	20.6	43.1	3
K2, 8 April 2004	2004Apr_17	9.2	1.9	23.1	7.2	15.3	43.2	2
K2, 8 April 2004	2004Apr_18	19.4	2.8	45.5	12.7	6.8	12.8	1
K2, 8 April 2004	2004Apr_19	20.3	3.1	45.4	13.1	6.8	11.4	1
K2, 8 April 2004	2004Apr_20	20.0	3.0	44.8	14.0	8.5	9.7	1
K2, 8 April 2004	2004Apr_21	24.2	3.2	43.0	13.5	6.7	9.3	1
K2, 8 April 2004	2004Apr_22	26.1	3.3	43.0	13.0	5.8	8.8	1
K2, 8 April 2004	2004Apr_23	17.9	2.9	38.9	12.3	9.7	18.2	1
K2, 8 April 2004	2004Apr_24	20.1	2.6	40.5	13.8	9.2	13.8	1
K2, 8 April 2004	2004Apr_25	22.3	2.9	42.0	12.8	8.3	11.8	1
K2, 8 April 2004	2004Apr_26	20.3	2.2	31.6	11.2	12.5	22.2	2
K2, 8 April 2004	2004Apr_27	3.7	1.0	4.7	6.7	19.5	64.4	3
K2, 8 April 2004	2004Apr_28	16.6	2.7	33.2	10.0	10.2	27.2	2
K2, 8 April 2004	2004Apr_29	0.9	0.8	3.5	3.7	22.5	68.6	3
K2, 8 April 2004	2004Apr_30	8.2	2.6	11.4	10.8	21.7	45.3	3
K2, 8 April 2004	2004Apr_31	21.4	3.1	42.4	13.2	6.9	12.9	1
K2, 8 April 2004	2004Apr_32	15.8	2.8	39.1	12.4	12.5	17.5	1
K2, 8 April 2004	2004Apr_33	19.9	2.8	43.8	13.0	8.6	11.8	1
K2, 8 April 2004	2004Apr_34	20.9	3.2	42.1	14.8	7.3	11.8	1
K2, 8 April 2004	2004Apr_35	28.1	3.5	29.6	13.4	10.3	15.1	1
K2, 8 April 2004	2004Apr_36	23.4	3.1	37.8	14.1	7.8	13.8	1
K2, 8 April 2004	2004Apr_37	21.2	2.7	43.7	14.9	6.7	10.8	1
K2, 8 April 2004	2004Apr_38	7.9	2.3	18.3	7.6	13.3	50.6	3
K2, 8 April 2004	2004Apr_39	20.8	3.0	41.5	14.1	7.5	13.2	1
K2, 8 April 2004	2004Apr_40	25.8	2.8	34.3	13.9	8.0	15.1	1
K2, 8 April 2004	2004Apr_41	22.6	2.7	36.7	15.9	7.9	14.3	1
K2, 8 April 2004	2004Apr_42	10.6	2.4	17.3	11.1	20.4	38.2	2
K2, 8 April 2004	2004Apr_43	7.8	1.6	19.7	6.7	15.8	48.3	3
K2, 8 April 2004	2004Apr_44	5.3	1.5	14.8	5.1	17.5	55.8	3
K2, 8 April 2004	2004Apr_45	17.9	3.3	20.2	11.2	15.4	32.0	2
K2, 8 April 2004	2004Apr_46	17.9	2.9	35.7	11.8	11.2	20.5	1

K2, 8 April 2004	2004Apr_47	0.4	0.9	0.1	4.8	23.1	70.7	3
K2, 8 April 2004	2004Apr_48	26.1	3.2	23.3	11.4	11.9	24.1	2
K2, 8 April 2004	2004Apr_49	18.1	3.1	37.1	14.8	14.2	12.7	1
K2, 8 April 2004	2004Apr_50	18.7	1.5	22.1	17.1	18.7	22.0	2
K2, 8 April 2004	2004Apr_51	16.0	2.9	15.3	14.4	21.6	29.9	2
K2, 8 April 2004	2004Apr_52	18.8	3.4	44.3	14.4	7.8	11.3	1
K2, 8 April 2004	2004Apr_53	3.2	1.3	10.5	4.3	20.0	60.7	3
K2, 8 April 2004	2004Apr_54	23.1	2.9	40.5	15.0	6.4	12.0	1
K2, 8 April 2004	2004Apr_55	7.4	1.1	1.4	20.0	32.9	37.2	3
K2, 8 April 2004	2004Apr_56	4.4	0.4	0.0	11.0	32.4	51.8	3
K2, 8 April 2004	2004Apr_57	2.0	0.0	0.0	6.8	29.1	62.1	3
K2, 8 April 2004	2004Apr_58	17.2	2.8	40.0	16.4	9.2	14.5	1
K2, 8 April 2004	2004Apr_59	14.1	2.9	31.9	19.2	13.3	18.6	2
K2, 8 April 2004	2004Apr_60	17.7	3.1	33.0	21.1	9.8	15.2	1
K2, 8 April 2004	2004Apr_61	16.5	3.2	34.1	19.3	11.7	15.2	1
K2, 8 April 2004	2004Apr_62	27.8	2.9	37.1	17.4	6.6	8.2	1
K2, 8 April 2004	2004Apr_63	14.3	3.3	27.6	12.9	13.5	28.4	2
K2, 8 April 2004	2004Apr_64	17.6	2.6	31.0	18.9	10.7	19.2	2
K2, 8 April 2004	2004Apr_65	20.3	2.9	45.3	17.7	5.5	8.3	1
K2, 8 April 2004	2004Apr_66	19.7	2.5	44.4	15.9	6.3	11.2	1
K2, 8 April 2004	2004Apr_67	14.5	1.6	32.5	13.9	11.3	26.2	2
K2, 8 April 2004	2004Apr_68	0.8	0.5	4.8	2.6	19.1	72.3	3
K2, 8 April 2004	2004Apr_69	24.3	2.2	25.3	14.7	10.9	22.6	2
K2, 8 April 2004	2004Apr_70	15.9	1.4	28.5	12.1	12.1	30.0	2
K2, 8 April 2004	2004Apr_71	21.7	2.0	41.5	15.5	6.5	12.6	1
K2, 8 April 2004	2004Apr_72	15.4	2.2	28.1	12.9	9.5	31.9	2
K2, 8 April 2004	2004Apr_73	18.5	1.7	42.8	18.1	7.1	11.8	1
K2, 8 April 2004	2004Apr_74	2.3	0.7	7.6	4.6	18.1	66.7	3
K2, 8 April 2004	2004Apr_75	20.1	2.6	48.6	15.9	4.2	8.6	1
K2, 8 April 2004	2004Apr_76	18.1	3.0	35.9	16.1	7.5	19.4	1
K2, 8 April 2004	2004Apr_77	14.3	2.2	36.5	12.4	10.0	24.5	2
K2, 8 April 2004	2004Apr_78	17.5	2.5	39.6	14.5	9.2	16.6	1
K2, 8 April 2004	2004Apr_79	14.7	2.2	38.4	15.8	10.3	18.6	1
K2, 8 April 2004	2004Apr_80	23.8	2.7	27.2	16.7	13.4	16.1	2
K2, 8 April 2004	2004Apr_81	21.5	2.4	42.6	16.3	6.4	10.8	1
K2, 8 April 2004	2004Apr_82	13.9	2.3	30.4	14.4	15.5	23.4	2
K2, 8 April 2004	2004Apr_83	0.6	0.6	2.3	3.1	21.3	72.2	3
K2, 8 April 2004	2004Apr_84	13.8	2.0	38.2	15.5	11.4	19.1	1
K2, 8 April 2004	2004Apr_85	17.5	2.5	42.0	16.7	7.8	13.6	1
K2, 8 April 2004	2004Apr_86	20.2	2.6	45.0	15.0	5.2	11.9	1
K2, 8 April 2004	2004Apr_87	20.8	2.8	36.4	15.2	7.9	16.9	1
K2, 8 April 2004	2004Apr_88	7.0	2.5	23.6	7.1	5.8	54.0	3
K2, 8 April 2004	2004Apr_89	7.4	1.4	22.5	8.4	12.7	47.6	3
K2, 8 April 2004	2004Apr_90	14.6	2.2	29.5	14.5	13.8	25.4	2
K2, 8 April 2004	2004Apr_91	19.8	2.3	36.0	14.9	10.1	16.9	1
K2, 8 April 2004	2004Apr_92	16.3	2.5	36.4	12.7	8.0	24.1	2
K2, 8 April 2004	2004Apr_93	21.0	2.4	46.6	15.6	4.5	9.9	1
K2, 8 April 2004	2004Apr_94	6.4	1.0	8.7	16.4	30.5	37.1	3
K2, 8 April 2004	2004Apr_95	18.0	2.8	41.1	15.2	8.6	14.3	1
K2, 8 April 2004	2004Apr_96	2.8	1.0	7.4	6.2	19.2	63.4	3
K2, 8 April 2004	2004Apr_97	18.1	2.6	29.4	15.3	11.4	23.2	2
K2, 8 April 2004	2004Apr_98	19.8	2.7	46.5	15.4	6.6	9.0	1
K2, 8 April 2004	2004Apr_99	16.4	2.7	34.0	13.6	8.3	25.0	2
K2, 8 April 2004	2004Apr_100	21.3	2.1	42.4	14.7	6.4	13.1	1
K2, 8 April 2004	2004Apr_101	22.3	3.3	37.3	15.1	5.8	16.3	1
K2, 8 April 2004	2004Apr_102	4.9	1.0	12.1	5.7	17.8	58.5	3
K2, 8 April 2004	2004Apr_103	17.2	3.5	21.9	15.0	14.8	27.5	2
K2, 8 April 2004	2004Apr_104	18.9	2.9	43.0	14.0	8.4	12.8	1
K2, 8 April 2004	2004Apr_105	17.8	2.6	38.6	14.8	10.5	15.8	1
K2, 8 April 2004	2004Apr_106	15.4	2.3	34.3	10.2	10.4	27.4	2
K2, 8 April 2004	2004Apr_107	16.6	3.3	29.0	16.0	20.7	14.5	2
K2, 8 April 2004	2004Apr_108	30.9	3.6	31.3	14.6	7.3	12.2	1
K2, 8 April 2004	2004Apr_109	12.8	2.6	27.1	10.8	14.1	32.6	2
K2, 8 April 2004	2004Apr_110	19.0	2.5	40.7	14.9	9.2	13.6	1
K2, 8 April 2004	2004Apr_111	12.8	1.9	30.4	11.0	12.4	31.5	2
K2, 8 April 2004	2004Apr_112	12.7	4.4	7.0	13.8	25.3	36.8	2
K2, 8 April 2004	2004Apr_113	18.4	3.0	37.7	11.0	9.1	20.8	1
K2, 8 April 2004	2004Apr_114	23.1	3.4	39.4	13.5	7.2	13.4	1
K2, 8 April 2004	2004Apr_115	25.5	2.8	39.0	14.1	7.0	11.7	1
K2, 8 April 2004	2004Apr_116	3.7	1.2	12.3	4.9	18.0	59.9	3
K2, 8 April 2004	2004Apr_117	21.9	3.2	39.1	13.4	8.7	13.7	1
K2, 8 April 2004	2004Apr_118	24.2	3.6	37.2	14.0	8.1	12.9	1
K2, 8 April 2004	2004Apr_119	21.0	2.3	24.6	12.0	12.2	27.8	2
K2, 8 April 2004	2004Apr_120	24.5	2.9	43.8	13.5	5.9	9.5	1
K2, 8 April 2004	2004Apr_121	23.9	2.5	32.9	14.0	11.1	15.6	1
K2, 8 April 2004	2004Apr_122	9.6	4.2	32.9	9.5	8.3	35.5	2
K2, 8 April 2004	2004Apr_123	18.2	2.5	36.4	12.9	10.7	19.3	1
K2, 8 April 2004	2004Apr_124	21.1	3.0	37.4	15.2	9.9	13.4	1
K2, 8 April 2004	2004Apr_125	23.3	2.4	46.2	15.6	4.8	7.7	1
K2, 8 April 2004	2004Apr_126	21.6	2.5	32.2	14.3	11.4	18.0	1
K2, 8 April 2004	2004Apr_127	15.4	2.9	30.0	10.6	13.1	27.9	2
K2, 8 April 2004	2004Apr_128	22.1	3.0	40.8	15.2	7.1	11.8	1
K2, 8 April 2004	2004Apr_129	20.2	2.7	38.4	14.4	10.2	14.2	1
K2, 8 April 2004	2004Apr_130	5.8	2.6	7.7	14.5	25.1	44.2	3
K2, 8 April 2004	2004Apr_131	17.9	4.1	19.6	11.6	17.0	29.8	2
K2, 8 April 2004	2004Apr_132	0.3	0.5	1.2	2.8	23.2	72.0	3

K2, 8 April 2004	2004Apr_133	17.1	2.1	31.2	10.7	0.0	38.9	2
K2, 8 April 2004	2004Apr_134	12.7	2.1	28.6	10.8	13.6	32.2	2
K2, 8 April 2004	2004Apr_135	24.2	2.9	40.1	13.5	6.9	12.4	1
K2, 8 April 2004	2004Apr_136	3.1	1.2	10.6	4.7	19.1	61.3	3
K2, 8 April 2004	2004Apr_137	22.4	3.0	35.7	14.6	9.4	14.9	1
K2, 8 April 2004	2004Apr_138	26.6	3.3	26.8	16.4	9.9	17.0	1
K2, 8 April 2004	2004Apr_139	26.5	2.3	41.2	14.8	6.0	9.2	1
K2, 8 April 2004	2004Apr_140	16.6	2.0	22.1	12.0	15.7	31.6	2
K2, 8 April 2004	2004Apr_141	16.7	2.8	38.0	15.1	10.7	16.7	1
K2, 8 April 2004	2004Apr_142	19.2	2.9	32.9	15.1	10.5	19.5	1
K2, 8 April 2004	2004Apr_143	23.5	3.1	38.8	14.4	6.9	13.4	1
K2, 8 April 2004	2004Apr_144	14.1	2.0	14.4	12.1	21.7	35.7	2
K2, 8 April 2004	2004Apr_145	16.1	2.7	33.6	13.5	13.1	21.1	2
K2, 8 April 2004	2004Apr_146	22.6	3.1	32.4	13.6	10.3	18.0	1
K2, 8 April 2004	2004Apr_147	19.3	2.1	37.2	21.8	7.6	12.0	1
K2, 8 April 2004	2004Apr_148	24.9	2.0	27.7	19.7	8.8	16.9	1
K2, 8 April 2004	2004Apr_149	14.2	1.8	40.1	17.3	9.8	16.8	1
K2, 8 April 2004	2004Apr_150	0.0	0.5	0.0	3.9	18.5	77.1	3
K2, 8 April 2004	2004Apr_151	16.9	2.2	41.0	18.3	7.3	14.4	1
K2, 8 April 2004	2004Apr_152	18.2	2.1	44.2	17.4	7.1	10.9	1
K2, 8 April 2004	2004Apr_153	12.8	2.1	35.3	16.8	11.1	21.9	2
K2, 8 April 2004	2004Apr_154	2.5	4.4	16.5	11.2	20.5	44.9	3
K2, 8 April 2004	2004Apr_155	17.4	2.2	40.5	19.6	7.0	13.4	1
K2, 8 April 2004	2004Apr_156	17.0	2.1	41.1	18.4	8.4	13.0	1
K2, 8 April 2004	2004Apr_157	14.4	2.0	29.4	16.2	12.7	25.2	2
K2, 8 April 2004	2004Apr_158	14.6	2.0	40.6	17.4	9.7	15.7	1
K2, 8 April 2004	2004Apr_159	15.2	1.9	41.7	18.6	8.3	14.3	1
K2, 8 April 2004	2004Apr_160	0.0	0.0	0.0	5.3	30.2	64.6	3
K2, 8 April 2004	2004Apr_161	15.7	1.8	40.4	18.8	8.1	15.2	1
K2, 8 April 2004	2004Apr_162	15.4	1.2	11.5	9.6	22.6	39.6	2
K2, 8 April 2004	2004Apr_163	20.5	2.2	19.9	16.6	14.7	26.1	2
K2, 8 April 2004	2004Apr_164	17.1	2.0	38.0	18.2	7.6	17.0	1
K2, 8 April 2004	2004Apr_165	16.6	2.3	27.2	15.8	11.9	26.2	2
K2, 8 April 2004	2004Apr_166	14.7	1.9	40.7	18.2	9.2	15.2	1
K2, 8 April 2004	2004Apr_167	12.4	1.7	36.7	17.0	7.4	24.8	2
K2, 8 April 2004	2004Apr_168	15.4	2.4	40.6	16.8	8.8	16.0	1
K2, 8 April 2004	2004Apr_169	21.3	1.7	40.8	18.9	6.5	10.7	1
K2, 8 April 2004	2004Apr_170	0.1	0.5	0.0	8.2	33.3	57.8	3
K2, 8 April 2004	2004Apr_171	23.1	1.4	17.7	11.3	15.0	31.5	2
K2, 8 April 2004	2004Apr_172	1.3	1.0	2.1	8.7	21.0	66.0	3
K2, 8 April 2004	2004Apr_173	11.2	1.9	28.3	15.2	14.1	29.2	2
K2, 8 April 2004	2004Apr_174	8.6	2.9	33.6	16.5	12.9	25.4	2
K2, 8 April 2004	2004Apr_175	13.1	1.7	30.0	16.8	11.0	27.4	2
K2, 8 April 2004	2004Apr_176	12.8	2.1	32.7	16.7	12.1	23.6	2
K2, 8 April 2004	2004Apr_177	0.6	1.6	0.0	11.0	36.4	50.4	3
K2, 8 April 2004	2004Apr_178	13.2	1.7	32.3	18.8	11.7	22.3	2
K2, 8 April 2004	2004Apr_179	15.5	2.0	38.0	18.6	7.8	18.1	1
K2, 8 April 2004	2004Apr_180	0.7	0.5	3.0	3.6	19.5	72.8	3
K2, 8 April 2004	2004Apr_181	15.7	1.9	36.4	18.1	10.4	17.4	1
K2, 8 April 2004	2004Apr_182	18.1	2.1	41.8	19.6	6.8	11.6	1
K2, 8 April 2004	2004Apr_183	16.1	1.9	46.7	18.7	4.7	12.0	1
K2, 8 April 2004	2004Apr_184	16.6	2.0	43.1	18.0	6.3	14.1	1
K2, 8 April 2004	2004Apr_185	16.7	1.7	36.9	16.9	5.4	22.4	1
K2, 8 April 2004	2004Apr_186	16.5	2.1	31.7	17.7	11.0	21.0	2
K2, 8 April 2004	2004Apr_187	15.3	1.8	42.9	19.4	7.8	12.9	1
K2, 8 April 2004	2004Apr_188	13.1	1.7	24.7	14.3	13.0	33.2	2
K2, 8 April 2004	2004Apr_189	17.6	3.4	38.0	17.7	7.3	15.9	1
K2, 8 April 2004	2004Apr_190	19.8	2.1	10.4	14.4	17.5	35.9	2
K2, 8 April 2004	2004Apr_191	17.7	1.9	37.6	18.4	8.8	15.6	1
K2, 8 April 2004	2004Apr_192	14.3	2.4	43.8	18.2	6.0	15.3	1
K2, 8 April 2004	2004Apr_193	12.1	2.0	30.4	14.4	11.5	29.6	2
K2, 8 April 2004	2004Apr_194	14.0	1.9	39.8	17.8	9.1	17.3	1
K2, 8 April 2004	2004Apr_195	18.8	2.9	36.1	12.4	10.8	19.1	1
K2, 8 April 2004	2004Apr_196	20.9	2.2	30.7	18.5	10.4	17.2	1
K2, 8 April 2004	2004Apr_197	16.8	2.1	27.1	17.9	11.5	24.5	2
K2, 8 April 2004	2004Apr_198	22.0	2.1	43.9	18.6	4.8	8.6	1
K2, 27 May 2004	2004May_1	2.3	1.5	0.0	14.6	29.8	51.8	3
K2, 27 May 2004	2004May_2	0.3	0.2	0.0	4.6	23.6	71.3	3
K2, 27 May 2004	2004May_3	0.6	1.0	1.0	8.1	22.4	67.0	3
K2, 27 May 2004	2004May_4	0.7	0.5	0.0	8.4	29.4	60.9	3
K2, 27 May 2004	2004May_5	23.8	2.8	41.0	13.1	8.6	10.6	1
K2, 27 May 2004	2004May_6	23.0	2.7	39.4	13.7	7.8	13.3	1
K2, 27 May 2004	2004May_7	10.4	3.7	14.1	13.0	21.8	37.0	2
K2, 27 May 2004	2004May_8	9.1	2.7	15.0	13.3	21.8	38.2	2
K2, 27 May 2004	2004May_9	19.4	2.1	43.7	18.2	8.2	8.4	1
K2, 27 May 2004	2004May_10	14.4	2.4	30.4	11.1	12.1	29.7	2
K2, 27 May 2004	2004May_11	20.1	2.9	50.6	14.1	4.9	7.5	1
K2, 27 May 2004	2004May_12	19.1	2.8	32.9	11.9	11.7	21.5	2
K2, 27 May 2004	2004May_13	1.9	0.3	0.0	7.7	27.5	62.7	3
K2, 27 May 2004	2004May_14	2.1	3.0	0.3	9.2	35.1	50.4	3
K2, 27 May 2004	2004May_15	0.1	0.0	0.0	2.9	19.2	77.9	3
K2, 27 May 2004	2004May_16	24.2	2.4	37.6	18.2	6.2	11.3	1
K2, 27 May 2004	2004May_17	22.4	3.0	44.4	14.1	5.9	10.3	1
K2, 27 May 2004	2004May_18	0.8	0.5	0.0	8.9	30.6	59.2	3
K2, 27 May 2004	2004May_19	1.5	0.5	0.0	9.7	30.9	57.3	3
K2, 27 May 2004	2004May_20	24.1	3.2	30.4	23.7	7.5	11.1	1

K2, 27 May 2004	2004May_21	1.6	0.9	0.0	12.0	29.3	56.2	3
K2, 27 May 2004	2004May_22	19.3	2.5	46.0	13.6	7.2	11.4	1
K2, 27 May 2004	2004May_23	14.2	2.9	33.5	10.6	14.1	24.8	2
K2, 27 May 2004	2004May_24	19.2	4.5	20.2	13.0	14.6	28.5	2
K2, 27 May 2004	2004May_25	0.5	0.4	0.0	8.2	27.6	63.3	3
K2, 27 May 2004	2004May_26	0.7	0.9	3.7	2.8	19.9	72.0	3
K2, 27 May 2004	2004May_27	26.2	2.7	38.8	13.2	7.8	11.3	1
K2, 27 May 2004	2004May_28	2.9	1.2	0.0	6.3	23.9	65.8	3
K2, 27 May 2004	2004May_29	22.9	3.0	47.3	12.7	6.1	8.0	1
K2, 27 May 2004	2004May_30	20.4	2.9	45.8	13.2	7.4	10.4	1
K2, 27 May 2004	2004May_31	7.3	2.9	14.5	10.7	21.6	43.0	3
K2, 27 May 2004	2004May_32	1.4	0.3	0.0	5.8	23.8	68.7	3
K2, 27 May 2004	2004May_33	21.3	3.3	43.7	12.3	8.1	11.3	1
K2, 27 May 2004	2004May_34	2.1	0.5	0.0	11.1	33.0	53.3	3
K2, 27 May 2004	2004May_35	5.8	2.1	14.9	7.2	19.2	50.9	3
K2, 27 May 2004	2004May_36	0.5	0.2	0.0	5.6	25.6	68.1	3
K2, 27 May 2004	2004May_37	22.0	3.2	37.3	11.4	11.0	15.1	1
K2, 27 May 2004	2004May_38	15.5	2.7	23.5	14.9	14.4	29.0	2
K2, 27 May 2004	2004May_39	5.8	1.3	13.9	4.6	20.4	54.0	3
K2, 27 May 2004	2004May_40	2.2	1.0	0.0	6.9	20.9	68.9	3
K2, 27 May 2004	2004May_41	16.9	2.4	27.8	13.7	13.4	25.8	2
K2, 27 May 2004	2004May_42	22.5	3.1	38.8	12.1	8.9	14.6	1
K2, 27 May 2004	2004May_43	23.3	2.4	39.3	15.4	7.3	12.2	1
K2, 27 May 2004	2004May_44	0.5	0.3	0.0	7.5	28.4	63.3	3
K2, 27 May 2004	2004May_45	0.0	0.3	0.0	2.1	21.3	76.3	3
K2, 27 May 2004	2004May_46	11.3	1.5	20.2	9.2	17.5	40.3	2
K2, 27 May 2004	2004May_47	18.8	2.6	39.5	14.1	8.7	16.4	1
K2, 27 May 2004	2004May_48	1.2	0.6	0.0	10.5	32.0	55.7	3
K2, 27 May 2004	2004May_49	27.4	3.0	28.6	17.7	8.4	14.9	1
K2, 27 May 2004	2004May_50	0.1	0.4	0.0	8.1	30.1	61.2	3
K2, 27 May 2004	2004May_51	18.6	2.5	32.7	9.3	11.2	25.7	2
K2, 27 May 2004	2004May_52	0.0	0.2	0.0	6.8	25.5	67.5	3
K2, 27 May 2004	2004May_53	13.2	4.6	55.2	2.6	8.6	15.9	1
K2, 27 May 2004	2004May_54	0.0	1.0	0.0	6.2	18.6	74.1	3
K2, 27 May 2004	2004May_55	2.3	1.5	0.0	10.8	32.5	52.8	3
K2, 27 May 2004	2004May_56	0.9	1.2	0.0	4.2	19.5	74.1	3
K2, 27 May 2004	2004May_57	11.5	2.1	15.2	13.0	20.3	37.9	2
K2, 27 May 2004	2004May_58	0.9	2.5	0.0	11.5	35.4	49.7	3
K2, 27 May 2004	2004May_59	7.5	1.7	16.5	9.0	18.0	47.3	3
K2, 27 May 2004	2004May_60	3.7	3.1	11.5	6.4	15.8	59.5	3
K2, 27 May 2004	2004May_61	0.5	0.6	2.4	3.0	23.2	70.3	3
K2, 27 May 2004	2004May_62	11.4	2.2	18.1	11.9	18.8	37.5	2
K2, 17 August 2004	2004Aug_1	13.9	1.9	29.0	10.3	12.3	32.6	2
K2, 17 August 2004	2004Aug_2	22.4	3.0	25.1	12.5	13.5	23.5	2
K2, 17 August 2004	2004Aug_3	23.2	2.3	38.1	19.3	7.7	9.4	1
K2, 17 August 2004	2004Aug_4	22.3	2.8	41.5	14.9	7.1	11.5	1
K2, 17 August 2004	2004Aug_5	22.4	3.0	45.1	13.7	6.4	9.4	1
K2, 17 August 2004	2004Aug_6	18.7	2.9	31.3	15.0	13.8	18.3	2
K2, 17 August 2004	2004Aug_7	0.9	0.2	0.0	4.3	18.9	75.7	3
K2, 17 August 2004	2004Aug_8	22.9	3.0	46.0	14.7	4.8	8.7	1
K2, 17 August 2004	2004Aug_9	13.3	3.1	17.1	14.9	20.6	31.1	2
K2, 17 August 2004	2004Aug_10	21.6	3.2	42.3	14.5	6.8	11.5	1
K2, 17 August 2004	2004Aug_11	0.0	1.0	0.0	8.5	36.2	54.3	3
K2, 17 August 2004	2004Aug_12	1.3	0.5	0.0	6.4	25.3	66.5	3
K2, 17 August 2004	2004Aug_13	0.0	1.3	0.0	7.6	28.1	63.1	3
K2, 17 August 2004	2004Aug_14	0.7	1.9	0.5	7.6	23.7	65.6	3
K2, 17 August 2004	2004Aug_15	0.9	1.4	0.0	10.8	25.5	61.4	3
K2, 17 August 2004	2004Aug_16	20.8	2.5	40.3	13.7	8.1	14.7	1
K2, 17 August 2004	2004Aug_17	3.6	1.4	11.7	5.3	20.1	57.8	3
K2, 17 August 2004	2004Aug_18	19.1	2.9	34.3	13.3	10.5	20.0	1
K2, 17 August 2004	2004Aug_19	20.7	2.9	41.5	13.0	8.3	13.6	1
K2, 17 August 2004	2004Aug_20	20.0	3.1	34.4	12.9	11.0	18.6	1
K2, 17 August 2004	2004Aug_21	21.6	2.9	37.4	12.7	9.1	16.3	1
K2, 17 August 2004	2004Aug_22	23.1	3.0	34.6	14.6	9.6	15.1	1
K2, 17 August 2004	2004Aug_23	0.0	0.6	4.1	2.7	22.5	70.1	3
K2, 17 August 2004	2004Aug_24	10.3	2.7	26.4	7.9	14.4	38.4	2
K2, 17 August 2004	2004Aug_25	19.1	2.3	40.7	14.8	9.2	14.0	1
K2, 17 August 2004	2004Aug_26	13.3	2.4	24.9	11.3	17.7	30.4	2
K2, 17 August 2004	2004Aug_27	18.8	2.3	35.8	15.1	11.5	16.4	1
K2, 17 August 2004	2004Aug_28	22.7	2.9	44.2	13.5	7.5	9.1	1
K2, 17 August 2004	2004Aug_29	18.4	2.6	38.1	12.8	11.0	17.1	1
K2, 17 August 2004	2004Aug_30	23.3	2.9	43.2	15.5	6.5	8.7	1
K2, 17 August 2004	2004Aug_31	7.4	2.6	12.0	12.3	22.3	43.4	3
K2, 17 August 2004	2004Aug_32	9.9	2.1	21.9	11.1	16.3	38.8	2
K2, 17 August 2004	2004Aug_33	16.4	2.7	36.3	12.8	13.6	18.3	1
K2, 17 August 2004	2004Aug_34	24.2	2.8	44.1	14.7	5.9	8.2	1
K2, 17 August 2004	2004Aug_35	15.1	2.9	33.3	11.7	13.9	23.1	2
K2, 17 August 2004	2004Aug_36	17.7	4.2	19.6	12.6	17.0	29.0	2
K2, 17 August 2004	2004Aug_37	21.4	3.1	40.6	11.8	9.0	14.1	1
K2, 17 August 2004	2004Aug_38	17.4	2.6	19.1	11.3	17.0	32.6	2
K2, 17 August 2004	2004Aug_39	24.9	2.7	41.7	12.5	6.9	11.3	1
K2, 17 August 2004	2004Aug_40	26.7	3.2	37.2	13.3	7.8	12.0	1
K2, 17 August 2004	2004Aug_41	11.9	2.0	26.9	7.9	13.8	37.5	2
K2, 17 August 2004	2004Aug_42	0.8	0.5	2.1	3.0	23.3	70.2	3
K2, 17 August 2004	2004Aug_43	0.5	0.1	0.0	4.5	22.3	72.6	3
K2, 17 August 2004	2004Aug_44	20.6	2.8	41.6	14.5	8.4	12.3	1

K2, 17 August 2004	2004Aug_45	9.4	1.6	20.7	7.7	18.8	41.8	2
K2, 17 August 2004	2004Aug_46	20.4	2.9	41.7	13.4	7.0	14.6	1
K2, 17 August 2004	2004Aug_47	10.7	2.3	18.9	12.0	20.4	35.7	2
K2, 17 August 2004	2004Aug_48	25.8	3.2	44.3	12.7	6.3	7.7	1
K2, 17 August 2004	2004Aug_49	16.5	3.1	29.4	11.9	14.6	24.5	2
K2, 17 August 2004	2004Aug_50	21.7	2.9	38.7	13.0	10.8	13.0	1
K2, 17 August 2004	2004Aug_51	22.0	2.8	39.4	13.4	9.1	13.2	1
K2, 17 August 2004	2004Aug_52	21.2	3.1	46.9	13.4	6.2	9.2	1
K2, 17 August 2004	2004Aug_53	22.3	2.9	45.5	13.5	6.6	9.2	1
K2, 17 August 2004	2004Aug_54	12.2	2.3	29.2	8.9	13.6	33.9	2
K2, 17 August 2004	2004Aug_55	15.3	2.7	34.6	11.1	14.9	21.5	2
K2, 17 August 2004	2004Aug_56	0.4	0.0	0.0	4.0	23.3	72.3	3
K2, 17 August 2004	2004Aug_57	0.4	0.2	0.0	5.4	24.2	69.8	3
K2, 17 August 2004	2004Aug_58	17.0	3.7	51.5	13.1	0.5	14.3	1
K2, 17 August 2004	2004Aug_59	23.5	2.6	36.7	16.0	8.9	12.3	1
K2, 17 August 2004	2004Aug_60	18.9	2.9	41.5	10.6	10.7	15.3	1
K2, 17 August 2004	2004Aug_61	24.8	2.7	40.6	15.7	6.5	9.5	1
K2, 17 August 2004	2004Aug_62	22.2	3.0	34.0	14.7	12.4	13.6	1
K2, 17 August 2004	2004Aug_63	4.1	1.1	11.1	4.3	20.0	59.5	3
K2, 17 August 2004	2004Aug_64	20.4	3.3	35.2	11.8	11.0	18.2	1
K2, 17 August 2004	2004Aug_65	19.6	3.0	41.0	12.5	10.3	13.7	1
K2, 17 August 2004	2004Aug_66	17.4	2.5	36.3	14.2	13.7	15.8	1
K2, 17 August 2004	2004Aug_67	21.3	2.5	44.6	15.0	6.9	9.6	1
K2, 17 August 2004	2004Aug_68	17.8	2.4	19.2	19.2	19.5	21.8	2
K2, 17 August 2004	2004Aug_69	21.3	2.1	32.6	12.3	12.0	19.7	1
K2, 17 August 2004	2004Aug_70	22.2	3.2	45.2	12.6	7.3	9.5	1
K2, 17 August 2004	2004Aug_71	11.1	2.1	17.6	14.5	21.6	33.1	2
K2, 17 August 2004	2004Aug_72	23.4	3.1	41.0	14.0	7.9	10.5	1
K2, 17 August 2004	2004Aug_73	21.4	3.4	37.7	14.2	8.3	15.0	1
K2, 17 August 2004	2004Aug_74	28.1	3.0	9.5	14.0	20.8	24.6	2
K2, 17 August 2004	2004Aug_75	25.0	3.0	42.6	12.5	7.8	9.2	1
K2, 17 August 2004	2004Aug_76	30.7	0.7	10.9	16.3	15.2	26.1	2
K2, 17 August 2004	2004Aug_77	5.0	1.3	13.2	4.6	19.1	56.7	3
K2, 17 August 2004	2004Aug_78	22.7	3.4	41.9	12.4	8.4	11.1	1
K2, 17 August 2004	2004Aug_79	11.7	2.5	17.7	15.4	22.1	30.6	2
K2, 17 August 2004	2004Aug_80	5.3	1.6	15.0	5.4	19.0	53.8	3
K2, 17 August 2004	2004Aug_81	22.3	3.1	37.6	12.5	11.6	12.9	1
K2, 17 August 2004	2004Aug_82	23.6	3.0	42.3	12.5	7.4	11.3	1
K2, 17 August 2004	2004Aug_83	0.7	0.3	0.0	4.7	24.2	70.1	3
K2, 17 August 2004	2004Aug_84	17.8	3.3	31.3	11.3	13.4	22.9	2
K2, 17 August 2004	2004Aug_85	19.6	2.9	36.4	14.3	9.5	17.3	1
K2, 17 August 2004	2004Aug_86	15.2	2.6	23.6	12.4	15.7	30.6	2
K2, 17 August 2004	2004Aug_87	24.0	3.1	36.9	14.3	8.8	12.9	1
K2, 17 August 2004	2004Aug_88	0.4	0.6	0.0	4.4	26.1	68.5	3
K2, 17 August 2004	2004Aug_89	0.2	0.0	0.0	3.7	21.7	74.4	3
K2, 17 August 2004	2004Aug_90	8.2	1.7	20.5	11.4	12.2	45.9	2
K2, 17 August 2004	2004Aug_91	15.8	1.0	34.6	15.8	8.2	24.7	2
K2, 17 August 2004	2004Aug_92	0.8	1.4	1.0	9.1	32.0	55.7	3
K2, 17 August 2004	2004Aug_93	22.4	2.0	34.3	18.7	9.1	13.5	1
K2, 17 August 2004	2004Aug_94	5.1	1.6	15.4	10.9	20.1	46.8	3
K2, 17 August 2004	2004Aug_95	18.0	2.1	39.6	18.1	8.0	14.2	1
K2, 17 August 2004	2004Aug_96	1.7	0.7	6.7	4.8	17.8	68.3	3
K2, 17 August 2004	2004Aug_97	15.4	2.1	19.1	17.7	17.2	28.5	2
K2, 17 August 2004	2004Aug_98	20.1	2.0	29.1	17.1	11.8	20.0	2
K2, 17 August 2004	2004Aug_99	6.2	1.0	16.8	9.6	15.2	51.2	3
K2, 17 August 2004	2004Aug_100	9.6	0.9	23.7	11.4	11.6	42.7	2
K2, 17 August 2004	2004Aug_101	9.9	1.9	14.5	13.7	21.5	38.6	2
K2, 17 August 2004	2004Aug_102	16.2	1.5	28.3	17.0	11.0	26.0	2
K2, 17 August 2004	2004Aug_103	11.7	1.8	34.2	17.0	12.9	22.3	2
K2, 17 August 2004	2004Aug_104	17.2	2.3	27.9	18.2	11.8	22.7	2
K2, 17 August 2004	2004Aug_105	0.1	0.2	0.0	3.4	18.9	77.5	3
K2, 17 August 2004	2004Aug_106	20.5	2.1	30.1	22.2	9.7	15.4	1
K2, 17 August 2004	2004Aug_107	23.9	1.6	34.3	23.5	6.8	9.9	1
K2, 17 August 2004	2004Aug_108	4.2	0.5	4.9	6.4	20.3	63.7	3
K2, 17 August 2004	2004Aug_109	17.4	1.9	38.5	19.5	6.1	16.5	1
K2, 17 August 2004	2004Aug_110	12.1	0.8	23.5	14.9	12.5	36.1	2
K2, 17 August 2004	2004Aug_111	18.4	2.3	33.6	21.3	10.8	13.7	1
K2, 17 August 2004	2004Aug_112	14.3	1.9	38.8	17.2	10.7	17.1	1
K2, 17 August 2004	2004Aug_113	3.9	0.9	10.5	6.5	17.8	60.5	3
K2, 17 August 2004	2004Aug_114	17.2	0.6	36.7	23.7	8.6	13.1	1
K2, 17 August 2004	2004Aug_115	5.2	2.2	5.9	14.9	24.3	47.5	3
K2, 17 August 2004	2004Aug_116	21.2	2.7	32.3	19.7	11.0	13.2	1
K2, 17 August 2004	2004Aug_117	26.6	1.7	34.6	20.8	6.6	9.7	1
K2, 17 August 2004	2004Aug_118	3.6	1.7	11.8	7.0	9.4	66.5	3
K2, 17 August 2004	2004Aug_119	25.9	1.7	21.7	22.9	11.0	16.8	2
K2, 17 August 2004	2004Aug_120	1.3	0.2	3.3	2.9	17.4	75.0	3
K2, 13 June 2006	2006Jun13_1	8.7	1.4	19.8	6.9	14.4	48.9	3
K2, 13 June 2006	2006Jun13_2	25.3	0.9	26.1	15.4	12.9	19.3	2
K2, 13 June 2006	2006Jun13_3	19.3	2.5	44.3	17.3	6.8	9.7	1
K2, 13 June 2006	2006Jun13_4	17.2	2.0	28.2	13.8	12.2	26.6	2
K2, 13 June 2006	2006Jun13_5	8.8	1.4	19.8	8.0	15.1	46.9	3
K2, 13 June 2006	2006Jun13_6	22.3	2.4	39.9	15.1	7.8	12.4	1
K2, 13 June 2006	2006Jun13_7	19.7	2.5	2.0	13.5	21.6	40.7	2
K2, 13 June 2006	2006Jun13_8	21.9	2.4	42.6	15.4	6.7	10.9	1
K2, 13 June 2006	2006Jun13_9	24.3	2.5	42.1	15.4	6.3	9.5	1
K2, 13 June 2006	2006Jun13_10	24.1	2.2	41.9	15.8	6.1	10.0	1

K2, 13 June 2006	2006Jun13_11	0.0	0.5	3.7	2.3	19.6	73.9	3
K2, 13 June 2006	2006Jun13_12	22.5	2.8	45.7	15.4	5.1	8.4	1
K2, 13 June 2006	2006Jun13_13	15.4	1.5	12.2	12.0	19.4	39.5	2
K2, 13 June 2006	2006Jun13_14	19.0	2.4	37.8	14.3	8.0	18.5	1
K2, 13 June 2006	2006Jun13_15	25.1	2.1	34.6	14.6	10.0	13.6	1
K2, 13 June 2006	2006Jun13_16	3.3	1.1	10.6	4.5	18.7	61.8	3
K2, 13 June 2006	2006Jun13_17	21.6	2.5	43.2	16.4	6.4	9.9	1
K2, 13 June 2006	2006Jun13_18	20.5	2.1	25.1	15.7	12.5	24.2	2
K2, 13 June 2006	2006Jun13_19	2.5	0.8	6.8	4.2	19.7	65.9	3
K2, 13 June 2006	2006Jun13_20	21.9	2.1	39.6	16.3	7.7	12.4	1
K2, 13 June 2006	2006Jun13_21	25.8	2.3	36.4	15.5	6.7	13.2	1
K2, 13 June 2006	2006Jun13_22	6.1	1.2	13.6	5.6	17.0	56.5	3
K2, 13 June 2006	2006Jun13_23	2.8	0.0	0.0	5.0	38.0	54.2	3
K2, 13 June 2006	2006Jun13_24	3.1	1.0	8.6	4.6	19.0	63.8	3
K2, 13 June 2006	2006Jun13_25	19.2	2.3	38.2	14.2	7.3	18.9	1
K2, 13 June 2006	2006Jun13_26	2.7	1.0	8.2	3.7	18.5	65.9	3
K2, 13 June 2006	2006Jun13_27	0.8	2.9	0.1	10.7	33.0	52.5	3
K2, 13 June 2006	2006Jun13_28	27.0	2.2	37.1	15.8	6.2	11.6	1
K2, 13 June 2006	2006Jun13_29	20.3	1.8	15.8	12.5	15.3	34.4	2
K2, 13 June 2006	2006Jun13_30	25.0	2.5	43.1	15.0	5.6	8.7	1
K2, 13 June 2006	2006Jun13_31	22.0	3.0	40.2	13.2	6.6	15.0	1
K2, 13 June 2006	2006Jun13_32	22.6	2.7	42.3	15.1	5.8	11.5	1
K2, 13 June 2006	2006Jun13_33	23.8	2.0	33.2	15.7	9.3	15.9	1
K2, 13 June 2006	2006Jun13_34	16.5	1.8	16.7	11.8	14.6	38.6	2
K2, 13 June 2006	2006Jun13_35	0.0	0.7	5.2	2.1	20.4	71.6	3
K2, 13 June 2006	2006Jun13_36	29.4	0.6	27.5	12.8	9.3	20.5	2
K2, 13 June 2006	2006Jun13_37	20.6	1.8	26.5	15.0	12.7	23.3	2
K2, 13 June 2006	2006Jun13_38	12.3	2.0	26.7	10.4	13.5	35.1	2
K2, 13 June 2006	2006Jun13_39	15.1	1.6	17.0	11.6	15.9	38.9	2
K2, 13 June 2006	2006Jun13_40	23.5	2.6	40.6	15.8	6.9	10.6	1
K2, 13 June 2006	2006Jun13_41	8.0	1.1	15.8	7.5	15.0	52.6	3
K2, 13 June 2006	2006Jun13_42	22.3	1.5	24.3	16.8	13.1	22.0	2
K2, 13 June 2006	2006Jun13_43	7.4	0.5	9.7	6.8	18.1	57.5	3
K2, 13 June 2006	2006Jun13_44	26.9	0.8	34.4	16.4	7.0	14.5	1
K2, 13 June 2006	2006Jun13_45	10.4	0.9	15.7	9.8	16.4	46.7	3
K2, 13 June 2006	2006Jun13_46	3.7	0.8	9.3	4.5	18.8	62.9	3
K2, 13 June 2006	2006Jun13_47	23.4	2.2	35.9	15.4	9.3	13.8	1
K2, 13 June 2006	2006Jun13_48	5.6	0.2	1.7	7.9	19.8	64.7	3
K2, 13 June 2006	2006Jun13_49	5.8	1.0	14.1	5.8	16.7	56.7	3
K2, 13 June 2006	2006Jun13_50	12.4	2.2	23.0	12.0	15.8	34.5	2
K2, 13 June 2006	2006Jun13_51	18.6	2.8	13.7	16.1	16.3	32.5	2
K2, 13 June 2006	2006Jun13_52	32.7	0.5	31.2	17.3	6.3	12.1	1
K2, 13 June 2006	2006Jun13_53	25.0	2.2	43.7	15.1	5.2	8.7	1
K2, 13 June 2006	2006Jun13_54	20.6	1.5	7.9	14.7	19.1	36.2	2
K2, 13 June 2006	2006Jun13_55	34.4	2.3	22.5	18.2	9.5	13.1	1
K2, 13 June 2006	2006Jun13_56	33.2	0.9	31.8	18.7	6.9	8.4	1
K2, 13 June 2006	2006Jun13_57	1.5	0.5	3.9	3.2	19.8	71.2	3
K2, 13 June 2006	2006Jun13_58	19.8	1.2	24.5	17.1	13.1	24.4	2
K2, 13 June 2006	2006Jun13_59	19.9	0.0	0.0	8.9	27.5	43.7	3
K2, 13 June 2006	2006Jun13_60	28.1	2.3	34.9	18.2	7.2	9.3	1
K2, 13 June 2006	2006Jun13_61	26.8	1.6	20.1	15.4	12.4	23.6	2
K2, 13 June 2006	2006Jun13_62	7.4	1.1	14.2	5.7	17.8	53.8	3
K2, 13 June 2006	2006Jun13_63	4.3	0.7	0.0	11.2	33.1	50.6	3
K2, 13 June 2006	2006Jun13_64	21.5	2.9	30.1	14.6	10.9	20.0	2
K2, 13 June 2006	2006Jun13_65	21.8	2.0	27.9	14.6	10.0	23.7	2
K2, 13 June 2006	2006Jun13_66	8.8	1.3	16.7	6.7	16.5	50.0	3
K2, 13 June 2006	2006Jun13_67	25.1	1.7	34.0	16.1	9.5	13.7	1
K2, 13 June 2006	2006Jun13_68	11.0	1.0	17.1	8.1	15.7	47.1	3
K2, 13 June 2006	2006Jun13_69	31.0	1.0	22.7	16.8	12.8	15.7	2
K2, 13 June 2006	2006Jun13_70	21.2	2.6	38.9	11.7	11.3	14.2	1
K2, 13 June 2006	2006Jun13_71	23.0	3.0	34.7	13.0	10.8	15.5	1
K2, 13 June 2006	2006Jun13_72	23.8	2.5	36.2	12.5	10.0	15.1	1
K2, 13 June 2006	2006Jun13_73	24.6	2.1	35.2	13.7	11.3	13.1	1
K2, 13 June 2006	2006Jun13_74	24.7	1.3	11.1	17.7	20.2	25.0	2
K2, 13 June 2006	2006Jun13_75	25.9	2.5	42.7	12.3	7.1	9.5	1
K2, 13 June 2006	2006Jun13_76	36.7	2.5	39.9	10.5	5.1	5.3	1
K2, 13 June 2006	2006Jun13_77	23.7	1.3	30.4	10.7	11.1	22.7	2
K2, 13 June 2006	2006Jun13_78	26.5	1.8	26.7	14.3	12.8	17.9	2
K2, 13 June 2006	2006Jun13_79	31.5	0.8	31.3	15.4	8.8	12.1	1
K2, 13 June 2006	2006Jun13_80	28.5	1.3	37.0	12.9	8.7	11.6	1
K2, 13 June 2006	2006Jun13_81	10.7	0.5	14.7	7.0	19.7	47.4	3
K2, 13 June 2006	2006Jun13_82	28.2	2.9	39.7	12.1	8.3	8.7	1
K2, 13 June 2006	2006Jun13_83	29.7	1.9	38.9	13.0	7.2	9.3	1
K2, 13 June 2006	2006Jun13_84	27.8	0.2	26.2	12.0	12.6	21.2	2
K2, 13 June 2006	2006Jun13_85	35.9	1.1	30.5	11.9	8.4	12.2	1
K2, 13 June 2006	2006Jun13_86	7.1	2.1	17.2	4.3	0.0	69.3	3
K2, 13 June 2006	2006Jun13_87	25.4	1.9	40.4	13.6	8.4	10.3	1
K2, 13 June 2006	2006Jun13_88	30.0	1.6	32.6	15.6	9.1	11.1	1
K2, 13 June 2006	2006Jun13_89	29.4	1.1	41.3	13.3	5.1	9.8	1
K2, 13 June 2006	2006Jun13_90	33.4	1.7	33.1	13.9	8.0	10.0	1
K2, 13 June 2006	2006Jun13_91	27.7	0.8	10.7	14.7	18.3	27.8	2
K2, 13 June 2006	2006Jun13_92	28.3	3.0	29.1	14.6	10.3	14.7	1
K2, 13 June 2006	2006Jun13_93	27.4	1.6	32.7	15.1	10.0	13.3	1
K2, 13 June 2006	2006Jun13_94	17.9	0.4	8.7	9.8	18.5	44.7	3
K2, 13 June 2006	2006Jun13_95	24.9	1.9	38.2	16.6	7.9	10.4	1
K2, 13 June 2006	2006Jun13_96	13.4	1.2	25.2	11.0	13.8	35.4	2

K2, 13 June 2006	2006Jun13_97	20.1	1.9	37.7	14.6	9.5	16.3	1
K2, 13 June 2006	2006Jun13_98	21.0	1.3	33.3	19.6	10.0	14.8	1
K2, 13 June 2006	2006Jun13_99	25.7	1.2	36.7	18.2	8.0	10.2	1
K2, 13 June 2006	2006Jun13_100	25.9	1.9	46.0	13.8	5.1	7.2	1
K2, 13 June 2006	2006Jun13_101	5.2	0.6	8.7	6.6	20.6	58.3	3
K2, 13 June 2006	2006Jun13_102	6.7	0.9	15.5	8.3	18.6	50.0	3
K2, 13 June 2006	2006Jun13_103	0.0	0.7	7.4	5.4	23.4	65.6	3
K2, 13 June 2006	2006Jun13_104	21.0	1.1	34.2	16.6	10.0	17.2	1
K2, 13 June 2006	2006Jun13_105	22.9	0.9	16.1	15.7	15.8	28.7	2
K2, 13 June 2006	2006Jun13_106	21.9	2.0	37.2	18.6	8.1	12.2	1
K2, 13 June 2006	2006Jun13_107	23.1	0.5	23.3	17.3	15.6	20.3	2
K2, 13 June 2006	2006Jun13_108	19.4	1.7	46.2	18.4	4.7	9.5	1
K2, 13 June 2006	2006Jun13_109	39.0	0.0	2.0	14.9	18.5	25.6	2
K2, 13 June 2006	2006Jun13_110	32.2	0.5	34.1	17.3	7.1	8.9	1
K2, 13 June 2006	2006Jun13_111	3.5	0.5	7.2	6.3	22.7	59.8	3
K2, 13 June 2006	2006Jun13_112	25.5	1.8	32.2	17.8	10.3	12.5	1
K2, 13 June 2006	2006Jun13_113	26.7	0.7	36.4	18.6	7.8	9.8	1
K2, 13 June 2006	2006Jun13_114	9.5	0.5	11.7	8.9	20.9	48.5	3
K2, 13 June 2006	2006Jun13_115	6.5	0.4	8.5	6.5	21.7	56.4	3
K2, 13 June 2006	2006Jun13_116	20.1	1.3	39.7	18.2	7.0	13.8	1
K2, 13 June 2006	2006Jun13_117	18.2	1.1	35.6	19.1	10.8	15.2	1
K2, 13 June 2006	2006Jun13_118	23.3	1.2	24.8	20.0	11.9	18.9	2
K2, 13 June 2006	2006Jun13_119	15.8	1.7	41.6	17.7	9.3	13.9	1
K2, 13 June 2006	2006Jun13_120	2.6	0.6	8.3	5.6	19.6	63.3	3
K2, 13 June 2006	2006Jun13_121	17.3	1.0	31.8	18.2	13.7	18.0	2
K2, 13 June 2006	2006Jun13_122	3.7	0.5	7.9	6.3	20.9	60.6	3
K2, 13 June 2006	2006Jun13_123	18.3	0.8	34.2	18.1	9.5	18.9	1
K2, 13 June 2006	2006Jun13_124	24.4	0.7	24.5	23.4	11.8	15.2	2
K2, 13 June 2006	2006Jun13_125	18.8	1.5	37.2	22.4	8.6	11.4	1
K2, 13 June 2006	2006Jun13_126	14.5	1.1	30.9	17.7	14.1	21.8	2
K2, 13 June 2006	2006Jun13_127	17.6	1.0	16.1	20.1	18.4	26.7	2
K2, 13 June 2006	2006Jun13_128	20.0	1.2	36.6	24.8	7.4	10.1	1
K2, 13 June 2006	2006Jun13_129	7.0	0.7	17.4	9.0	16.0	49.9	3
K2, 13 June 2006	2006Jun13_130	13.3	0.8	30.9	18.9	11.1	25.1	2
K2, 13 June 2006	2006Jun13_131	18.2	1.3	42.9	22.1	6.2	9.4	1
K2, 13 June 2006	2006Jun13_132	22.5	0.9	36.5	20.3	8.2	11.6	1
K2, 13 June 2006	2006Jun13_133	18.3	0.4	35.9	18.7	8.7	17.9	1
K2, 13 June 2006	2006Jun13_134	6.8	2.4	24.9	11.9	15.2	38.8	2
K2, 13 June 2006	2006Jun13_135	10.3	0.8	26.5	14.5	14.5	33.3	2
K2, 13 June 2006	2006Jun13_136	9.8	1.0	19.6	12.9	19.3	37.4	2
K2, 13 June 2006	2006Jun13_137	2.2	0.4	8.1	5.4	19.8	64.1	3
K2, 13 June 2006	2006Jun13_138	15.2	1.0	27.5	16.0	13.1	27.3	2
K2, 13 June 2006	2006Jun13_139	14.7	1.5	38.4	18.4	11.6	15.5	1
K2, 13 June 2006	2006Jun13_140	14.3	0.8	40.3	21.2	7.9	15.5	1
K2, 13 June 2006	2006Jun13_141	13.0	0.0	20.7	13.0	13.8	39.5	2
K2, 13 June 2006	2006Jun13_142	20.8	1.1	33.7	20.4	9.5	14.5	1
K2, 13 June 2006	2006Jun13_143	4.5	0.5	10.6	6.7	19.7	58.0	3
K2, 13 June 2006	2006Jun13_144	14.7	0.9	31.0	14.4	12.2	26.8	2
K2, 13 June 2006	2006Jun13_145	7.5	0.7	16.8	10.3	17.0	47.7	3
K2, 13 June 2006	2006Jun13_146	23.1	0.0	15.4	19.3	15.4	26.7	2
K2, 13 June 2006	2006Jun13_147	25.1	0.5	34.5	20.8	6.6	12.6	1
K2, 13 June 2006	2006Jun13_148	12.6	1.5	13.6	16.7	19.7	35.8	2
K2, 13 June 2006	2006Jun13_149	22.4	1.5	37.5	20.6	8.2	9.9	1
K2, 13 June 2006	2006Jun13_150	20.1	0.1	17.4	17.7	15.5	29.2	2
K2, 13 June 2006	2006Jun13_151	15.5	0.8	32.6	18.4	10.5	22.2	2
K2, 13 June 2006	2006Jun13_152	0.0	0.0	0.0	8.1	40.9	51.0	3
K2, 13 June 2006	2006Jun13_153	20.4	1.2	39.9	19.0	9.1	10.3	1
K2, 13 June 2006	2006Jun13_154	18.1	0.9	39.2	22.4	7.7	11.7	1
K2, 13 June 2006	2006Jun13_155	15.6	0.8	48.8	23.3	4.0	7.6	1
K2, 13 June 2006	2006Jun13_156	16.8	0.0	19.0	15.5	15.8	32.9	2
K2, 13 June 2006	2006Jun13_157	16.2	1.1	40.5	21.5	7.7	12.9	1
K2, 13 June 2006	2006Jun13_158	14.2	0.3	21.5	15.4	15.5	33.1	2
K2, 13 June 2006	2006Jun13_159	15.0	1.2	30.7	16.8	10.0	26.2	2
K2, 13 June 2006	2006Jun13_160	17.2	0.5	42.0	21.4	7.0	12.0	1
K2, 13 June 2006	2006Jun13_161	17.0	0.1	15.3	15.7	18.2	33.7	2
K2, 13 June 2006	2006Jun13_162	19.0	0.7	37.3	20.3	9.1	13.5	1
K2, 13 June 2006	2006Jun13_163	23.4	0.2	36.7	18.9	8.0	12.7	1
K2, 13 June 2006	2006Jun13_164	20.5	1.1	38.5	20.6	8.3	11.1	1
K2, 13 June 2006	2006Jun13_165	19.4	1.1	44.3	19.9	6.2	9.1	1
K2, 13 June 2006	2006Jun13_166	18.7	1.0	39.6	20.0	8.4	12.2	1
K2, 13 June 2006	2006Jun13_167	21.9	0.8	34.0	22.2	8.7	12.4	1
K2, 13 June 2006	2006Jun13_168	19.6	0.6	16.8	18.7	17.7	26.6	2
K2, 13 June 2006	2006Jun13_169	6.6	0.2	14.4	9.6	16.3	52.9	3
K2, 13 June 2006	2006Jun13_170	16.6	0.4	28.9	18.1	11.5	24.5	2
K2, 13 June 2006	2006Jun13_171	23.2	0.3	14.7	21.7	17.0	23.1	2
K2, 13 June 2006	2006Jun13_172	21.2	0.9	38.5	23.6	6.5	9.2	1
K2, 13 June 2006	2006Jun13_173	15.4	1.1	20.3	20.3	12.0	30.9	2
K2, 13 June 2006	2006Jun13_174	1.6	0.5	7.1	4.8	17.8	68.2	3
K2, 13 June 2006	2006Jun13_175	11.6	1.2	31.2	18.7	14.2	23.1	2
K2, 13 June 2006	2006Jun13_176	19.5	1.5	36.7	21.8	8.2	12.4	1
K2, 13 June 2006	2006Jun13_177	19.1	1.1	35.6	21.2	10.6	12.3	1
K2, 13 June 2006	2006Jun13_178	13.1	0.6	18.5	14.2	15.7	37.9	2
K2, 13 June 2006	2006Jun13_179	12.5	1.3	21.7	15.9	15.2	33.5	2
K2, 13 June 2006	2006Jun13_180	22.3	1.3	41.2	21.5	7.0	6.7	1
K2, 13 June 2006	2006Jun13_181	21.4	1.2	31.1	21.5	12.1	12.7	1
K2, 13 June 2006	2006Jun13_182	0.0	0.0	0.0	4.0	21.5	74.5	3

K2, 13 June 2006	2006Jun13_183	1.1	0.4	4.2	4.1	21.0	69.2	3
K2, 13 June 2006	2006Jun13_184	19.9	1.1	32.8	21.3	8.7	16.2	1
K2, 13 June 2006	2006Jun13_185	23.6	0.0	24.9	18.7	12.3	20.5	2
K2, 13 June 2006	2006Jun13_186	1.8	0.3	4.0	3.9	22.1	67.9	3
K2, 13 June 2006	2006Jun13_187	20.6	0.6	31.3	19.8	10.7	16.9	1
K2, 13 June 2006	2006Jun13_188	15.4	0.2	14.1	15.8	19.5	34.9	2
K2, 13 June 2006	2006Jun13_189	9.9	0.6	17.2	11.4	17.6	43.4	2
K2, 13 June 2006	2006Jun13_190	7.9	0.1	17.1	11.0	15.6	48.3	3
K2, 13 June 2006	2006Jun13_191	18.8	1.5	33.7	18.3	9.4	18.2	1
K2, 13 June 2006	2006Jun13_192	21.3	0.2	20.6	25.4	12.3	20.3	2
K2, 13 June 2006	2006Jun13_193	19.1	0.1	16.4	15.1	15.5	33.7	2
K2, 13 June 2006	2006Jun13_194	28.9	0.0	15.6	19.6	13.7	22.2	2
K2, 13 June 2006	2006Jun13_195	22.3	0.3	24.2	19.4	13.3	20.5	2
K2, 13 June 2006	2006Jun13_196	18.8	0.0	22.8	16.8	13.2	28.4	2
K2, 13 June 2006	2006Jun13_197	18.0	1.0	35.5	20.4	10.8	14.4	1
K2, 13 June 2006	2006Jun13_198	26.5	0.5	15.9	23.2	13.6	20.2	2
K2, 13 June 2006	2006Jun13_199	1.7	0.4	5.3	4.7	20.5	67.3	3
K2, 13 June 2006	2006Jun13_200	27.3	0.2	7.3	18.3	19.5	27.4	2
K2, 13 June 2006	2006Jun13_201	18.7	0.8	41.2	22.7	6.7	9.9	1
K2, 13 June 2006	2006Jun13_202	4.1	0.2	5.0	6.6	20.4	63.8	3
K2, 13 June 2006	2006Jun13_203	12.8	0.5	17.8	16.9	16.9	35.0	2
K2, 13 June 2006	2006Jun13_204	21.2	0.2	10.7	20.1	16.7	31.0	2
K2, 13 June 2006	2006Jun13_205	25.8	0.8	28.7	23.4	9.3	11.9	1
K2, 13 June 2006	2006Jun13_206	15.9	1.4	18.4	22.6	13.6	28.1	2
K2, 13 June 2006	2006Jun13_207	15.4	1.1	26.4	24.8	8.6	23.8	2
K2, 13 June 2006	2006Jun13_208	6.6	0.5	9.4	11.8	19.1	52.6	3
K2, 13 June 2006	2006Jun13_209	13.8	1.5	44.9	25.2	4.7	10.0	1
K2, 13 June 2006	2006Jun13_210	4.3	0.8	7.1	10.8	18.0	59.0	3
K2, 13 June 2006	2006Jun13_211	18.6	0.9	41.4	21.9	6.5	10.7	1
K2, 13 June 2006	2006Jun13_212	6.8	0.5	13.2	9.2	16.4	53.9	3
K2, 13 June 2006	2006Jun13_213	14.0	1.5	39.2	19.2	10.6	15.4	1
K2, 13 June 2006	2006Jun13_214	2.8	0.5	8.8	6.2	19.7	62.1	3
K2, 13 June 2006	2006Jun13_215	19.1	1.4	43.9	21.6	5.6	8.5	1
K2, 13 June 2006	2006Jun13_216	18.2	1.4	39.8	19.0	9.2	12.4	1
K2, 13 June 2006	2006Jun13_217	10.7	2.1	27.5	14.3	10.2	35.2	2
K2, 13 June 2006	2006Jun13_218	2.2	0.5	7.8	5.6	19.4	64.5	3
K2, 13 June 2006	2006Jun13_219	2.0	0.0	1.1	3.8	20.8	72.3	3
K2, 13 June 2006	2006Jun13_220	20.2	0.2	38.7	22.8	7.5	10.6	1
K2, 13 June 2006	2006Jun13_221	6.4	0.6	14.0	8.3	16.7	54.0	3
K2, 13 June 2006	2006Jun13_222	25.4	0.1	23.7	20.9	11.5	18.4	2
K2, 13 June 2006	2006Jun13_223	30.7	1.2	20.2	24.0	10.3	13.6	2
K2, 13 June 2006	2006Jun13_224	17.0	0.1	36.9	22.6	7.0	16.5	1
K2, 13 June 2006	2006Jun13_225	15.5	1.3	40.9	21.5	7.6	13.2	1
K2, 13 June 2006	2006Jun13_226	8.0	0.4	15.1	11.5	17.8	47.1	3
K2, 13 June 2006	2006Jun13_227	35.9	0.0	14.7	21.7	10.4	17.3	2
K2, 13 June 2006	2006Jun13_228	8.8	0.7	16.7	11.8	14.7	47.3	3
K2, 13 June 2006	2006Jun13_229	20.5	1.1	40.2	20.1	7.3	10.7	1
K2, 13 June 2006	2006Jun13_230	20.0	1.7	19.4	13.9	13.6	31.5	2
K2, 13 June 2006	2006Jun13_231	22.5	2.7	39.4	16.1	6.5	12.9	1
K2, 13 June 2006	2006Jun13_232	22.1	2.0	36.3	16.8	5.8	17.0	1
K2, 13 June 2006	2006Jun13_233	6.2	0.9	13.4	6.1	14.7	58.7	3
K2, 13 June 2006	2006Jun13_234	3.4	0.5	5.9	4.5	16.9	68.8	3
K2, 13 June 2006	2006Jun13_235	19.5	2.1	42.7	16.4	6.0	13.2	1
K2, 13 June 2006	2006Jun13_236	19.1	1.1	21.8	16.3	13.1	28.7	2
K2, 13 June 2006	2006Jun13_237	15.4	1.9	36.1	14.5	8.9	23.1	2
K2, 13 June 2006	2006Jun13_238	9.4	1.0	18.7	9.1	12.5	49.3	3
K2, 13 June 2006	2006Jun13_239	5.7	0.6	12.0	6.5	15.7	59.4	3
K2, 13 June 2006	2006Jun13_240	22.6	2.2	39.8	17.5	6.0	12.0	1
K2, 13 June 2006	2006Jun13_241	13.2	1.4	4.3	12.1	21.5	47.5	3
K2, 13 June 2006	2006Jun13_242	18.1	2.2	38.2	16.1	8.4	17.1	1
K2, 13 June 2006	2006Jun13_243	13.9	1.4	29.4	13.4	11.5	30.3	2
K2, 13 June 2006	2006Jun13_244	20.3	1.5	30.9	16.9	10.6	19.8	2
K2, 13 June 2006	2006Jun13_245	0.0	0.5	0.0	8.0	30.3	61.2	3
K2, 13 June 2006	2006Jun13_246	20.5	2.3	38.9	17.7	6.7	13.8	1
K2, 13 June 2006	2006Jun13_247	10.2	1.1	20.8	12.2	14.7	41.0	2
K2, 13 June 2006	2006Jun13_248	17.7	1.5	39.8	17.7	6.0	17.4	1
K2, 13 June 2006	2006Jun13_249	22.9	2.1	37.0	18.8	6.2	13.1	1
K2, 13 June 2006	2006Jun13_250	6.7	0.9	14.6	7.0	13.4	57.2	3
K2, 13 June 2006	2006Jun13_251	9.1	0.3	11.2	7.4	15.2	56.7	3
K2, 13 June 2006	2006Jun13_252	20.0	1.9	10.5	14.8	17.0	35.9	2
K2, 13 June 2006	2006Jun13_253	19.1	2.1	41.9	16.0	5.3	15.6	1
K2, 13 June 2006	2006Jun13_254	0.0	0.2	0.0	7.5	33.8	58.5	3
K2, 13 June 2006	2006Jun13_255	19.9	1.9	42.0	18.7	4.8	12.8	1
K2, 13 June 2006	2006Jun13_256	15.9	1.7	32.6	13.2	8.6	28.0	2
K2, 13 June 2006	2006Jun13_257	15.9	2.2	39.4	15.9	9.1	17.6	1
K2, 13 June 2006	2006Jun13_258	0.0	1.9	0.0	8.9	28.8	60.4	3
K2, 13 June 2006	2006Jun13_259	0.0	0.0	0.0	2.0	20.3	77.6	3
K2, 13 June 2006	2006Jun13_260	20.7	1.6	32.1	20.0	9.6	16.1	1
K2, 13 June 2006	2006Jun13_261	16.9	2.0	35.3	14.1	8.1	23.6	2
K2, 13 June 2006	2006Jun13_262	16.3	1.3	23.0	14.5	10.6	34.3	2
K2, 13 June 2006	2006Jun13_263	14.5	2.4	18.6	14.4	14.2	36.0	2
K2, 13 June 2006	2006Jun13_264	17.0	2.3	41.3	17.2	7.8	14.5	1
K2, 13 June 2006	2006Jun13_265	18.5	2.3	41.4	17.6	6.2	14.2	1
K2, 13 June 2006	2006Jun13_266	0.0	0.5	5.3	2.3	17.1	74.8	3
K2, 13 June 2006	2006Jun13_267	3.3	0.8	10.3	9.1	15.4	61.0	3
K2, 13 June 2006	2006Jun13_268	21.3	2.0	43.0	18.5	4.9	10.3	1

K2, 13 June 2006	2006Jun13_269	15.4	1.4	4.8	12.5	20.4	45.5	3
K2, 13 June 2006	2006Jun13_270	21.2	2.1	39.8	19.5	5.4	12.0	1
K2, 13 June 2006	2006Jun13_271	21.4	1.9	39.5	19.5	6.0	11.8	1
K2, 13 June 2006	2006Jun13_272	21.7	0.9	30.9	16.4	11.4	18.7	1
K2, 13 June 2006	2006Jun13_273	18.9	1.9	37.2	17.0	8.8	16.3	1
K2, 13 June 2006	2006Jun13_274	19.7	2.0	47.0	17.2	4.9	9.2	1
K2, 13 June 2006	2006Jun13_275	18.0	2.1	28.0	19.8	10.6	21.4	2
K2, 13 June 2006	2006Jun13_276	11.6	1.8	23.8	15.5	11.3	36.1	2
K2, 13 June 2006	2006Jun13_277	3.2	1.4	8.4	10.9	11.5	64.5	3
K2, 13 June 2006	2006Jun13_278	13.4	1.6	14.8	14.2	14.4	41.6	2
K2, 13 June 2006	2006Jun13_279	16.3	2.2	27.2	23.8	9.6	21.0	2
K2, 13 June 2006	2006Jun13_280	10.6	1.0	21.9	10.3	10.6	45.6	2
K2, 13 June 2006	2006Jun13_281	16.4	2.4	21.4	21.3	11.6	26.9	2
K2, 13 June 2006	2006Jun13_282	5.7	1.1	3.7	13.6	20.7	55.2	3
K2, 13 June 2006	2006Jun13_283	18.2	1.7	24.3	18.7	12.3	24.9	2
K2, 13 June 2006	2006Jun13_284	31.0	0.0	1.5	14.0	18.2	35.3	2
K2, 13 June 2006	2006Jun13_285	24.1	1.9	33.2	17.8	8.7	14.3	1
K2, 13 June 2006	2006Jun13_286	15.8	1.6	33.6	14.9	9.1	25.0	2
K2, 13 June 2006	2006Jun13_287	20.3	2.0	34.2	19.5	10.0	14.0	1
K2, 13 June 2006	2006Jun13_288	18.3	2.4	34.9	13.7	8.5	22.2	1
K2, 13 June 2006	2006Jun13_289	21.7	1.9	43.4	19.3	4.6	9.2	1
K2, 13 June 2006	2006Jun13_290	23.1	1.9	46.3	19.1	2.9	6.8	1
K2, 13 June 2006	2006Jun13_291	8.3	0.7	4.9	13.1	21.1	51.8	3
K2, 13 June 2006	2006Jun13_292	17.9	1.6	29.2	15.8	9.6	25.9	2
K2, 13 June 2006	2006Jun13_293	19.2	2.0	43.8	18.9	4.9	11.2	1
K2, 13 June 2006	2006Jun13_294	25.5	1.2	26.6	15.5	9.0	22.1	2
K2, 13 June 2006	2006Jun13_295	23.1	2.1	24.1	16.6	11.7	22.4	2
K2, 13 June 2006	2006Jun13_296	24.8	1.6	42.5	18.1	4.3	8.7	1
K2, 13 June 2006	2006Jun13_297	18.0	1.6	33.1	18.9	11.7	16.7	1
K2, 13 June 2006	2006Jun13_298	1.6	0.5	4.8	5.2	19.3	68.5	3
K2, 13 June 2006	2006Jun13_299	8.6	0.4	6.8	10.1	21.5	52.6	3
K2, 13 June 2006	2006Jun13_300	14.6	1.4	39.6	18.9	10.8	14.8	1
K2, 13 June 2006	2006Jun13_301	22.4	1.4	27.6	22.2	9.0	17.4	1
K2, 13 June 2006	2006Jun13_302	19.6	1.0	30.0	17.4	11.6	20.5	2
K2, 13 June 2006	2006Jun13_303	18.6	1.1	30.5	18.2	11.3	20.3	2
K2, 13 June 2006	2006Jun13_304	6.8	0.5	12.2	9.5	16.4	54.6	3
K2, 13 June 2006	2006Jun13_305	20.6	1.5	37.9	21.9	7.2	10.8	1
K2, 13 June 2006	2006Jun13_306	2.9	0.5	8.2	6.0	17.9	64.4	3
K2, 13 June 2006	2006Jun13_307	14.8	1.3	33.3	17.6	9.9	23.1	2
K2, 13 June 2006	2006Jun13_308	15.5	1.3	30.7	17.9	9.4	25.1	2
K2, 13 June 2006	2006Jun13_309	7.4	0.3	9.2	9.9	18.2	55.0	3
K2, 13 June 2006	2006Jun13_310	11.6	0.1	7.0	12.5	17.8	51.1	3
K2, 13 June 2006	2006Jun13_311	21.6	1.1	27.6	23.6	10.8	15.3	1
K2, 13 June 2006	2006Jun13_312	10.8	0.9	31.1	16.0	10.7	30.5	2
K2, 13 June 2006	2006Jun13_313	22.4	1.4	36.5	23.5	7.6	8.7	1
K2, 13 June 2006	2006Jun13_314	15.2	0.5	12.5	18.8	15.8	37.2	2
K2, 13 June 2006	2006Jun13_315	9.4	0.8	22.9	12.3	13.2	41.3	2
K2, 13 June 2006	2006Jun13_316	18.4	1.8	36.1	19.8	9.1	14.8	1
K2, 13 June 2006	2006Jun13_317	15.5	1.3	29.1	21.2	10.2	22.7	2
K2, 13 June 2006	2006Jun13_318	22.4	1.6	38.7	22.5	5.8	9.0	1
K2, 13 June 2006	2006Jun13_319	14.7	1.6	37.7	18.9	9.6	17.5	1
K2, 13 June 2006	2006Jun13_320	14.5	1.4	13.1	26.0	14.3	30.7	2
K2, 13 June 2006	2006Jun13_321	13.9	1.9	33.6	19.4	7.5	23.7	2
K2, 13 June 2006	2006Jun13_322	20.6	1.7	36.0	21.9	8.0	11.9	1
K2, 13 June 2006	2006Jun13_323	21.2	1.6	10.5	32.4	12.0	22.3	2
K2, 13 June 2006	2006Jun13_324	15.2	1.7	28.1	18.7	9.1	27.1	2
K2, 13 June 2006	2006Jun13_325	0.0	1.5	3.3	8.8	13.4	74.4	3
K2, 13 June 2006	2006Jun13_326	9.8	1.2	29.2	15.3	13.7	30.8	2
K2, 13 June 2006	2006Jun13_327	17.6	1.5	41.3	20.1	7.7	11.8	1
K2, 13 June 2006	2006Jun13_328	28.5	0.7	30.2	17.2	8.5	14.9	1
K2, 13 June 2006	2006Jun13_329	18.3	1.3	42.9	20.6	4.9	12.0	1
K2, 13 June 2006	2006Jun13_330	22.3	1.3	37.2	22.2	6.6	10.5	1
K2, 13 June 2006	2006Jun13_331	3.0	0.6	8.8	5.8	18.3	63.6	3
K2, 13 June 2006	2006Jun13_332	7.2	1.0	12.9	9.7	17.2	52.0	3
K2, 13 June 2006	2006Jun13_333	15.7	0.7	16.2	18.4	16.6	32.4	2
K2, 13 June 2006	2006Jun13_334	16.8	1.4	41.7	21.7	6.7	11.7	1
K2, 13 June 2006	2006Jun13_335	0.5	0.3	2.0	3.5	20.4	73.3	3
K2, 13 June 2006	2006Jun13_336	0.6	0.4	2.9	3.2	20.5	72.3	3
K2, 13 June 2006	2006Jun13_337	17.6	1.4	31.2	19.9	10.9	19.0	2
K2, 13 June 2006	2006Jun13_338	18.4	2.6	37.1	19.6	7.7	14.7	1
K2, 13 June 2006	2006Jun13_339	20.8	0.5	31.7	23.5	9.6	13.9	1
K2, 13 June 2006	2006Jun13_340	16.8	1.3	21.7	17.4	15.8	26.9	2
K2, 13 June 2006	2006Jun13_341	28.1	1.5	28.5	20.8	8.8	12.3	1
K2, 13 June 2006	2006Jun13_342	15.3	2.2	34.4	24.3	9.3	14.6	1
K2, 13 June 2006	2006Jun13_343	13.4	2.2	30.5	22.9	8.0	23.0	2
K2, 13 June 2006	2006Jun13_344	18.1	2.1	31.6	22.3	8.9	17.0	1
K2, 13 June 2006	2006Jun13_345	14.1	2.0	34.9	18.3	10.8	20.0	1
K2, 13 June 2006	2006Jun13_346	19.3	1.5	39.8	19.8	6.7	12.9	1
K2, 13 June 2006	2006Jun13_347	4.0	0.2	6.4	5.2	19.7	64.5	3
K2, 13 June 2006	2006Jun13_348	16.9	1.3	40.2	20.8	8.2	12.6	1
K2, 13 June 2006	2006Jun13_349	17.2	1.1	36.8	18.6	10.9	15.4	1
K2, 13 June 2006	2006Jun13_350	3.5	0.1	2.3	4.9	19.7	69.5	3
K2, 13 June 2006	2006Jun13_351	13.9	0.9	28.6	12.9	11.7	32.0	2
K2, 13 June 2006	2006Jun13_352	19.9	1.6	33.8	20.6	9.4	14.7	1
K2, 13 June 2006	2006Jun13_353	21.4	1.7	41.7	20.2	6.3	8.8	1
K2, 13 June 2006	2006Jun13_354	21.9	1.7	24.4	19.2	12.3	20.6	2

K2, 13 June 2006	2006Jun13_355	2.4	0.6	5.9	5.3	19.9	66.0	3
K2, 13 June 2006	2006Jun13_356	18.7	2.3	18.6	18.5	17.0	25.0	2
K2, 13 June 2006	2006Jun13_357	8.7	1.3	19.8	10.5	16.7	43.0	2
K2, 13 June 2006	2006Jun13_358	4.4	0.7	10.9	6.7	19.4	58.0	3
K2, 13 June 2006	2006Jun13_359	19.0	1.9	43.6	17.6	6.7	11.4	1
K2, 13 June 2006	2006Jun13_360	11.0	1.2	25.0	13.1	13.3	36.4	2
K2, 13 June 2006	2006Jun13_361	1.4	0.4	5.2	3.6	18.1	71.3	3
K2, 13 June 2006	2006Jun13_362	10.2	1.2	24.8	11.6	13.5	38.6	2
K2, 13 June 2006	2006Jun13_363	5.1	0.8	13.4	7.9	16.7	56.1	3
K2, 13 June 2006	2006Jun13_364	3.1	0.7	9.0	5.3	19.0	62.8	3
K2, 13 June 2006	2006Jun13_365	14.7	2.0	37.7	16.9	9.6	19.2	1
K2, 13 June 2006	2006Jun13_366	22.6	1.6	20.3	20.3	13.9	21.2	2
K2, 13 June 2006	2006Jun13_367	21.1	1.5	34.7	21.3	8.9	12.6	1
K2, 13 June 2006	2006Jun13_368	22.5	1.3	34.4	20.3	8.7	12.8	1
K2, 13 June 2006	2006Jun13_369	19.2	2.0	32.5	20.0	9.0	17.4	1
K2, 13 June 2006	2006Jun13_370	17.2	1.2	35.7	17.0	9.1	19.8	1
K2, 13 June 2006	2006Jun13_371	22.5	1.8	35.2	21.7	8.2	10.6	1
K2, 13 June 2006	2006Jun13_372	10.5	1.0	24.2	11.8	13.9	38.6	2
K2, 13 June 2006	2006Jun13_373	0.7	0.0	0.0	7.9	35.0	56.4	3
K2, 13 June 2006	2006Jun13_374	18.6	1.7	40.4	19.8	7.9	11.6	1
K2, 13 June 2006	2006Jun13_375	21.5	1.2	35.5	20.1	8.0	13.6	1
K2, 13 June 2006	2006Jun13_376	20.6	1.4	42.0	20.3	6.2	9.5	1
K2, 13 June 2006	2006Jun13_377	18.9	1.8	40.2	18.2	8.5	12.4	1
K2, 13 June 2006	2006Jun13_378	18.9	1.2	26.1	17.8	14.0	21.9	2
K2, 13 June 2006	2006Jun13_379	22.4	1.6	37.7	20.0	8.1	10.2	1
K2, 13 June 2006	2006Jun13_380	1.3	0.4	3.4	4.1	20.3	70.5	3
K2, 13 June 2006	2006Jun13_381	2.6	1.0	0.0	11.8	33.1	51.5	3
K2, 13 June 2006	2006Jun13_382	24.2	1.5	24.4	21.1	11.8	17.1	2
K2, 13 June 2006	2006Jun13_383	3.6	0.4	0.0	11.2	33.6	51.2	3
K2, 13 June 2006	2006Jun13_384	20.1	1.9	11.3	19.3	19.0	28.3	2
K2, 13 June 2006	2006Jun13_385	26.4	1.8	36.3	19.5	6.7	9.2	1
K2, 13 June 2006	2006Jun13_386	18.7	0.8	9.4	21.3	19.8	30.0	2
K2, 13 June 2006	2006Jun13_387	20.0	1.9	44.6	17.7	7.1	8.7	1
K2, 13 June 2006	2006Jun13_388	21.2	1.2	33.5	19.6	10.7	13.8	1
K2, 13 June 2006	2006Jun13_389	2.4	0.6	7.6	4.5	19.4	65.4	3
K2, 13 June 2006	2006Jun13_390	2.7	0.6	7.5	4.6	19.5	65.1	3
K2, 13 June 2006	2006Jun13_391	19.6	1.9	38.8	18.9	7.9	12.9	1
K2, 13 June 2006	2006Jun13_392	5.5	0.6	18.2	9.4	15.3	50.9	3
K2, 13 June 2006	2006Jun13_393	18.9	1.4	20.5	16.8	15.3	27.2	2
K2, 13 June 2006	2006Jun13_394	21.3	1.6	41.9	19.5	6.8	9.0	1
K2, 13 June 2006	2006Jun13_395	15.5	1.7	35.8	15.5	9.4	22.1	1
K2, 13 June 2006	2006Jun13_396	20.3	1.5	38.3	17.4	9.0	13.6	1
K2, 13 June 2006	2006Jun13_397	25.3	1.0	28.0	14.7	12.4	18.7	2
K2, 13 June 2006	2006Jun13_398	17.2	1.1	41.7	20.0	6.8	13.2	1
K2, 13 June 2006	2006Jun13_399	25.0	2.0	38.4	17.3	7.1	10.2	1
K2, 13 June 2006	2006Jun13_400	24.7	1.7	36.0	19.5	6.8	11.2	1
K2, 13 June 2006	2006Jun13_401	26.2	1.7	21.0	19.0	12.0	20.0	2
K2, 13 June 2006	2006Jun13_402	18.8	1.6	36.0	18.7	10.5	14.4	1
K2, 13 June 2006	2006Jun13_403	20.5	0.4	21.8	19.0	11.2	27.1	2
K2, 13 June 2006	2006Jun13_404	20.5	0.9	34.3	21.6	8.6	14.2	1
K2, 13 June 2006	2006Jun13_405	20.1	1.8	37.2	18.5	10.1	12.3	1
K2, 13 June 2006	2006Jun13_406	19.2	1.4	39.0	19.1	6.9	14.5	1
K2, 13 June 2006	2006Jun13_407	13.4	1.3	17.4	15.2	16.5	36.2	2
K2, 13 June 2006	2006Jun13_408	12.1	2.4	17.7	17.3	15.8	34.8	2
K2, 13 June 2006	2006Jun13_409	13.5	1.4	39.1	17.7	8.8	19.6	1
K2, 13 June 2006	2006Jun13_410	19.6	1.0	19.5	16.6	16.1	27.3	2
K2, 13 June 2006	2006Jun13_411	23.0	1.6	37.3	17.0	9.7	11.4	1
K2, 13 June 2006	2006Jun13_412	11.7	1.1	30.9	14.9	11.8	29.6	2
K2, 13 June 2006	2006Jun13_413	8.9	0.7	17.4	10.2	15.7	47.0	3
K2, 13 June 2006	2006Jun13_414	17.6	1.8	44.7	18.8	7.0	10.0	1
K2, 13 June 2006	2006Jun13_415	4.5	0.9	12.4	7.2	20.1	54.9	3
K2, 13 June 2006	2006Jun13_416	18.4	1.5	38.3	18.6	9.1	14.1	1
K2, 13 June 2006	2006Jun13_417	21.2	1.3	27.7	17.7	12.6	19.6	2
K2, 13 June 2006	2006Jun13_418	12.0	0.8	20.8	12.5	14.6	39.3	2
K2, 13 June 2006	2006Jun13_419	14.9	1.2	30.0	16.7	12.0	25.2	2
K2, 30 June 2006	2006Jun30_1	4.6	1.8	9.1	8.1	23.6	52.8	3
K2, 30 June 2006	2006Jun30_2	22.8	2.3	24.0	24.7	10.6	15.6	2
K2, 30 June 2006	2006Jun30_3	0.1	1.2	7.7	1.8	8.2	81.0	3
K2, 30 June 2006	2006Jun30_4	21.3	3.2	38.6	13.0	9.2	14.7	1
K2, 30 June 2006	2006Jun30_5	19.1	3.2	37.7	11.8	13.3	15.0	1
K2, 30 June 2006	2006Jun30_6	10.5	1.9	20.0	8.6	22.2	36.7	2
K2, 30 June 2006	2006Jun30_7	14.3	3.3	36.1	11.4	16.2	18.7	1
K2, 30 June 2006	2006Jun30_8	21.8	2.6	31.1	14.2	10.8	19.6	1
K2, 30 June 2006	2006Jun30_9	17.0	3.5	42.8	6.4	11.6	18.6	1
K2, 30 June 2006	2006Jun30_10	15.4	3.1	27.7	11.9	16.3	25.5	2
K2, 30 June 2006	2006Jun30_11	5.0	0.3	0.0	12.0	41.0	41.7	3
K2, 30 June 2006	2006Jun30_12	18.8	2.4	36.3	14.6	10.5	17.4	1
K2, 30 June 2006	2006Jun30_13	17.8	3.5	41.1	13.6	9.9	14.1	1
K2, 30 June 2006	2006Jun30_14	13.2	2.9	21.9	10.5	16.5	35.0	2
K2, 30 June 2006	2006Jun30_15	16.5	4.8	40.9	10.6	8.8	18.3	1
K2, 30 June 2006	2006Jun30_16	4.2	1.4	13.1	5.1	20.3	55.9	3
K2, 30 June 2006	2006Jun30_17	20.4	2.3	34.2	13.2	12.3	17.7	1
K2, 30 June 2006	2006Jun30_18	13.1	2.8	36.4	14.9	13.6	19.3	1
K2, 30 June 2006	2006Jun30_19	5.3	1.8	11.3	10.1	25.5	46.1	3
K2, 30 June 2006	2006Jun30_20	22.7	2.9	37.3	13.9	10.6	12.6	1
K2, 30 June 2006	2006Jun30_21	22.3	2.1	37.0	12.9	10.4	15.2	1

K2, 30 June 2006	2006Jun30_22	21.1	2.4	28.7	12.5	14.9	20.3	2
K2, 30 June 2006	2006Jun30_23	18.0	2.9	43.8	14.0	8.4	12.9	1
K2, 30 June 2006	2006Jun30_24	0.1	0.2	0.3	2.1	23.1	74.3	3
K2, 30 June 2006	2006Jun30_25	20.3	2.8	41.2	12.5	9.7	13.5	1
K2, 30 June 2006	2006Jun30_26	23.7	3.4	37.8	13.1	9.5	12.5	1
K2, 30 June 2006	2006Jun30_27	5.6	1.4	15.3	5.8	20.3	51.6	3
K2, 30 June 2006	2006Jun30_28	13.2	2.2	28.8	10.0	15.9	29.9	2
K2, 30 June 2006	2006Jun30_29	9.3	2.2	24.8	8.1	15.7	39.9	2
K2, 30 June 2006	2006Jun30_30	24.6	3.5	45.7	11.8	5.7	8.7	1
K2, 30 June 2006	2006Jun30_31	15.4	2.2	23.6	11.1	17.5	30.2	2
K2, 30 June 2006	2006Jun30_32	29.1	4.0	31.3	14.3	9.1	12.3	1
K2, 30 June 2006	2006Jun30_33	1.1	0.9	5.0	3.0	23.9	66.1	3
K2, 30 June 2006	2006Jun30_34	22.1	3.1	39.8	12.9	9.3	12.9	1
K2, 30 June 2006	2006Jun30_35	20.5	2.9	39.4	13.0	10.5	13.7	1
K2, 30 June 2006	2006Jun30_36	17.0	3.1	42.0	14.2	10.1	13.7	1
K2, 30 June 2006	2006Jun30_37	5.6	1.1	9.2	4.8	20.2	59.1	3
K2, 30 June 2006	2006Jun30_38	0.0	0.0	0.0	3.6	24.4	71.9	3
K2, 30 June 2006	2006Jun30_39	17.9	3.3	29.1	11.7	14.1	23.9	2
K2, 30 June 2006	2006Jun30_40	21.0	2.7	43.9	13.6	6.6	12.3	1
K2, 30 June 2006	2006Jun30_41	0.0	0.6	0.0	7.2	37.2	55.0	3
K2, 30 June 2006	2006Jun30_42	35.4	0.0	27.1	12.3	11.1	14.3	1
K2, 30 June 2006	2006Jun30_43	22.0	3.1	35.1	12.6	11.4	15.7	1
K2, 30 June 2006	2006Jun30_44	17.0	3.2	31.8	10.4	12.3	25.3	2
K2, 30 June 2006	2006Jun30_45	10.3	2.1	18.7	10.0	20.2	38.7	2
K2, 30 June 2006	2006Jun30_46	11.2	3.5	22.7	10.0	16.9	35.7	2
K2, 30 June 2006	2006Jun30_47	19.0	3.0	37.5	12.3	11.2	17.0	1
K2, 30 June 2006	2006Jun30_48	20.6	3.7	45.0	11.0	6.0	13.7	1
K2, 30 June 2006	2006Jun30_49	22.9	2.4	22.6	9.2	15.0	27.9	2
K2, 30 June 2006	2006Jun30_50	17.4	3.1	34.2	12.5	14.4	18.5	1
K2, 30 June 2006	2006Jun30_51	13.5	2.5	27.9	12.2	18.6	25.3	2
K2, 30 June 2006	2006Jun30_52	17.4	3.1	37.6	13.1	12.8	16.0	1
K2, 30 June 2006	2006Jun30_53	19.7	2.6	24.3	13.1	15.6	24.8	2
K2, 30 June 2006	2006Jun30_54	16.2	2.7	34.3	11.8	14.6	20.5	2
K2, 30 June 2006	2006Jun30_55	19.0	3.1	29.7	14.2	15.7	18.3	2
K2, 30 June 2006	2006Jun30_56	25.8	3.5	34.1	14.8	10.4	11.4	1
K2, 30 June 2006	2006Jun30_57	0.0	0.3	0.0	5.8	32.9	61.0	3
K2, 30 June 2006	2006Jun30_58	16.3	2.4	8.2	13.0	18.9	41.3	2
K2, 30 June 2006	2006Jun30_59	18.6	3.2	29.6	11.8	12.7	24.1	2
K2, 30 June 2006	2006Jun30_60	3.9	0.6	8.7	5.1	22.9	58.7	3
K2, 30 June 2006	2006Jun30_61	7.5	2.0	17.2	5.7	19.3	48.3	3
K2, 30 June 2006	2006Jun30_62	21.2	3.2	43.7	12.8	8.4	10.7	1
K2, 30 June 2006	2006Jun30_63	20.3	3.9	35.2	13.9	11.8	14.8	1
K2, 30 June 2006	2006Jun30_64	21.4	3.5	34.1	13.4	12.3	15.4	1
K2, 30 June 2006	2006Jun30_65	14.5	2.1	16.9	14.7	17.9	33.9	2
K2, 30 June 2006	2006Jun30_66	13.0	1.6	31.7	13.9	9.5	30.4	2
K2, 30 June 2006	2006Jun30_67	21.1	2.2	36.0	16.4	7.6	16.7	1
K2, 30 June 2006	2006Jun30_68	23.2	4.0	21.6	13.7	12.2	25.2	2
K2, 30 June 2006	2006Jun30_69	19.3	1.9	45.3	18.0	4.9	10.6	1
K2, 30 June 2006	2006Jun30_70	13.7	2.3	38.5	16.3	10.1	19.1	1
K2, 30 June 2006	2006Jun30_71	16.1	1.9	40.4	16.9	7.7	17.0	1
K2, 30 June 2006	2006Jun30_72	21.3	2.7	42.7	16.8	5.7	10.9	1
K2, 30 June 2006	2006Jun30_73	19.3	2.1	40.4	16.3	7.6	14.3	1
K2, 30 June 2006	2006Jun30_74	17.8	2.2	40.7	17.5	8.7	13.1	1
K2, 30 June 2006	2006Jun30_75	20.2	2.0	40.7	17.7	7.1	12.4	1
K2, 30 June 2006	2006Jun30_76	17.3	2.2	41.0	15.0	7.5	17.0	1
K2, 30 June 2006	2006Jun30_77	16.2	1.8	32.7	11.2	9.6	28.5	2
K2, 30 June 2006	2006Jun30_78	19.8	1.4	39.4	20.9	6.4	12.1	1
K2, 30 June 2006	2006Jun30_79	14.8	1.9	34.1	17.9	10.4	21.0	2
K2, 30 June 2006	2006Jun30_80	20.8	2.2	43.6	17.0	6.5	9.8	1
K2, 30 June 2006	2006Jun30_81	18.7	2.3	44.2	17.5	6.9	10.3	1
K2, 30 June 2006	2006Jun30_82	14.8	2.3	39.6	16.8	9.3	17.2	1
K2, 30 June 2006	2006Jun30_83	19.1	2.1	46.6	17.2	5.3	9.6	1
K2, 30 June 2006	2006Jun30_84	14.5	2.1	16.9	14.7	17.9	33.9	2
K2, 30 June 2006	2006Jun30_85	14.5	2.1	16.9	14.7	17.9	33.9	2
K2, 30 June 2006	2006Jun30_86	20.4	2.5	41.2	12.8	7.7	15.3	1
K2, 30 June 2006	2006Jun30_87	8.9	1.6	19.5	6.7	17.3	46.0	3
K2, 30 June 2006	2006Jun30_88	24.1	2.2	43.6	13.4	5.0	11.7	1
K2, 30 June 2006	2006Jun30_89	31.6	2.1	29.3	17.4	8.1	11.6	1
K2, 30 June 2006	2006Jun30_90	29.5	2.6	39.9	14.3	5.2	8.6	1
K2, 30 June 2006	2006Jun30_91	24.2	2.5	44.8	15.4	5.1	8.0	1
K2, 30 June 2006	2006Jun30_92	25.7	2.2	43.2	14.4	4.9	9.6	1
K2, 30 June 2006	2006Jun30_93	20.8	2.2	45.9	14.4	4.9	11.8	1
K2, 30 June 2006	2006Jun30_94	2.1	0.8	7.0	4.0	20.1	66.1	3
K2, 30 June 2006	2006Jun30_95	1.5	0.7	5.7	3.3	20.5	68.3	3
K2, 30 June 2006	2006Jun30_96	24.3	2.2	41.1	14.7	6.1	11.5	1
K2, 30 June 2006	2006Jun30_97	20.4	2.2	47.2	18.3	3.8	8.0	1
K2, 30 June 2006	2006Jun30_98	15.4	2.1	19.8	14.6	11.4	36.7	2
K2, 30 June 2006	2006Jun30_99	15.3	1.7	28.0	12.0	11.4	31.6	2
K2, 30 June 2006	2006Jun30_100	22.1	1.3	38.0	17.3	7.5	13.8	1
K2, 30 June 2006	2006Jun30_101	21.5	1.5	35.7	17.8	9.5	14.0	1
K2, 30 June 2006	2006Jun30_102	14.6	1.3	31.8	13.0	10.4	28.9	2
K2, 30 June 2006	2006Jun30_103	16.7	1.4	31.1	11.8	9.7	29.3	2
K2, 30 June 2006	2006Jun30_104	22.0	3.0	24.5	13.1	12.8	24.7	2
K2, 30 June 2006	2006Jun30_105	18.4	2.1	48.6	18.0	4.0	8.8	1
K2, 30 June 2006	2006Jun30_106	12.7	1.3	26.9	11.8	11.2	36.2	2
K2, 30 June 2006	2006Jun30_107	22.4	2.0	45.1	17.9	3.7	8.9	1

K2, 30 June 2006	2006Jun30_108	25.4	3.0	41.8	14.6	6.0	9.3	1
K2, 30 June 2006	2006Jun30_109	16.9	2.0	36.6	15.2	8.4	20.9	1
K2, 30 June 2006	2006Jun30_110	0.0	1.5	0.0	10.7	36.2	51.7	3
K2, 30 June 2006	2006Jun30_111	11.6	1.8	24.9	11.2	12.7	37.9	2
K2, 30 June 2006	2006Jun30_112	15.6	1.9	26.0	11.3	11.6	33.6	2
K2, 30 June 2006	2006Jun30_113	25.9	1.6	45.7	16.6	4.0	6.3	1
K2, 30 June 2006	2006Jun30_114	24.8	2.4	41.1	18.1	5.6	8.0	1
K2, 30 June 2006	2006Jun30_115	35.3	1.0	34.8	17.2	4.3	7.5	1
K2, 30 June 2006	2006Jun30_116	22.4	1.3	16.3	13.4	15.0	31.5	2
K2, 30 June 2006	2006Jun30_117	25.6	3.1	31.7	15.8	7.9	15.9	1
K2, 30 June 2006	2006Jun30_118	20.1	2.5	36.6	15.4	9.1	16.3	1
K2, 30 June 2006	2006Jun30_119	26.1	2.9	39.3	14.5	6.2	10.9	1
K2, 30 June 2006	2006Jun30_120	24.5	2.3	45.1	15.9	4.6	7.5	1
K2, 30 June 2006	2006Jun30_121	25.2	1.6	41.3	19.1	5.0	7.8	1
K2, 30 June 2006	2006Jun30_122	23.2	2.6	45.5	14.6	6.2	7.9	1
K2, 30 June 2006	2006Jun30_123	16.6	1.9	32.9	13.8	11.8	22.9	2
K2, 30 June 2006	2006Jun30_124	20.3	2.3	28.2	17.3	12.1	19.9	2
K2, 30 June 2006	2006Jun30_125	31.8	3.0	30.8	16.5	7.8	10.1	1
K2, 30 June 2006	2006Jun30_126	14.2	1.5	25.1	11.8	12.5	34.9	2
K2, 30 June 2006	2006Jun30_127	22.0	2.5	44.1	14.6	5.7	11.1	1
K2, 30 June 2006	2006Jun30_128	9.3	1.3	22.0	8.5	13.6	45.3	2
K2, 30 June 2006	2006Jun30_129	22.9	2.4	45.6	17.4	4.8	6.9	1
K2, 30 June 2006	2006Jun30_130	26.0	1.9	34.2	20.4	7.1	10.4	1
K2, 30 June 2006	2006Jun30_131	3.4	0.9	10.1	4.4	18.1	63.1	3
K2, 30 June 2006	2006Jun30_132	21.1	2.4	25.9	18.2	11.9	20.5	2
K2, 30 June 2006	2006Jun30_133	21.8	1.9	51.2	18.6	2.8	3.6	1
K2, 30 June 2006	2006Jun30_134	23.7	2.4	38.7	14.9	8.3	12.0	1
K2, 30 June 2006	2006Jun30_135	9.2	1.6	12.2	12.3	24.0	40.7	3
K2, 30 June 2006	2006Jun30_136	0.1	0.2	0.0	4.9	27.9	66.9	3
K2, 30 June 2006	2006Jun30_137	25.2	2.5	34.2	15.8	8.6	13.8	1
K2, 30 June 2006	2006Jun30_138	19.0	1.9	42.2	14.5	6.7	15.7	1
K2, 30 June 2006	2006Jun30_139	20.4	2.4	44.9	15.7	7.1	9.6	1
K2, 30 June 2006	2006Jun30_140	25.0	2.6	35.0	14.3	10.9	12.2	1
K2, 30 June 2006	2006Jun30_141	22.6	2.6	47.3	14.9	5.6	7.0	1
K2, 30 June 2006	2006Jun30_142	17.9	1.8	27.4	11.9	11.5	29.5	2
K2, 30 June 2006	2006Jun30_143	18.5	1.6	25.7	13.3	11.8	29.0	2
K2, 30 June 2006	2006Jun30_144	19.8	2.5	44.8	18.1	5.1	9.7	1
K2, 30 June 2006	2006Jun30_145	28.1	2.0	32.1	17.6	7.9	12.2	1
K2, 30 June 2006	2006Jun30_146	20.4	2.2	33.4	12.9	14.0	17.1	1
K2, 30 June 2006	2006Jun30_147	15.4	2.2	8.5	13.2	28.2	32.5	2
K2, 30 June 2006	2006Jun30_148	4.9	1.2	10.5	5.6	22.7	55.3	3
K2, 30 June 2006	2006Jun30_149	13.9	1.3	19.4	8.2	19.1	38.1	2
K2, 30 June 2006	2006Jun30_150	22.3	2.6	43.6	14.2	8.1	9.2	1
K2, 30 June 2006	2006Jun30_151	15.6	2.8	20.6	12.7	20.1	28.1	2
K2, 30 June 2006	2006Jun30_152	17.6	3.4	44.5	13.7	8.9	11.9	1
K2, 30 June 2006	2006Jun30_153	2.7	0.9	7.7	4.7	24.1	59.9	3
K2, 30 June 2006	2006Jun30_154	19.9	2.6	37.4	17.6	11.4	11.0	1
K2, 30 June 2006	2006Jun30_155	10.9	1.9	23.6	10.3	18.4	35.0	2
K2, 30 June 2006	2006Jun30_156	22.9	3.5	42.3	15.1	8.6	7.7	1
K2, 30 June 2006	2006Jun30_157	16.8	3.6	44.2	13.3	7.6	14.5	1
K2, 30 June 2006	2006Jun30_158	17.8	3.0	43.2	13.9	9.7	12.4	1
K2, 30 June 2006	2006Jun30_159	23.3	4.8	47.1	13.4	3.1	8.2	1
K2, 30 June 2006	2006Jun30_160	3.9	1.4	12.6	5.4	22.9	53.8	3
K2, 30 June 2006	2006Jun30_161	10.4	2.5	15.2	9.5	21.8	40.6	2
K2, 30 June 2006	2006Jun30_162	17.2	3.4	16.8	16.7	22.4	23.5	2
K2, 30 June 2006	2006Jun30_163	13.8	2.8	28.0	14.9	17.6	22.8	2
K2, 30 June 2006	2006Jun30_164	18.4	2.8	30.3	18.0	13.0	17.5	2
K2, 30 June 2006	2006Jun30_165	17.5	3.1	39.3	12.5	12.6	15.1	1
K2, 30 June 2006	2006Jun30_166	22.1	4.0	49.0	13.0	5.1	7.0	1
K2, 30 June 2006	2006Jun30_167	20.0	3.6	43.2	12.2	9.0	11.9	1
K2, 30 June 2006	2006Jun30_168	9.9	3.8	24.4	16.5	19.4	25.9	2
K2, 30 June 2006	2006Jun30_169	20.4	3.1	47.0	13.1	7.6	8.8	1
K2, 30 June 2006	2006Jun30_170	20.1	3.1	45.1	13.3	7.3	11.1	1
K2, 30 June 2006	2006Jun30_171	0.0	0.0	0.0	8.6	44.1	51.2	3
K2, 30 June 2006	2006Jun30_172	6.5	2.7	15.8	9.6	23.4	42.0	3
K2, 30 June 2006	2006Jun30_173	20.0	2.9	45.3	12.9	6.8	12.1	1
K2, 30 June 2006	2006Jun30_174	17.1	3.1	42.7	13.1	10.4	13.5	1
K2, 30 June 2006	2006Jun30_175	16.2	2.8	40.9	13.3	11.7	15.1	1
K2, 30 June 2006	2006Jun30_176	12.2	2.3	24.9	16.3	22.5	21.9	2
K2, 30 June 2006	2006Jun30_177	22.4	3.1	46.4	14.9	6.9	6.4	1
K2, 30 June 2006	2006Jun30_178	9.3	2.3	24.1	9.2	16.9	38.1	2
K2, 30 June 2006	2006Jun30_179	15.9	3.1	29.4	12.8	15.4	23.4	2
K2, 30 June 2006	2006Jun30_180	14.0	2.8	18.4	20.6	23.1	21.2	2
K2, 30 June 2006	2006Jun30_181	15.9	1.8	27.8	10.3	14.5	29.7	2
K2, 30 June 2006	2006Jun30_182	22.3	3.2	47.1	13.3	6.6	7.5	1
K2, 30 June 2006	2006Jun30_183	20.4	3.1	44.6	12.8	8.6	10.4	1
K2, 30 June 2006	2006Jun30_184	22.3	2.7	48.8	13.5	6.3	6.3	1
K2, 30 June 2006	2006Jun30_185	19.8	3.2	44.9	12.5	8.5	11.0	1
K2, 30 June 2006	2006Jun30_186	19.3	3.7	39.6	14.4	10.3	12.7	1
K2, 30 June 2006	2006Jun30_187	22.5	2.8	45.6	13.5	6.8	8.8	1
K2, 30 June 2006	2006Jun30_188	21.4	3.2	27.6	14.4	10.8	22.6	2
K2, 30 June 2006	2006Jun30_189	27.1	2.9	29.6	18.3	8.1	13.9	1
K2, 30 June 2006	2006Jun30_190	24.0	3.1	42.1	13.7	6.7	10.4	1
K2, 30 June 2006	2006Jun30_191	22.5	2.7	33.7	16.3	9.3	15.6	1
K2, 30 June 2006	2006Jun30_192	22.3	1.2	19.4	14.5	16.2	26.3	2
K2, 30 June 2006	2006Jun30_193	20.0	1.8	39.9	22.2	6.8	9.2	1

K2, 30 June 2006	2006Jun30_194	31.2	2.3	40.4	16.0	4.0	6.1	1
K2, 30 June 2006	2006Jun30_195	7.4	1.4	22.8	9.7	15.8	42.9	2
K2, 30 June 2006	2006Jun30_196	22.9	2.2	43.3	16.4	6.9	8.3	1
K2, 30 June 2006	2006Jun30_197	9.6	1.4	20.0	8.4	16.2	44.3	2
K2, 30 June 2006	2006Jun30_198	28.1	2.6	38.5	19.0	5.3	6.4	1
K2, 30 June 2006	2006Jun30_199	27.3	3.0	29.8	16.6	8.3	14.9	1
K2, 30 June 2006	2006Jun30_200	21.8	2.4	45.4	16.5	5.1	8.8	1
K2, 30 June 2006	2006Jun30_201	15.8	2.5	23.0	18.1	12.6	28.0	2
K2, 30 June 2006	2006Jun30_202	0.7	1.7	0.0	10.2	33.6	53.7	3
K2, 30 June 2006	2006Jun30_203	20.9	2.4	47.1	16.1	4.7	8.8	1
K2, 30 June 2006	2006Jun30_204	18.7	1.7	20.8	11.7	12.3	34.8	2
K2, 30 June 2006	2006Jun30_205	7.0	1.0	17.1	6.3	16.9	51.6	3
K2, 30 June 2006	2006Jun30_206	6.2	1.1	14.9	9.7	14.5	53.5	3
K2, 30 June 2006	2006Jun30_207	25.3	2.2	38.6	15.7	6.9	11.2	1
K2, 30 June 2006	2006Jun30_208	24.8	2.2	46.9	16.2	4.0	5.8	1
K2, 30 June 2006	2006Jun30_209	25.6	2.4	40.5	16.6	5.1	9.8	1
K2, 30 June 2006	2006Jun30_210	17.9	2.1	42.9	15.0	7.1	14.9	1
K2, 30 June 2006	2006Jun30_211	24.6	2.3	31.9	18.7	9.8	12.8	1
K2, 30 June 2006	2006Jun30_212	12.1	1.6	35.3	13.3	9.3	28.4	2
K2, 30 June 2006	2006Jun30_213	16.5	1.6	37.3	14.3	6.6	23.7	1
K2, 30 June 2006	2006Jun30_214	16.6	3.0	33.0	17.1	17.5	12.8	1
K2, 30 June 2006	2006Jun30_215	22.9	2.4	26.6	18.8	8.8	20.4	2
K2, 30 June 2006	2006Jun30_216	17.8	1.9	39.3	16.4	6.1	18.5	1
K2, 30 June 2006	2006Jun30_217	23.0	2.5	46.4	15.8	4.4	7.8	1
K2, 30 June 2006	2006Jun30_218	17.6	1.8	21.6	12.3	12.9	33.9	2
K2, 30 June 2006	2006Jun30_219	21.9	2.9	34.6	11.0	11.9	17.8	1
K2, 30 June 2006	2006Jun30_220	8.0	1.9	19.7	11.5	18.5	40.3	2
K2, 30 June 2006	2006Jun30_221	23.7	3.1	38.8	16.6	6.8	10.9	1
K2, 30 June 2006	2006Jun30_222	17.0	2.1	29.2	13.1	11.2	27.5	2
K2, 30 June 2006	2006Jun30_223	24.5	3.0	25.0	17.6	10.6	19.3	2
K2, 30 June 2006	2006Jun30_224	26.0	2.3	42.6	17.1	4.6	7.4	1
K2, 30 June 2006	2006Jun30_225	16.9	4.1	52.6	13.2	0.0	14.7	1
K2, 30 June 2006	2006Jun30_226	17.1	3.9	51.6	13.2	0.0	14.3	1
K2, 9 July 2006	2006Jul_1	17.1	1.7	41.0	17.7	7.5	15.0	1
K2, 9 July 2006	2006Jul_2	13.5	2.0	29.9	13.3	12.5	28.6	2
K2, 9 July 2006	2006Jul_3	7.3	1.3	12.5	10.5	15.3	53.1	3
K2, 9 July 2006	2006Jul_4	17.8	2.5	44.4	15.3	7.1	12.9	1
K2, 9 July 2006	2006Jul_5	20.2	2.1	49.2	16.9	4.7	6.9	1
K2, 9 July 2006	2006Jul_6	21.0	2.4	44.3	17.4	6.2	8.7	1
K2, 9 July 2006	2006Jul_7	20.5	1.9	26.9	14.5	13.5	22.6	2
K2, 9 July 2006	2006Jul_8	18.8	1.9	30.0	13.9	12.4	23.0	2
K2, 9 July 2006	2006Jul_9	16.5	2.3	34.6	13.7	13.4	19.6	1
K2, 9 July 2006	2006Jul_10	22.5	1.3	27.1	14.6	12.1	22.4	2
K2, 9 July 2006	2006Jul_11	23.8	1.9	37.5	15.5	8.2	13.2	1
K2, 9 July 2006	2006Jul_12	17.4	2.2	37.9	15.0	9.2	18.3	1
K2, 9 July 2006	2006Jul_13	10.7	1.8	19.3	11.7	17.5	39.0	2
K2, 9 July 2006	2006Jul_14	17.5	2.4	40.9	16.0	7.2	16.1	1
K2, 9 July 2006	2006Jul_15	3.9	0.9	12.7	5.3	18.3	58.9	3
K2, 9 July 2006	2006Jul_16	15.5	2.4	33.3	17.2	14.7	16.8	1
K2, 9 July 2006	2006Jul_17	18.8	2.1	39.8	15.1	8.7	15.4	1
K2, 9 July 2006	2006Jul_18	15.7	2.8	41.7	14.1	8.6	17.2	1
K2, 9 July 2006	2006Jul_19	10.1	1.6	19.2	12.1	19.4	37.5	2
K2, 9 July 2006	2006Jul_20	19.6	2.5	45.7	17.3	6.3	8.5	1
K2, 9 July 2006	2006Jul_21	20.0	2.4	46.8	19.0	4.5	7.3	1
K2, 9 July 2006	2006Jul_22	15.9	2.5	38.6	15.5	12.8	14.8	1
K2, 9 July 2006	2006Jul_23	17.4	2.4	45.3	18.3	7.5	9.1	1
K2, 9 July 2006	2006Jul_24	18.0	2.2	37.9	15.5	9.5	16.9	1
K2, 9 July 2006	2006Jul_25	25.1	2.1	19.7	15.2	19.8	18.0	2
K2, 9 July 2006	2006Jul_26	3.7	0.9	11.1	5.7	20.4	58.2	3
K2, 9 July 2006	2006Jul_27	9.2	1.6	15.3	12.3	22.6	39.1	2
K2, 9 July 2006	2006Jul_28	7.5	0.6	12.8	7.7	18.7	52.7	3
K2, 9 July 2006	2006Jul_29	19.1	1.8	42.8	19.2	7.8	9.4	1
K2, 9 July 2006	2006Jul_30	1.6	0.5	3.4	4.6	24.9	65.0	3
K2, 9 July 2006	2006Jul_31	13.9	2.0	29.7	11.8	13.2	29.4	2
K2, 9 July 2006	2006Jul_32	17.7	2.0	33.1	16.1	9.2	21.9	2
K2, 9 July 2006	2006Jul_33	21.4	2.3	46.6	17.2	4.4	8.0	1
K2, 9 July 2006	2006Jul_34	24.0	1.1	16.9	14.1	16.7	27.2	2
K2, 9 July 2006	2006Jul_35	18.6	2.4	43.7	15.9	7.2	12.3	1
K2, 9 July 2006	2006Jul_36	16.8	2.1	38.7	16.7	8.6	17.1	1
K2, 9 July 2006	2006Jul_37	19.8	2.6	43.2	16.5	7.4	10.5	1
K2, 9 July 2006	2006Jul_38	9.1	1.3	20.7	9.2	16.1	43.6	2
K2, 9 July 2006	2006Jul_39	0.0	0.3	1.4	2.3	21.4	74.6	3
K2, 9 July 2006	2006Jul_40	6.9	1.7	8.5	11.9	24.4	46.7	3
K2, 9 July 2006	2006Jul_41	14.2	2.6	19.2	15.8	19.4	28.9	2
K2, 9 July 2006	2006Jul_42	16.3	2.7	23.8	14.8	16.5	25.9	2
K2, 9 July 2006	2006Jul_43	18.2	2.5	43.5	16.6	8.5	10.6	1
K2, 9 July 2006	2006Jul_44	17.8	2.2	40.9	15.3	8.4	15.4	1
K2, 9 July 2006	2006Jul_45	9.6	1.4	25.3	9.6	14.6	39.5	2
K2, 9 July 2006	2006Jul_46	8.9	2.8	11.0	12.1	24.1	41.0	3
K2, 9 July 2006	2006Jul_47	22.7	2.0	40.0	18.6	6.2	10.6	1
K2, 9 July 2006	2006Jul_48	22.6	2.3	39.6	16.7	6.5	12.3	1
K2, 9 July 2006	2006Jul_49	17.1	3.4	16.6	15.0	18.2	29.9	2
K2, 9 July 2006	2006Jul_50	16.8	2.8	41.1	16.6	8.9	13.9	1
K2, 9 July 2006	2006Jul_51	25.9	1.5	30.9	16.9	9.3	15.5	1
K2, 9 July 2006	2006Jul_52	17.1	2.6	36.0	15.5	10.6	18.3	1
K2, 9 July 2006	2006Jul_53	20.0	2.6	45.6	16.4	6.7	8.6	1

K2, 9 July 2006	2006Jul_54	16.7	2.4	43.4	17.1	8.0	12.5	1
K2, 9 July 2006	2006Jul_55	0.0	0.4	2.9	2.4	22.0	72.2	3
K2, 9 July 2006	2006Jul_56	19.1	2.5	48.4	19.4	4.3	6.3	1
K2, 9 July 2006	2006Jul_57	18.3	2.6	42.5	16.7	7.7	12.0	1
K2, 9 July 2006	2006Jul_58	19.9	1.8	43.6	20.8	5.2	8.7	1
K2, 9 July 2006	2006Jul_59	8.3	1.3	15.9	10.0	16.9	47.6	3
K2, 9 July 2006	2006Jul_60	23.7	2.9	23.4	18.2	11.6	20.3	2
K2, 9 July 2006	2006Jul_61	19.8	1.9	44.8	18.5	5.9	9.1	1
K2, 9 July 2006	2006Jul_62	20.3	2.3	33.2	17.3	10.8	16.1	1
K2, 9 July 2006	2006Jul_63	18.2	2.0	41.3	20.3	7.8	10.4	1
K2, 9 July 2006	2006Jul_64	18.6	2.4	36.7	16.7	9.2	16.4	1
K2, 9 July 2006	2006Jul_65	10.7	1.9	10.3	13.5	25.6	38.0	2
K2, 9 July 2006	2006Jul_66	16.9	2.6	41.5	16.8	10.6	11.6	1
K2, 9 July 2006	2006Jul_67	22.5	2.5	45.2	18.2	4.4	7.1	1
K2, 9 July 2006	2006Jul_68	22.2	1.9	48.4	17.9	3.7	5.9	1
K2, 9 July 2006	2006Jul_69	9.8	1.7	17.8	11.4	22.6	36.7	2
K2, 9 July 2006	2006Jul_70	20.3	2.0	46.5	19.1	5.1	6.9	1
K2, 9 July 2006	2006Jul_71	13.7	1.6	37.2	15.3	10.0	22.3	1
K2, 9 July 2006	2006Jul_72	13.0	2.0	29.8	12.5	14.7	28.0	2
K2, 9 July 2006	2006Jul_73	2.9	0.7	7.5	4.6	20.5	63.8	3
K2, 9 July 2006	2006Jul_74	21.6	2.3	46.7	19.2	4.0	6.2	1
K2, 9 July 2006	2006Jul_75	16.0	2.0	33.7	14.9	10.4	23.0	2
K2, 9 July 2006	2006Jul_76	6.6	1.0	15.7	7.3	17.9	51.6	3
K2, 9 July 2006	2006Jul_77	22.6	2.5	41.9	17.7	6.1	9.1	1
K2, 9 July 2006	2006Jul_78	20.0	1.8	38.0	14.2	7.1	18.9	1
K2, 9 July 2006	2006Jul_79	21.8	2.6	38.2	19.2	7.3	10.9	1
K2, 9 July 2006	2006Jul_80	16.8	1.8	31.7	17.3	12.9	19.5	2
K2, 9 July 2006	2006Jul_81	0.0	0.5	0.0	9.6	36.8	53.1	3
K2, 9 July 2006	2006Jul_82	7.6	1.1	21.3	9.4	13.7	46.9	3
K2, 9 July 2006	2006Jul_83	15.2	2.1	31.9	13.0	10.5	27.2	2
K2, 9 July 2006	2006Jul_84	18.4	2.5	34.9	14.5	11.4	18.2	1
K2, 9 July 2006	2006Jul_85	3.2	0.7	8.2	5.4	19.8	62.8	3
K2, 9 July 2006	2006Jul_86	21.8	1.6	42.0	18.9	6.5	9.3	1
K2, 9 July 2006	2006Jul_87	7.5	2.8	10.1	14.9	28.1	36.6	2
K2, 9 July 2006	2006Jul_88	24.5	2.5	31.1	23.4	7.8	10.6	1
K2, 9 July 2006	2006Jul_89	20.3	2.5	41.3	18.0	8.0	9.9	1
K2, 9 July 2006	2006Jul_90	15.2	2.2	33.1	15.5	13.3	20.9	2
K2, 9 July 2006	2006Jul_91	18.8	2.1	23.9	13.0	13.8	28.4	2
K2, 9 July 2006	2006Jul_92	18.2	2.6	46.7	17.0	6.1	9.4	1
K2, 9 July 2006	2006Jul_93	21.3	2.2	45.2	17.9	5.4	8.0	1
K2, 9 July 2006	2006Jul_94	18.8	1.9	43.2	19.4	6.4	10.3	1
K2, 9 July 2006	2006Jul_95	19.3	2.0	34.7	16.0	11.2	16.9	1
K2, 9 July 2006	2006Jul_96	19.6	2.7	45.4	17.5	6.6	8.1	1
K2, 9 July 2006	2006Jul_97	19.2	2.8	40.9	16.5	8.3	12.3	1
K2, 9 July 2006	2006Jul_98	21.3	2.6	47.8	16.7	3.9	7.8	1
K2, 9 July 2006	2006Jul_99	16.1	2.2	38.5	13.5	8.8	20.9	1
K2, 9 July 2006	2006Jul_100	8.0	1.7	10.8	10.6	18.2	50.6	3
K2, 9 July 2006	2006Jul_101	20.0	2.3	44.7	16.6	6.3	10.1	1
K2, 9 July 2006	2006Jul_102	15.2	1.5	41.3	16.2	7.1	18.8	1
K2, 9 July 2006	2006Jul_103	19.4	2.5	46.2	18.4	4.6	8.9	1
K2, 9 July 2006	2006Jul_104	39.1	0.0	4.5	14.5	13.2	28.8	2
K2, 9 July 2006	2006Jul_105	0.0	0.1	0.0	9.9	43.3	46.7	3
K2, 9 July 2006	2006Jul_106	13.0	2.0	30.1	13.4	13.8	27.6	2
K2, 9 July 2006	2006Jul_107	20.8	2.2	32.1	19.6	10.2	15.1	1
K2, 9 July 2006	2006Jul_108	14.1	1.4	25.9	16.1	14.7	27.9	2
K2, 9 July 2006	2006Jul_109	17.7	0.9	42.7	14.7	9.8	14.2	1
K2, 9 July 2006	2006Jul_110	17.5	0.4	46.8	16.7	7.7	11.0	1
K2, 9 July 2006	2006Jul_111	18.3	2.3	42.4	16.3	8.7	12.0	1
K2, 9 July 2006	2006Jul_112	19.4	1.9	43.6	14.8	6.7	13.7	1
K2, 9 July 2006	2006Jul_113	13.7	2.1	34.6	15.0	14.2	20.4	2
K2, 9 July 2006	2006Jul_114	0.9	0.0	8.5	3.3	20.0	67.4	3
K2, 9 July 2006	2006Jul_115	10.8	0.9	28.2	9.3	12.9	37.8	2
K2, 9 July 2006	2006Jul_116	17.0	1.1	48.1	17.1	5.4	11.3	1
K2, 9 July 2006	2006Jul_117	0.0	0.6	0.0	7.4	24.9	67.1	3
K2, 9 July 2006	2006Jul_118	18.6	2.2	30.0	14.5	11.9	22.8	2
K2, 9 July 2006	2006Jul_119	17.5	2.4	23.2	18.1	14.1	24.7	2
K2, 9 July 2006	2006Jul_120	18.7	2.3	39.0	18.4	9.1	12.6	1
K2, 9 July 2006	2006Jul_121	17.5	2.5	38.4	17.9	8.6	15.0	1
K2, 9 July 2006	2006Jul_122	18.3	1.9	45.2	18.6	7.0	9.0	1
K2, 9 July 2006	2006Jul_123	1.7	0.7	6.9	4.9	19.9	65.7	3
K2, 9 July 2006	2006Jul_124	13.3	1.9	35.0	18.1	11.3	20.5	2
K2, 9 July 2006	2006Jul_125	16.9	2.1	46.4	18.9	4.0	11.6	1
K2, 9 July 2006	2006Jul_126	16.3	1.8	43.3	18.3	7.6	12.8	1
K2, 9 July 2006	2006Jul_127	13.8	1.5	37.7	17.0	7.6	22.3	1
K2, 9 July 2006	2006Jul_128	15.1	2.5	41.7	18.5	5.4	16.8	1
K2, 9 July 2006	2006Jul_129	17.9	2.3	42.4	17.2	7.6	12.6	1
K2, 9 July 2006	2006Jul_130	16.8	2.2	44.2	22.1	5.0	9.8	1
K2, 9 July 2006	2006Jul_131	18.0	2.0	39.8	17.5	8.0	14.6	1
K2, 9 July 2006	2006Jul_132	9.4	3.5	12.0	10.3	19.0	45.9	3
K2, 9 July 2006	2006Jul_133	16.4	0.8	25.1	23.2	17.1	17.5	2
K2, 9 July 2006	2006Jul_134	11.2	2.0	31.1	17.4	14.5	23.8	2
K2, 9 July 2006	2006Jul_135	24.2	1.4	45.7	17.1	5.6	6.0	1
K2, 9 July 2006	2006Jul_136	19.3	2.3	43.6	20.4	6.2	8.3	1
K2, 9 July 2006	2006Jul_137	16.6	1.6	47.9	18.2	5.9	9.7	1
K2, 9 July 2006	2006Jul_138	12.5	1.8	28.4	15.9	14.4	26.9	2
K2, 9 July 2006	2006Jul_139	15.3	1.6	27.6	15.8	8.6	31.1	2

K2, 9 July 2006	2006Jul_140	7.9	2.1	19.6	13.7	16.7	39.9	2
K2, 9 July 2006	2006Jul_141	13.4	1.6	38.1	16.5	10.7	19.7	1
K2, 9 July 2006	2006Jul_142	4.2	0.9	13.4	7.4	19.4	54.7	3
K2, 9 July 2006	2006Jul_143	13.4	2.2	43.9	16.9	6.8	16.8	1
K2, 9 July 2006	2006Jul_144	10.2	1.3	23.6	11.0	13.7	40.2	2
K2, 9 July 2006	2006Jul_145	17.1	2.2	46.0	18.4	6.5	9.8	1
K2, 9 July 2006	2006Jul_146	19.9	1.9	49.7	19.2	3.4	5.9	1
K2, 9 July 2006	2006Jul_147	18.1	1.4	42.4	18.1	7.5	12.6	1
K2, 9 July 2006	2006Jul_148	0.0	0.4	3.5	2.2	20.3	73.7	3
K2, 9 July 2006	2006Jul_149	13.3	1.8	24.6	13.3	14.3	32.7	2
K2, 9 July 2006	2006Jul_150	11.1	1.6	26.5	21.6	14.9	24.3	2
K2, 9 July 2006	2006Jul_151	15.8	1.9	34.1	17.4	11.7	19.1	1
K2, 9 July 2006	2006Jul_152	19.3	1.9	44.7	18.4	6.3	9.3	1
K2, 9 July 2006	2006Jul_153	15.4	1.8	42.5	17.4	8.3	14.7	1
K2, 9 July 2006	2006Jul_154	13.0	2.0	17.2	16.3	18.7	32.9	2
K2, 9 July 2006	2006Jul_155	16.0	2.4	48.1	19.0	4.4	10.0	1
K2, 9 July 2006	2006Jul_156	14.1	2.4	44.7	17.2	7.0	14.6	1
K2, 9 July 2006	2006Jul_157	13.3	1.7	35.4	16.6	14.1	18.8	1
K2, 9 July 2006	2006Jul_158	16.7	2.1	29.7	17.1	14.4	20.0	2
K2, 9 July 2006	2006Jul_159	14.0	2.1	37.0	17.7	11.0	18.2	1
K2, 9 July 2006	2006Jul_160	17.0	2.0	42.7	18.4	6.3	13.6	1
K2, 9 July 2006	2006Jul_161	11.1	1.6	20.7	14.6	17.4	34.6	2
K2, 9 July 2006	2006Jul_162	19.7	2.4	25.0	19.3	14.5	19.1	2
K2, 9 July 2006	2006Jul_163	13.4	2.0	35.1	16.4	12.4	20.8	2
K2, 9 July 2006	2006Jul_164	23.8	3.0	26.0	14.3	11.3	21.6	2
K2, 9 July 2006	2006Jul_165	18.6	1.9	42.1	17.0	6.5	14.0	1
K2, 9 July 2006	2006Jul_166	14.5	1.9	24.3	15.8	13.2	30.3	2
K2, 9 July 2006	2006Jul_167	19.5	2.2	43.6	20.2	3.7	10.8	1
K2, 9 July 2006	2006Jul_168	17.8	2.7	50.5	15.7	0.7	12.5	1
K2, 9 July 2006	2006Jul_169	16.2	2.1	35.5	17.3	8.2	20.7	1
K2, 9 July 2006	2006Jul_170	7.3	1.1	19.5	8.2	13.9	49.8	3
K2, 9 July 2006	2006Jul_171	18.5	2.4	45.7	18.7	5.2	9.5	1
K2, 9 July 2006	2006Jul_172	16.6	1.9	47.2	18.4	6.0	9.9	1
K2, 9 July 2006	2006Jul_173	15.1	2.0	36.3	14.9	8.6	23.1	1
K2, 9 July 2006	2006Jul_174	19.6	2.1	47.4	18.0	4.1	8.7	1
K2, 9 July 2006	2006Jul_175	17.6	1.5	34.6	21.0	8.3	16.9	1
K2, 9 July 2006	2006Jul_176	10.8	2.2	35.0	16.0	10.4	25.7	2
K2, 9 July 2006	2006Jul_177	15.5	1.9	37.0	16.0	9.6	20.0	1
K2, 9 July 2006	2006Jul_178	6.5	3.2	24.8	8.7	9.3	47.5	2
K2, 9 July 2006	2006Jul_179	36.2	0.0	2.3	13.9	14.4	33.2	2
K2, 9 July 2006	2006Jul_180	11.9	2.3	32.1	13.5	11.9	28.2	2
K2, 9 July 2006	2006Jul_181	6.4	1.0	18.1	8.0	13.8	52.7	3
K2, 9 July 2006	2006Jul_182	18.8	1.9	43.9	18.0	3.5	14.0	1
K2, 9 July 2006	2006Jul_183	24.5	0.4	40.5	17.0	6.2	11.4	1
K2, 9 July 2006	2006Jul_184	21.7	1.3	34.8	17.1	6.2	18.9	1
K2, 9 July 2006	2006Jul_185	31.2	0.0	24.8	16.4	6.9	20.6	2
K2, 9 July 2006	2006Jul_186	18.2	2.5	47.1	17.2	5.5	9.6	1
K2, 9 July 2006	2006Jul_187	6.4	0.9	20.1	9.7	12.6	50.3	3
K2, 9 July 2006	2006Jul_188	5.9	4.1	23.3	10.7	14.1	41.9	2
K2, 9 July 2006	2006Jul_189	17.1	1.9	47.3	18.7	5.7	9.3	1
K2, 9 July 2006	2006Jul_190	17.5	1.7	42.9	21.0	6.2	10.7	1
K2, 21 February 2021	2021Feb_1	10.9	2.3	26.1	8.4	19.3	33.0	2
K2, 21 February 2021	2021Feb_2	0.1	0.0	0.0	7.6	38.3	54.0	3
K2, 21 February 2021	2021Feb_3	19.0	3.8	38.9	11.3	9.3	17.7	1
K2, 21 February 2021	2021Feb_4	7.2	1.3	16.3	6.3	21.4	47.5	3
K2, 21 February 2021	2021Feb_5	19.7	4.6	29.8	10.2	12.3	23.4	2
K2, 21 February 2021	2021Feb_6	2.5	2.1	9.9	3.9	1.6	79.9	3
K2, 21 February 2021	2021Feb_7	21.4	2.8	28.3	15.1	13.0	19.4	2
K2, 21 February 2021	2021Feb_8	21.0	3.4	28.0	15.4	13.8	18.5	2
K2, 21 February 2021	2021Feb_9	10.7	2.2	18.9	7.8	17.1	43.2	2
K2, 21 February 2021	2021Feb_10	37.3	3.3	32.8	12.0	6.9	7.7	1
K2, 21 February 2021	2021Feb_11	23.8	3.4	43.1	11.0	7.9	10.7	1
K2, 21 February 2021	2021Feb_12	20.1	2.5	41.7	11.9	10.0	13.8	1
K2, 21 February 2021	2021Feb_13	1.2	0.7	3.5	2.8	24.2	67.6	3
K2, 21 February 2021	2021Feb_14	12.8	1.6	25.3	8.1	16.2	35.9	2
K2, 21 February 2021	2021Feb_15	18.2	2.3	23.8	11.8	16.0	28.0	2
K2, 21 February 2021	2021Feb_16	17.4	1.8	13.9	12.2	20.1	34.7	2
K2, 21 February 2021	2021Feb_17	22.6	3.3	43.8	11.0	8.6	10.7	1
K2, 21 February 2021	2021Feb_18	14.5	3.2	19.0	10.3	17.8	35.2	2
K2, 21 February 2021	2021Feb_19	13.1	2.4	30.9	9.1	15.4	29.0	2
K2, 21 February 2021	2021Feb_20	19.1	2.8	33.2	13.1	13.6	18.2	1
K2, 21 February 2021	2021Feb_21	16.1	2.7	32.1	9.8	14.4	24.9	2
K2, 21 February 2021	2021Feb_22	20.0	3.4	40.0	11.9	10.9	13.8	1
K2, 21 February 2021	2021Feb_23	0.2	0.0	0.0	6.7	34.6	58.5	3
K2, 21 February 2021	2021Feb_24	12.6	4.3	26.0	10.8	16.8	29.6	2
K2, 21 February 2021	2021Feb_25	4.5	1.4	16.4	4.4	17.8	55.5	3
K2, 21 February 2021	2021Feb_26	17.8	3.0	39.4	11.5	11.9	16.5	1
K2, 21 February 2021	2021Feb_27	18.3	3.0	45.6	13.3	8.1	11.6	1
K2, 21 February 2021	2021Feb_28	6.9	2.6	20.7	8.7	21.0	40.1	2
K2, 21 February 2021	2021Feb_29	22.6	3.7	43.8	10.2	7.5	12.1	1
K2, 21 February 2021	2021Feb_30	12.8	2.1	23.9	13.1	19.3	29.0	2
K2, 21 February 2021	2021Feb_31	0.0	0.2	0.0	10.2	40.6	49.0	3
K2, 21 February 2021	2021Feb_32	18.7	3.1	39.9	12.8	9.1	16.4	1
K2, 21 February 2021	2021Feb_33	1.7	2.4	5.7	9.6	27.2	53.3	3
K2, 21 February 2021	2021Feb_34	14.9	4.4	34.7	10.4	11.7	23.8	2
K2, 21 February 2021	2021Feb_35	21.1	3.4	43.3	11.6	9.6	10.9	1

K2, 21 February 2021	2021Feb_36	27.0	1.7	40.1	13.8	7.7	9.8	1
K2, 21 February 2021	2021Feb_37	9.4	1.9	24.7	6.6	15.6	41.8	2
K2, 21 February 2021	2021Feb_38	22.5	3.4	38.0	13.9	9.5	12.7	1
K2, 21 February 2021	2021Feb_39	9.5	3.0	15.9	11.0	24.0	36.7	2
K2, 21 February 2021	2021Feb_40	22.5	2.9	47.9	11.7	6.2	8.8	1
K2, 21 February 2021	2021Feb_41	4.8	0.0	0.0	14.8	33.5	46.8	3
K2, 21 February 2021	2021Feb_42	21.6	3.3	46.1	11.7	7.6	9.6	1
K2, 21 February 2021	2021Feb_43	17.9	1.4	17.1	7.6	22.4	33.6	2
K2, 21 February 2021	2021Feb_44	19.6	2.4	39.6	10.7	12.2	15.5	1
K2, 21 February 2021	2021Feb_45	16.0	2.9	36.7	11.6	15.2	17.7	1
K2, 21 February 2021	2021Feb_46	22.3	3.6	38.8	11.3	10.3	13.7	1
K2, 21 February 2021	2021Feb_47	16.8	3.0	39.0	11.2	12.4	17.4	1
K2, 21 February 2021	2021Feb_48	19.5	3.1	36.0	11.4	12.7	17.3	1
K2, 21 February 2021	2021Feb_49	18.7	3.2	36.8	10.5	11.4	19.5	1
K2, 21 February 2021	2021Feb_50	6.9	1.6	18.7	7.0	17.4	48.5	3
K2, 21 February 2021	2021Feb_51	24.1	3.2	35.0	12.3	6.8	18.6	1
K2, 21 February 2021	2021Feb_52	25.4	3.3	46.1	10.9	6.8	7.4	1
K2, 21 February 2021	2021Feb_53	21.7	3.5	48.7	10.6	5.8	9.7	1
K2, 21 February 2021	2021Feb_54	22.8	3.1	49.2	11.0	6.4	7.5	1
K2, 21 February 2021	2021Feb_55	15.8	2.5	19.9	11.5	15.4	34.9	2
K2, 21 February 2021	2021Feb_56	6.2	3.1	38.6	12.3	0.4	39.3	2
K2, 21 February 2021	2021Feb_57	5.9	2.0	29.9	9.9	0.5	51.7	2
K2, 21 February 2021	2021Feb_58	4.8	1.0	17.4	9.0	14.5	53.4	3
K2, 21 February 2021	2021Feb_59	17.1	1.7	41.2	24.2	5.5	10.3	1
K2, 21 February 2021	2021Feb_60	1.9	2.2	9.6	10.5	22.7	53.1	3
K2, 21 February 2021	2021Feb_61	6.0	1.1	18.8	10.5	15.0	48.6	3
K2, 21 February 2021	2021Feb_62	0.3	0.9	0.0	3.5	17.0	78.3	3
K2, 21 February 2021	2021Feb_63	13.1	2.8	35.5	15.3	8.1	25.3	2
K2, 21 February 2021	2021Feb_64	12.9	1.7	26.1	17.5	13.7	28.1	2
K2, 21 February 2021	2021Feb_65	15.7	2.1	37.1	18.1	12.1	14.9	1
K2, 21 February 2021	2021Feb_66	0.0	0.5	0.0	6.0	29.9	63.6	3
K2, 21 February 2021	2021Feb_67	17.1	1.9	27.3	15.1	14.7	24.0	2
K2, 21 February 2021	2021Feb_68	0.0	0.1	0.0	2.1	19.9	77.8	3
K2, 21 February 2021	2021Feb_69	28.1	2.2	30.8	18.9	8.4	11.5	1
K2, 21 February 2021	2021Feb_70	20.6	1.7	25.4	20.7	8.5	23.2	2
K2, 21 February 2021	2021Feb_71	19.8	3.1	15.1	15.1	19.5	27.4	2
K2, 21 February 2021	2021Feb_72	24.7	1.7	29.0	20.7	9.2	14.8	1
K2, 21 February 2021	2021Feb_73	14.7	2.4	20.2	14.9	14.8	33.1	2
K2, 21 February 2021	2021Feb_74	22.7	2.0	33.9	18.5	8.4	14.5	1
K2, 21 February 2021	2021Feb_75	26.4	0.7	17.0	21.7	14.5	19.7	2
K2, 21 February 2021	2021Feb_76	8.3	2.0	11.2	13.4	24.8	40.3	3
K2, 21 February 2021	2021Feb_77	20.8	1.8	45.3	18.0	4.9	9.2	1
K2, 21 February 2021	2021Feb_78	10.3	2.5	8.5	10.1	21.9	46.8	3
K2, 21 February 2021	2021Feb_79	11.4	1.5	31.6	14.0	10.9	30.5	2
K2, 21 February 2021	2021Feb_80	15.5	2.2	36.8	16.0	8.7	20.7	1
K2, 21 February 2021	2021Feb_81	20.7	2.8	21.9	17.8	16.2	20.6	2
K2, 21 February 2021	2021Feb_82	5.0	2.5	15.7	12.8	20.5	43.5	3
K2, 21 February 2021	2021Feb_83	11.7	1.4	24.7	12.2	14.2	35.7	2
K2, 21 February 2021	2021Feb_84	21.8	1.4	35.9	17.2	9.3	14.5	1
K2, 21 February 2021	2021Feb_85	12.0	2.3	31.9	15.3	11.4	27.2	2
K2, 21 February 2021	2021Feb_86	20.9	2.5	42.0	19.1	5.3	10.2	1
K2, 21 February 2021	2021Feb_87	19.9	2.4	30.5	16.7	11.3	19.2	2
K2, 21 February 2021	2021Feb_88	6.2	1.1	0.0	8.2	35.9	48.6	3
K2, 21 February 2021	2021Feb_89	21.1	1.7	44.6	21.4	4.9	6.3	1
K2, 21 February 2021	2021Feb_90	5.3	1.1	10.9	8.1	21.0	53.6	3
K2, 21 February 2021	2021Feb_91	23.1	1.5	22.4	17.1	13.2	22.7	2
K2, 21 February 2021	2021Feb_92	24.2	2.7	21.7	18.4	12.0	20.9	2
K2, 21 February 2021	2021Feb_93	8.0	3.6	37.5	12.5	5.7	32.6	2
K2, 21 February 2021	2021Feb_94	11.8	1.6	20.1	16.3	16.7	33.5	2
K2, 21 February 2021	2021Feb_95	20.9	1.4	36.1	23.8	7.3	10.6	1
K2, 21 February 2021	2021Feb_96	21.3	2.6	49.0	16.6	3.5	7.0	1
K2, 21 February 2021	2021Feb_97	19.2	1.8	43.0	18.9	5.3	11.9	1
K2, 21 February 2021	2021Feb_98	23.8	1.9	33.9	18.7	7.8	13.8	1
K2, 21 February 2021	2021Feb_99	0.0	0.5	0.0	2.5	17.1	79.9	3
K2, 21 February 2021	2021Feb_100	14.2	1.7	32.4	20.4	11.3	20.0	2
K2, 21 February 2021	2021Feb_101	16.8	1.9	44.1	19.7	6.5	11.0	1
K2, 21 February 2021	2021Feb_102	17.9	0.6	41.5	23.1	6.6	10.3	1
K2, 21 February 2021	2021Feb_103	1.1	0.7	6.5	4.7	16.6	70.3	3
K2, 21 February 2021	2021Feb_104	14.6	1.0	50.1	19.9	3.7	10.7	1
K2, 21 February 2021	2021Feb_105	4.5	0.9	14.4	6.0	14.2	60.0	3
K2, 3 May 2022	2022May_1	25.7	1.3	10.8	16.1	15.6	30.3	2
K2, 3 May 2022	2022May_2	19.5	1.9	37.4	20.8	6.9	13.6	1
K2, 3 May 2022	2022May_3	15.9	0.9	31.5	20.1	10.8	20.7	2
K2, 3 May 2022	2022May_4	15.8	1.2	36.7	18.8	8.5	19.1	1
K2, 3 May 2022	2022May_5	6.3	0.8	14.5	10.1	14.4	53.9	3
K2, 3 May 2022	2022May_6	20.7	0.4	29.8	17.9	13.4	17.8	2
K2, 3 May 2022	2022May_7	29.3	0.1	12.8	14.8	15.2	27.8	2
K2, 3 May 2022	2022May_8	17.3	1.5	41.2	21.5	5.7	12.7	1
K2, 3 May 2022	2022May_9	17.9	0.5	28.6	18.5	8.3	26.2	2
K2, 3 May 2022	2022May_10	20.0	1.0	27.0	19.7	12.9	19.3	2
K2, 3 May 2022	2022May_11	16.8	1.5	37.2	18.4	8.3	17.7	1
K2, 3 May 2022	2022May_12	12.0	0.1	16.1	10.7	14.9	46.1	3
K2, 3 May 2022	2022May_13	10.1	1.3	23.1	15.9	12.8	36.9	2
K2, 3 May 2022	2022May_14	24.4	1.8	17.4	19.5	14.8	22.1	2
K2, 3 May 2022	2022May_15	0.0	0.3	2.3	2.7	18.5	76.3	3
K2, 3 May 2022	2022May_16	18.2	1.4	16.7	20.2	15.0	28.5	2

K2, 3 May 2022	2022May_17	15.4	1.5	33.1	20.2	11.4	18.3	1
K2, 3 May 2022	2022May_18	0.0	0.4	5.2	3.3	18.4	72.6	3
K2, 3 May 2022	2022May_19	16.6	1.6	39.1	19.0	7.5	16.2	1
K2, 3 May 2022	2022May_20	22.4	0.2	22.2	20.6	15.1	19.6	2
K2, 3 May 2022	2022May_21	16.8	0.0	9.8	17.1	14.6	41.7	2
K2, 3 May 2022	2022May_22	16.8	1.5	40.4	19.1	6.8	15.4	1
K2, 3 May 2022	2022May_23	14.9	1.4	19.0	15.9	16.5	32.3	2
K2, 3 May 2022	2022May_24	12.6	1.5	7.0	9.1	19.7	50.2	3
K2, 3 May 2022	2022May_25	15.6	1.0	17.8	13.8	15.7	36.1	2
K2, 3 May 2022	2022May_26	10.5	0.8	22.9	11.6	13.7	40.6	2
K2, 3 May 2022	2022May_27	18.0	1.3	42.1	19.3	6.8	12.6	1
K2, 3 May 2022	2022May_28	0.0	0.4	0.0	6.3	31.3	62.0	3
K2, 3 May 2022	2022May_29	24.2	1.5	13.1	20.5	15.5	25.2	2
K2, 3 May 2022	2022May_30	16.4	0.7	9.7	15.9	20.9	36.5	2
K2, 3 May 2022	2022May_31	18.9	1.5	44.1	17.3	7.1	11.0	1
K2, 3 May 2022	2022May_32	23.7	0.0	4.4	15.9	15.8	40.2	2
K2, 3 May 2022	2022May_33	20.1	1.8	40.0	20.9	5.3	11.9	1
K2, 3 May 2022	2022May_34	5.6	0.7	5.5	8.6	16.1	63.5	3
K2, 3 May 2022	2022May_35	21.6	1.7	27.9	20.7	9.2	18.9	2
K2, 3 May 2022	2022May_36	22.1	1.9	34.5	14.7	8.2	18.5	1
K2, 3 May 2022	2022May_37	14.6	1.1	41.3	21.8	6.5	14.7	1
K2, 3 May 2022	2022May_38	22.5	1.8	16.7	20.4	14.2	24.4	2
K2, 3 May 2022	2022May_39	17.6	1.4	34.3	17.4	9.8	19.5	1
K2, 3 May 2022	2022May_40	20.0	0.4	5.5	15.4	19.9	38.8	2
K2, 3 May 2022	2022May_41	8.1	0.9	6.3	10.9	18.5	55.3	3
K2, 3 May 2022	2022May_42	26.4	0.0	25.6	18.6	8.6	20.8	2
K2, 3 May 2022	2022May_43	20.4	0.0	7.1	12.4	18.6	41.5	2
K2, 3 May 2022	2022May_44	4.0	0.1	4.4	3.2	22.1	66.3	3
K2, 3 May 2022	2022May_45	16.7	1.4	33.1	19.9	8.2	20.7	1
K2, 3 May 2022	2022May_46	17.6	1.5	27.8	15.9	11.9	25.3	2
K2, 3 May 2022	2022May_47	30.5	0.7	31.4	16.5	8.3	12.6	1
K2, 3 May 2022	2022May_48	15.2	0.3	4.7	13.1	20.0	46.7	3
K2, 3 May 2022	2022May_49	18.1	1.6	38.0	18.4	7.4	16.4	1
K2, 3 May 2022	2022May_50	22.0	2.1	14.1	12.9	19.4	29.5	2
K2, 3 May 2022	2022May_51	0.9	0.4	2.7	3.5	21.9	70.6	3
K2, 3 May 2022	2022May_52	20.6	2.1	42.1	18.8	6.6	9.8	1
K2, 3 May 2022	2022May_53	19.8	1.5	41.9	22.5	5.6	8.6	1
K2, 3 May 2022	2022May_54	18.4	1.5	31.6	18.4	10.4	19.7	2
K2, 3 May 2022	2022May_55	17.7	0.9	10.8	15.3	16.8	38.4	2
K2, 3 May 2022	2022May_56	28.2	0.9	36.7	20.3	5.9	7.9	1
K2, 3 May 2022	2022May_57	14.9	1.6	24.9	14.7	14.7	29.3	2
K2, 3 May 2022	2022May_58	17.1	2.0	32.3	23.5	9.2	15.8	1
K2, 3 May 2022	2022May_59	21.6	1.1	32.8	19.0	9.9	15.6	1
K2, 3 May 2022	2022May_60	0.5	0.3	3.2	2.7	19.0	74.3	3
K2, 3 May 2022	2022May_61	11.9	1.6	30.8	14.7	13.4	27.7	2
K2, 3 May 2022	2022May_62	13.7	2.0	38.7	18.0	8.8	18.8	1
K2, 3 May 2022	2022May_63	4.1	1.0	9.9	5.5	21.5	58.0	3
K2, 3 May 2022	2022May_64	22.7	2.0	31.5	18.3	12.2	13.3	1
K2, 3 May 2022	2022May_65	17.2	0.8	32.8	16.2	10.5	22.5	2
K2, 3 May 2022	2022May_66	27.1	1.9	25.3	17.8	11.1	16.7	2
K2, 3 May 2022	2022May_67	26.9	0.0	13.7	9.6	9.7	40.1	2
K2, 3 May 2022	2022May_68	28.3	1.8	25.1	16.0	12.8	16.0	2
K2, 3 May 2022	2022May_69	23.6	0.7	21.9	19.5	14.0	20.4	2
K2, 3 May 2022	2022May_70	15.3	1.6	29.9	22.1	11.4	19.7	2
K2, 3 May 2022	2022May_71	25.7	1.2	32.1	18.3	9.4	13.4	1
K2, 3 May 2022	2022May_72	17.1	0.8	22.7	20.2	14.6	24.6	2
K2, 3 May 2022	2022May_73	11.5	1.3	17.4	10.9	13.0	45.9	2
K2, 3 May 2022	2022May_74	12.0	0.0	2.4	6.7	15.0	63.8	3
K2, 3 May 2022	2022May_75	13.2	0.9	30.5	15.9	10.0	29.6	2
K2, 3 May 2022	2022May_76	19.3	2.1	37.5	17.9	7.8	15.3	1
K2, 3 May 2022	2022May_77	17.5	2.1	40.8	16.7	6.6	16.3	1
K2, 3 May 2022	2022May_78	15.5	1.8	38.8	15.3	4.9	23.7	1
K2, 3 May 2022	2022May_79	10.4	0.9	29.3	14.1	9.4	35.9	2
K2, 3 May 2022	2022May_80	12.9	1.7	30.3	15.9	10.7	28.4	2
K2, 3 May 2022	2022May_81	18.5	1.8	33.8	16.0	9.0	20.9	1
K2, 3 May 2022	2022May_82	21.7	2.0	35.1	20.8	9.2	11.3	1
K2, 3 May 2022	2022May_83	18.0	2.7	43.3	15.7	6.8	13.5	1
K2, 3 May 2022	2022May_84	16.1	2.1	42.2	17.5	7.7	14.4	1
K2, 3 May 2022	2022May_85	21.2	1.1	39.2	20.1	6.7	11.7	1
K2, 3 May 2022	2022May_86	7.5	1.0	25.5	11.1	11.5	43.4	2
K2, 3 May 2022	2022May_87	14.8	1.8	29.5	15.2	11.2	27.6	2
K2, 3 May 2022	2022May_88	20.2	2.0	36.2	17.5	7.9	16.1	1
K2, 3 May 2022	2022May_89	17.6	1.7	39.1	17.9	7.1	16.7	1
K2, 3 May 2022	2022May_90	16.6	2.1	41.3	19.0	5.9	15.2	1
K2, 3 May 2022	2022May_91	18.2	2.1	44.6	18.2	5.1	11.8	1
K2, 3 May 2022	2022May_92	17.3	2.1	40.5	16.9	8.4	14.9	1
K2, 3 May 2022	2022May_93	17.5	1.8	40.9	19.5	6.7	13.6	1
K2, 3 May 2022	2022May_94	17.3	2.8	21.7	13.0	14.5	30.7	2
K2, 3 May 2022	2022May_95	15.4	1.8	24.6	14.5	13.0	30.8	2
K2, 3 May 2022	2022May_96	16.7	1.7	41.0	19.4	7.3	14.0	1
K2, 3 May 2022	2022May_97	22.4	2.0	41.4	16.3	6.4	11.5	1
K2, 3 May 2022	2022May_98	19.0	1.9	38.6	19.4	7.4	13.7	1
K2, 3 May 2022	2022May_99	18.0	1.1	38.7	19.1	5.6	17.5	1
K2, 3 May 2022	2022May_100	28.3	1.7	25.0	16.9	8.4	19.6	2
K2, 3 May 2022	2022May_101	20.1	1.4	22.4	18.2	12.1	25.8	2
K2, 3 May 2022	2022May_102	15.4	1.9	16.4	11.7	14.3	40.4	2

K2, 3 May 2022	2022May_103	0.0	0.8	0.0	3.1	15.6	80.4	3
K2, 3 May 2022	2022May_104	22.3	1.7	36.5	17.6	7.3	14.6	1
K2, 3 May 2022	2022May_105	17.9	1.5	30.0	18.2	10.2	22.4	2
K2, 3 May 2022	2022May_106	18.0	1.1	9.1	11.7	21.9	38.2	2
K2, 3 May 2022	2022May_107	5.2	0.1	8.8	7.3	17.9	60.7	3
K2, 3 May 2022	2022May_108	12.8	1.2	22.0	14.2	13.8	36.0	2
K2, 3 May 2022	2022May_109	12.8	1.5	33.6	15.0	8.9	28.2	2
K2, 3 May 2022	2022May_110	15.2	1.0	7.8	11.2	16.0	48.9	3
K2, 3 May 2022	2022May_111	23.7	0.4	29.7	17.4	9.3	19.5	1
K2, 3 May 2022	2022May_112	4.3	0.6	8.3	6.1	16.8	63.9	3
K2, 3 May 2022	2022May_113	13.4	1.6	35.3	16.9	8.4	24.3	2
K2, 3 May 2022	2022May_114	1.6	0.5	5.5	4.9	18.5	69.1	3
K2, 3 May 2022	2022May_115	16.6	1.6	39.3	17.3	7.0	18.3	1
K2, 3 May 2022	2022May_116	11.6	0.9	10.4	11.3	15.6	50.3	3
K2, 3 May 2022	2022May_117	20.0	1.3	21.7	15.5	11.0	30.5	2
K2, 3 May 2022	2022May_118	21.5	1.6	41.1	17.7	5.2	12.8	1
K2, 3 May 2022	2022May_119	11.7	1.2	12.8	10.6	17.7	45.9	3
K2, 3 May 2022	2022May_120	19.3	1.9	40.4	17.3	7.1	14.1	1
K2, 3 May 2022	2022May_121	26.6	0.3	4.4	12.6	17.4	38.9	2
K2, 3 May 2022	2022May_122	13.7	1.7	30.2	14.6	10.9	29.0	2
K2, 3 May 2022	2022May_123	7.1	0.9	18.2	8.3	13.2	52.3	3
K2, 3 May 2022	2022May_124	28.3	1.7	25.0	16.9	8.4	19.6	2
K2, 3 May 2022	2022May_125	19.4	1.8	41.3	16.9	8.0	12.5	1
K2, 3 May 2022	2022May_126	19.7	1.2	37.5	20.5	7.2	14.0	1
K2, 3 May 2022	2022May_127	15.8	2.0	35.2	15.8	11.5	19.8	1
K2, 3 May 2022	2022May_128	17.0	1.6	30.8	15.4	11.3	23.9	2
K2, 3 May 2022	2022May_129	19.2	1.9	43.1	16.6	6.0	13.2	1
K2, 3 May 2022	2022May_130	26.1	1.9	19.9	15.4	12.8	23.8	2
K2, 3 May 2022	2022May_131	5.5	0.7	12.6	7.6	15.4	58.2	3
K2, 3 May 2022	2022May_132	20.3	1.5	27.4	19.4	10.3	21.0	2
K2, 3 May 2022	2022May_133	18.9	1.9	36.1	19.7	9.0	14.6	1
K2, 3 May 2022	2022May_134	14.0	1.0	45.7	21.3	5.1	12.9	1
K2, 3 May 2022	2022May_135	16.5	1.4	29.2	15.5	10.9	26.5	2
K2, 3 May 2022	2022May_136	17.1	1.9	33.6	17.1	7.6	22.7	2
K2, 3 May 2022	2022May_137	18.9	2.2	32.9	13.9	9.5	22.6	2
K2, 3 May 2022	2022May_138	30.6	1.8	32.5	13.1	5.3	16.7	1
K2, 3 May 2022	2022May_139	17.6	1.6	22.1	15.4	12.6	30.7	2
K2, 3 May 2022	2022May_140	16.3	1.8	37.0	14.8	7.2	22.9	1
K2, 3 May 2022	2022May_141	14.2	1.8	32.8	19.1	9.2	22.9	2
K2, 3 May 2022	2022May_142	15.6	0.3	12.4	16.5	18.6	36.6	2
K2, 3 May 2022	2022May_143	14.8	1.6	39.5	17.8	7.6	18.6	1
K2, 3 May 2022	2022May_144	17.1	1.9	35.3	16.6	8.9	20.3	1
K2, 3 May 2022	2022May_145	15.2	1.6	34.0	14.5	10.1	24.7	2
K2, 3 May 2022	2022May_146	17.4	1.5	33.2	15.6	8.4	23.9	2
K2, 3 May 2022	2022May_147	0.1	0.3	0.4	2.4	18.8	78.0	3
K2, 3 May 2022	2022May_148	2.3	0.8	4.9	4.0	17.9	70.0	3
K2, 3 May 2022	2022May_149	0.3	0.3	1.4	2.5	16.5	79.1	3
K2, 3 May 2022	2022May_150	16.4	1.3	31.1	16.6	11.0	23.5	2
K2, 3 May 2022	2022May_151	15.5	1.1	20.0	12.9	11.9	38.6	2
K2, 3 May 2022	2022May_152	19.3	1.8	38.5	17.3	8.1	14.9	1
K2, 3 May 2022	2022May_153	14.5	1.6	36.5	16.9	8.9	21.5	1
K2, 3 May 2022	2022May_154	24.4	1.6	38.0	16.6	6.5	13.0	1
K2, 3 May 2022	2022May_155	23.6	1.4	27.9	14.2	8.9	23.9	2
K2, 3 May 2022	2022May_156	7.9	1.1	10.5	11.5	19.1	49.9	3
K2, 3 May 2022	2022May_157	18.3	1.8	35.6	16.6	8.7	19.0	1
K2, 3 May 2022	2022May_158	22.2	1.4	38.1	19.4	6.2	12.9	1
K2, 3 May 2022	2022May_159	1.9	0.6	4.0	3.6	15.9	74.0	3
K2, 3 May 2022	2022May_160	13.4	1.7	29.3	15.3	11.0	29.2	2
K2, 3 May 2022	2022May_161	14.5	1.7	25.4	15.1	12.0	31.2	2
K2, 3 May 2022	2022May_162	19.6	1.4	39.4	17.8	7.6	14.3	1
K2, 3 May 2022	2022May_163	3.4	0.8	11.1	5.6	15.2	63.8	3
K2, 3 May 2022	2022May_164	17.9	1.7	36.3	15.4	8.6	20.2	1
K2, 3 May 2022	2022May_165	20.6	1.7	11.6	24.5	14.0	27.7	2
K2, 3 May 2022	2022May_166	20.1	0.2	28.2	14.1	11.1	26.2	2
K2, 3 May 2022	2022May_167	7.0	0.2	9.3	5.1	17.3	61.0	3
K2, 3 May 2022	2022May_168	0.4	0.2	0.5	2.2	15.7	81.0	3
K2, 3 May 2022	2022May_169	18.4	2.1	25.2	13.7	11.7	28.8	2
K2, 3 May 2022	2022May_170	23.3	2.3	16.8	15.1	15.3	27.2	2
K2, 3 May 2022	2022May_171	15.1	1.1	16.3	14.4	12.1	41.0	2
K2, 3 May 2022	2022May_172	15.7	2.1	38.8	18.6	8.6	16.2	1
K2, 3 May 2022	2022May_173	6.8	0.6	11.2	7.8	15.9	57.7	3
K2, 3 May 2022	2022May_174	15.6	1.7	24.6	13.2	12.1	32.9	2
K2, 3 May 2022	2022May_175	19.7	1.1	28.4	16.9	11.0	22.9	2
K2, 3 May 2022	2022May_176	21.2	2.7	42.8	15.9	5.0	12.4	1
K2, 3 May 2022	2022May_177	20.3	0.0	11.3	13.9	12.7	41.8	2
K2, 3 May 2022	2022May_178	18.7	2.1	42.0	18.6	6.1	12.4	1
K2, 3 May 2022	2022May_179	22.9	1.5	35.2	22.3	6.6	11.4	1
K2, 3 May 2022	2022May_180	18.3	2.9	44.3	15.3	6.7	12.5	1
K2, 3 May 2022	2022May_181	4.4	0.9	13.7	6.8	16.5	57.8	3
K2, 3 May 2022	2022May_182	17.2	2.2	38.4	15.5	10.1	16.5	1
K2, 3 May 2022	2022May_183	18.2	2.2	39.6	16.2	8.1	15.8	1
K2, 3 May 2022	2022May_184	14.2	1.8	30.9	14.2	12.7	26.2	2
K2, 3 May 2022	2022May_185	0.1	0.3	0.0	2.9	21.9	74.8	3
K2, 3 May 2022	2022May_186	17.8	2.4	40.0	16.2	9.1	14.5	1
K2, 3 May 2022	2022May_187	23.6	1.3	30.1	13.3	9.0	22.6	2
K2, 3 May 2022	2022May_188	22.4	1.9	39.5	16.7	6.7	12.8	1

K2, 3 May 2022	2022May_189	19.5	0.6	11.6	11.8	17.4	39.0	2
K2, 3 May 2022	2022May_190	19.3	1.6	39.0	17.7	7.5	15.0	1
K2, 3 May 2022	2022May_191	23.2	2.0	36.8	15.8	7.7	14.4	1
K2, 3 May 2022	2022May_192	20.8	1.9	43.2	17.2	5.8	11.1	1
K2, 3 May 2022	2022May_193	18.5	1.5	16.1	15.9	15.2	32.9	2
K2, 3 May 2022	2022May_194	19.4	1.2	10.8	19.2	19.2	30.3	2
K2, 3 May 2022	2022May_195	18.4	1.9	39.5	17.0	8.1	15.2	1
K2, 3 May 2022	2022May_196	16.4	2.2	38.5	16.7	10.4	15.8	1
K2, 3 May 2022	2022May_197	13.9	1.4	12.1	10.8	18.1	43.6	3
K2, 3 May 2022	2022May_198	19.5	1.7	39.3	16.5	7.7	15.4	1
K2, 3 May 2022	2022May_199	9.8	2.7	24.4	12.2	13.4	37.3	2
K2, 3 May 2022	2022May_200	6.9	1.3	16.4	7.8	16.2	51.5	3
K2, 3 May 2022	2022May_201	22.9	2.3	41.6	13.4	8.4	11.4	1
K2, 3 May 2022	2022May_202	20.0	2.7	38.2	19.7	7.5	11.9	1
K2, 3 May 2022	2022May_203	18.1	2.2	40.5	16.7	8.9	13.6	1
K2, 3 May 2022	2022May_204	1.7	0.7	5.7	4.2	21.5	66.1	3
K2, 3 May 2022	2022May_205	7.9	1.1	17.6	10.1	16.4	47.1	3
K2, 3 May 2022	2022May_206	11.3	2.3	14.4	12.1	18.3	41.7	2
K2, 3 May 2022	2022May_207	3.3	0.9	10.0	5.0	18.7	62.2	3
K2, 3 May 2022	2022May_208	15.4	2.1	36.5	14.4	9.4	22.3	1
K2, 3 May 2022	2022May_209	18.7	1.5	38.6	22.4	6.6	12.2	1
K2, 3 May 2022	2022May_210	0.4	0.4	0.0	3.9	22.8	72.5	3
K2, 3 May 2022	2022May_211	11.1	2.0	24.5	11.6	15.3	35.5	2
K2, 3 May 2022	2022May_212	16.8	2.3	27.5	11.0	12.0	30.4	2
K2, 3 May 2022	2022May_213	17.7	2.6	34.2	11.9	8.7	24.9	2
K2, 3 May 2022	2022May_214	8.8	2.1	12.8	11.2	18.5	46.6	3
K2, 3 May 2022	2022May_215	17.7	1.7	36.9	17.7	9.6	16.5	1
K2, 3 May 2022	2022May_216	10.5	1.6	14.4	12.8	15.7	45.0	3
K2, 3 May 2022	2022May_217	12.8	1.9	19.7	13.6	17.0	35.0	2
K2, 3 May 2022	2022May_218	15.1	2.2	39.5	15.9	8.7	18.6	1
K2, 3 May 2022	2022May_219	21.4	2.7	36.7	12.7	8.7	17.7	1
K2, 3 May 2022	2022May_220	12.3	1.6	13.1	12.5	18.2	42.5	2
K2, 3 May 2022	2022May_221	18.2	1.8	44.5	17.0	6.1	12.4	1
K2, 3 May 2022	2022May_222	1.3	0.6	4.0	4.0	20.2	69.9	3
K2, 3 May 2022	2022May_223	14.6	2.3	38.3	18.2	9.5	17.2	1
K2, 3 May 2022	2022May_224	13.5	1.2	13.3	15.9	19.3	36.7	2
K2, 3 May 2022	2022May_225	26.4	1.3	34.1	11.8	13.3	13.1	1
K2, 3 May 2022	2022May_226	15.2	2.1	38.0	14.7	9.3	20.7	1
K2, 3 May 2022	2022May_227	20.0	2.0	40.0	16.5	9.2	12.3	1
K2, 3 May 2022	2022May_228	17.2	1.5	28.6	14.2	11.4	27.1	2
K2, 3 May 2022	2022May_229	22.6	1.4	31.7	15.4	9.5	19.3	1
K2, 3 May 2022	2022May_230	0.0	2.5	48.2	21.7	9.4	18.2	1
K2, 3 May 2022	2022May_231	0.0	3.1	44.4	17.4	11.1	24.1	2
K2, 3 May 2022	2022May_232	9.5	0.8	17.3	8.4	16.0	47.9	3
K2, 3 May 2022	2022May_233	3.2	0.6	3.6	5.3	23.6	63.7	3
K2, 3 May 2022	2022May_234	20.6	2.1	34.9	14.3	10.4	17.6	1
K2, 3 May 2022	2022May_235	20.4	2.5	41.2	16.6	7.0	12.2	1
K2, 3 May 2022	2022May_236	13.1	2.5	25.3	12.6	15.7	30.8	2
K2, 3 May 2022	2022May_237	15.3	3.4	30.8	9.1	5.8	35.7	2
K2, 3 May 2022	2022May_238	16.0	1.9	32.9	14.6	12.0	22.6	2
K2, 3 May 2022	2022May_239	18.6	1.1	27.3	14.4	12.0	26.6	2
K2, 3 May 2022	2022May_240	20.0	1.7	32.2	16.0	10.4	19.7	1
K2, 3 May 2022	2022May_241	2.7	1.0	9.1	4.6	19.4	63.2	3
K2, 3 May 2022	2022May_242	20.7	2.0	41.5	14.9	8.1	12.7	1
K2, 3 May 2022	2022May_243	15.0	1.3	25.0	12.3	13.4	33.0	2
K2, 3 May 2022	2022May_244	19.1	2.1	36.2	11.7	9.1	21.8	1
K2, 3 May 2022	2022May_245	17.7	1.2	41.1	17.0	7.2	15.7	1
K2, 3 May 2022	2022May_246	15.4	1.7	27.8	14.3	14.2	26.5	2
K2, 3 May 2022	2022May_247	14.1	2.7	14.8	10.8	20.2	37.4	2
K2, 3 May 2022	2022May_248	12.2	1.8	9.4	9.7	20.1	46.9	3
K2, 3 May 2022	2022May_249	18.7	2.2	36.7	13.9	8.8	19.6	1
K2, 3 May 2022	2022May_250	16.8	2.3	34.3	14.0	11.0	21.5	2
K2, 3 May 2022	2022May_251	17.0	2.2	35.5	14.5	10.2	20.6	1
K2, 3 May 2022	2022May_252	14.7	2.1	18.2	10.3	15.9	38.7	2
K2, 3 May 2022	2022May_253	20.5	2.8	36.7	12.2	9.1	18.8	1
K2, 3 May 2022	2022May_254	10.8	2.1	24.7	10.4	13.7	38.2	2
K2, 3 May 2022	2022May_255	27.7	2.0	7.2	6.1	16.6	40.5	2
K2, 3 May 2022	2022May_256	14.4	2.1	24.7	13.4	12.7	32.7	2
K2, 3 May 2022	2022May_257	1.9	0.7	0.0	9.9	31.6	55.8	3
K2, 3 May 2022	2022May_258	18.3	2.0	38.8	16.2	9.1	15.6	1
K2, 3 May 2022	2022May_259	18.6	1.3	28.0	18.6	10.5	22.9	2
K2, 3 May 2022	2022May_260	12.8	1.6	29.3	16.5	13.1	26.7	2
K2, 3 May 2022	2022May_261	14.3	2.3	14.5	16.0	15.5	37.5	2
K2, 3 May 2022	2022May_262	12.6	1.3	20.5	17.8	14.7	33.1	2
K2, 3 May 2022	2022May_263	15.6	1.8	32.3	18.7	9.1	22.4	2
K2, 3 May 2022	2022May_264	17.9	1.9	38.3	18.0	6.9	17.0	1
K2, 3 May 2022	2022May_265	19.2	1.7	46.1	20.4	4.4	8.3	1
K2, 3 May 2022	2022May_266	17.5	1.6	42.1	19.7	6.6	12.4	1
K2, 3 May 2022	2022May_267	12.4	1.2	18.6	15.3	15.6	37.0	2
K2, 3 May 2022	2022May_268	19.8	1.9	20.2	15.1	14.6	28.5	2
K2, 3 May 2022	2022May_269	17.6	0.9	28.7	16.2	11.5	25.2	2
K2, 3 May 2022	2022May_270	18.9	1.2	16.3	16.0	15.1	32.5	2
K2, 3 May 2022	2022May_271	16.0	2.5	42.9	15.7	7.3	15.6	1
K2, 3 May 2022	2022May_272	16.4	1.5	24.8	15.9	12.6	28.8	2
K2, 3 May 2022	2022May_273	11.6	1.8	16.0	12.6	17.7	40.3	2
K2, 3 May 2022	2022May_274	19.2	2.1	25.8	17.2	13.4	22.3	2

K2, 3 May 2022	2022May_275	21.8	1.9	40.4	18.2	5.9	11.8	1
K2, 3 May 2022	2022May_276	25.3	1.5	34.1	15.2	7.9	15.9	1
K2, 3 May 2022	2022May_277	16.7	1.8	27.6	15.5	12.1	26.3	2
K2, 3 May 2022	2022May_278	15.1	1.8	24.8	13.4	12.4	32.5	2
K2, 3 May 2022	2022May_279	25.9	1.4	31.5	19.1	7.6	14.5	1
K2, 3 May 2022	2022May_280	23.7	2.3	42.5	17.7	5.9	7.9	1
K2, 3 May 2022	2022May_281	16.5	0.9	10.0	11.8	19.6	41.2	2
K2, 3 May 2022	2022May_282	23.8	2.0	33.4	16.3	9.0	15.6	1
K2, 3 May 2022	2022May_283	13.1	1.5	16.7	12.1	14.6	42.0	2
K2, 3 May 2022	2022May_284	20.8	2.2	34.0	16.1	8.7	18.2	1
K2, 3 May 2022	2022May_285	18.8	1.9	34.7	17.2	10.9	16.6	1
K2, 3 May 2022	2022May_286	20.4	1.8	37.2	17.2	8.7	14.7	1
K2, 3 May 2022	2022May_287	7.3	0.8	15.6	9.2	13.6	53.6	3
K2, 3 May 2022	2022May_288	20.9	1.7	38.2	17.9	7.8	13.5	1
K2, 3 May 2022	2022May_289	19.2	0.0	6.3	11.0	15.4	48.0	3
K2, 3 May 2022	2022May_290	13.7	1.7	27.0	12.5	11.3	33.7	2
K2, 3 May 2022	2022May_291	14.2	1.1	22.5	13.0	12.1	37.0	2
K2, 3 May 2022	2022May_292	12.8	2.9	34.7	12.9	7.7	29.0	2
K2, 3 May 2022	2022May_293	17.2	0.6	16.4	13.5	15.2	37.1	2
K2, 3 May 2022	2022May_294	0.5	0.2	1.4	2.9	20.4	74.6	3
K2, 3 May 2022	2022May_295	5.3	1.0	14.5	7.4	16.7	55.1	3
K2, 3 May 2022	2022May_296	13.2	1.6	22.9	14.3	13.8	34.3	2
K2, 3 May 2022	2022May_297	16.9	1.9	13.7	10.8	15.2	41.5	2
K2, 3 May 2022	2022May_298	20.9	1.9	35.0	18.0	7.9	16.3	1
K2, 3 May 2022	2022May_299	24.0	0.9	11.9	12.3	17.3	33.6	2
K2, 3 May 2022	2022May_300	15.8	2.1	29.6	15.1	10.4	27.0	2
K2, 3 May 2022	2022May_301	19.2	1.1	39.5	21.0	6.1	13.1	1
K2, 3 May 2022	2022May_302	18.4	1.7	23.8	17.1	10.7	28.3	2
K2, 3 May 2022	2022May_303	19.2	2.1	42.2	18.3	6.1	12.2	1
K2, 3 May 2022	2022May_304	17.1	1.1	26.3	15.6	11.0	28.9	2
K2, 3 May 2022	2022May_305	27.1	0.5	27.9	23.5	7.8	13.1	1
K2, 3 May 2022	2022May_306	21.4	1.1	29.8	19.1	8.4	20.3	2
K2, 3 May 2022	2022May_307	23.2	2.3	31.6	15.4	9.6	18.0	1
K2, 3 May 2022	2022May_308	26.2	1.7	23.1	13.7	12.5	22.7	2
K2, 3 May 2022	2022May_309	18.3	2.4	37.7	17.1	8.0	16.5	1
K2, 3 May 2022	2022May_310	21.0	1.6	20.6	12.9	12.9	31.1	2
K2, 3 May 2022	2022May_311	20.1	1.7	17.7	14.1	14.0	32.3	2
K2, 3 May 2022	2022May_312	0.4	0.0	0.0	5.7	31.3	62.6	3
K2, 3 May 2022	2022May_313	19.3	1.4	34.3	16.1	7.9	21.2	1
K2, 3 May 2022	2022May_314	24.6	1.7	38.0	19.0	5.2	11.6	1
K2, 3 May 2022	2022May_315	12.9	1.1	7.0	10.6	18.6	49.7	3
K2, 3 May 2022	2022May_316	25.0	1.7	35.4	16.6	6.0	15.2	1
K2, 3 May 2022	2022May_317	10.9	2.5	32.1	7.9	13.0	33.7	2
K2, 3 May 2022	2022May_318	14.2	1.8	20.6	15.6	16.5	31.3	2
K2, 3 May 2022	2022May_319	8.2	0.9	11.0	9.4	16.1	54.5	3
K2, 3 May 2022	2022May_320	18.2	1.4	26.9	15.2	9.0	29.3	2
K2, 3 May 2022	2022May_321	10.8	1.3	14.1	12.3	18.3	43.2	2
K2, 3 May 2022	2022May_322	0.0	0.1	0.0	2.0	19.9	78.0	3
K2, 3 May 2022	2022May_323	10.9	1.2	19.0	10.1	13.1	45.7	2
K2, 3 May 2022	2022May_324	17.8	2.3	22.8	15.8	13.4	27.9	2
K2, 3 May 2022	2022May_325	23.8	2.2	39.0	17.3	6.5	11.2	1
K2, 3 May 2022	2022May_326	22.2	1.4	23.9	16.6	12.4	23.5	2
K2, 3 May 2022	2022May_327	21.7	2.4	34.4	17.4	7.8	16.3	1
K2, 3 May 2022	2022May_328	19.6	2.3	34.9	18.2	8.7	16.3	1
K2, 3 May 2022	2022May_329	19.9	2.1	42.9	18.3	6.3	10.5	1
K2, 3 May 2022	2022May_330	22.2	2.2	41.0	17.4	5.5	11.7	1
K2, 3 May 2022	2022May_331	20.4	1.2	43.5	20.4	5.6	8.9	1
K2, 3 May 2022	2022May_332	25.4	1.8	37.1	19.5	6.1	10.2	1
K2, 3 May 2022	2022May_333	18.3	1.2	24.9	12.0	13.4	30.1	2
K2, 3 May 2022	2022May_334	12.7	1.5	29.9	13.7	12.0	30.2	2
K2, 3 May 2022	2022May_335	12.9	1.6	32.9	16.6	10.1	25.9	2
K2, 3 May 2022	2022May_336	19.1	1.4	44.0	18.5	6.0	11.1	1
K2, 3 May 2022	2022May_337	21.1	1.7	32.2	18.8	8.6	17.5	1
K2, 3 May 2022	2022May_338	24.7	2.2	26.8	18.3	10.6	17.4	2
K2, 3 May 2022	2022May_339	29.9	1.7	30.6	20.3	5.4	12.1	1
K2, 3 May 2022	2022May_340	18.7	1.7	31.2	16.2	11.1	21.0	2
K2, 3 May 2022	2022May_341	28.5	0.6	23.5	16.8	10.2	20.4	2
K2, 3 May 2022	2022May_342	14.8	1.0	5.6	13.8	21.0	43.8	3
K2, 3 May 2022	2022May_343	21.8	2.0	31.7	16.4	9.3	18.6	1
K2, 3 May 2022	2022May_344	16.4	1.8	37.5	18.7	9.1	16.5	1
K2, 3 May 2022	2022May_345	15.4	2.2	39.7	16.6	7.2	18.9	1
K2, 3 May 2022	2022May_346	17.2	2.1	27.0	14.2	12.1	27.4	2
K2, 3 May 2022	2022May_347	11.0	1.2	8.8	12.3	18.9	47.8	3
K2, 3 May 2022	2022May_348	20.4	2.0	30.1	17.7	8.5	21.3	2
K2, 3 May 2022	2022May_349	13.8	1.5	32.4	14.0	10.3	27.9	1
K2, 3 May 2022	2022May_350	0.9	0.4	3.3	3.2	19.2	73.0	3
K2, 3 May 2022	2022May_351	16.7	1.5	40.3	19.0	8.2	14.3	1
K2, 3 May 2022	2022May_352	13.2	1.0	17.7	14.2	14.2	39.8	2
K2, 3 May 2022	2022May_353	22.8	0.6	18.2	14.3	15.7	28.4	2
K2, 3 May 2022	2022May_354	19.6	1.1	36.7	19.6	7.2	15.8	1
K2, 3 May 2022	2022May_355	14.6	1.3	16.4	11.6	15.3	40.8	2
K2, 3 May 2022	2022May_356	23.0	1.7	14.6	10.1	13.6	37.0	2
K2, 3 May 2022	2022May_357	5.7	0.8	12.4	6.7	15.7	58.7	3
K2, 3 May 2022	2022May_358	20.2	2.1	20.9	14.9	13.0	29.0	2
K2, 3 May 2022	2022May_359	20.3	1.8	35.8	17.3	8.1	16.7	1
K2, 3 May 2022	2022May_360	16.0	2.0	32.7	14.3	9.6	25.4	2

K2, 3 May 2022	2022May_361	28.0	2.8	17.0	11.2	11.7	29.2	2
K2, 3 May 2022	2022May_362	10.4	1.0	15.2	9.0	18.2	46.1	3
K2, 3 May 2022	2022May_363	21.3	2.6	34.0	18.7	9.8	13.7	1
K2, 3 May 2022	2022May_364	12.3	1.4	9.6	13.5	16.6	46.7	3
K2, 3 May 2022	2022May_365	17.5	2.0	39.5	18.2	8.2	14.5	1
K2, 3 May 2022	2022May_366	9.0	1.2	14.9	8.5	17.1	49.3	3
K2, 3 May 2022	2022May_367	22.3	2.2	29.3	18.4	8.5	19.3	2
K2, 3 May 2022	2022May_368	15.5	0.7	27.9	29.2	9.3	17.4	2
K2, 3 May 2022	2022May_369	14.6	1.4	25.1	14.5	12.8	31.6	2
K2, 3 May 2022	2022May_370	15.0	1.2	23.2	16.7	14.0	30.0	2
K2, 3 May 2022	2022May_371	21.1	1.7	27.3	12.2	11.3	26.4	2
K2, 3 May 2022	2022May_372	0.0	0.7	0.0	2.8	18.3	78.3	3
K2, 3 May 2022	2022May_373	16.2	1.8	34.0	16.4	8.5	23.1	2
K2, 3 May 2022	2022May_374	23.2	1.4	26.0	17.0	10.5	21.8	2
K2, 3 May 2022	2022May_375	5.1	0.7	9.4	6.2	16.9	61.7	3
K2, 3 May 2022	2022May_376	20.4	2.0	38.6	16.3	8.0	14.6	1
K2, 3 May 2022	2022May_377	24.9	2.2	9.9	12.8	17.9	32.3	2
K2, 3 May 2022	2022May_378	24.1	0.6	36.5	18.4	6.7	13.6	1
K2, 3 May 2022	2022May_379	22.8	2.3	37.9	17.2	6.2	13.5	1
K2, 3 May 2022	2022May_380	20.3	2.1	36.0	21.6	7.1	13.0	1
K2, 3 May 2022	2022May_381	0.6	0.5	2.4	3.2	18.6	74.7	3
K2, 3 May 2022	2022May_382	17.5	2.1	35.4	17.0	10.3	17.6	1
K2, 3 May 2022	2022May_383	12.8	1.9	22.5	9.5	11.9	41.4	2
K2, 3 May 2022	2022May_384	16.5	2.3	38.1	18.0	8.3	16.8	1
K2, 3 May 2022	2022May_385	1.0	0.4	3.7	3.5	17.8	73.5	3
K2, 3 May 2022	2022May_386	17.8	2.3	37.8	16.5	9.0	16.6	1
K2, 3 May 2022	2022May_387	25.8	1.8	21.1	13.9	12.2	25.2	2
K2, 3 May 2022	2022May_388	15.8	2.0	12.1	19.0	15.8	35.3	2
K2, 3 May 2022	2022May_389	16.1	1.4	25.2	15.3	11.5	30.5	2
K2, 3 May 2022	2022May_390	5.6	0.6	8.9	6.4	20.1	58.4	3
K2, 3 May 2022	2022May_391	18.3	2.1	35.4	16.4	9.8	18.1	1
K2, 3 May 2022	2022May_392	18.6	1.1	26.3	15.1	11.5	27.4	2
K2, 3 May 2022	2022May_393	18.5	2.2	26.3	14.4	12.3	26.4	2
K2, 3 May 2022	2022May_394	22.1	1.6	21.4	12.4	10.6	31.9	2
K2, 3 May 2022	2022May_395	18.6	2.3	43.5	16.3	6.0	13.3	1
K2, 3 May 2022	2022May_396	17.1	2.1	31.9	14.4	12.7	21.8	2
K2, 3 May 2022	2022May_397	22.7	2.4	36.2	17.0	8.0	13.8	1
K2, 3 May 2022	2022May_398	3.0	0.6	6.4	4.8	20.6	64.6	3
K2, 3 May 2022	2022May_399	19.5	2.3	34.7	16.0	10.0	17.5	1
K2, 3 May 2022	2022May_400	22.4	1.4	32.6	15.1	11.3	17.4	1
K2, 3 May 2022	2022May_401	6.7	0.7	10.9	6.6	16.1	59.0	3
K2, 3 May 2022	2022May_402	18.5	1.8	34.6	15.0	9.6	20.5	1
K2, 3 May 2022	2022May_403	22.6	1.8	38.7	16.1	8.1	12.8	1
K2, 3 May 2022	2022May_404	14.6	2.1	37.9	15.2	10.0	20.2	1
K2, 3 May 2022	2022May_405	27.0	1.6	39.9	15.3	7.1	9.0	1
K2, 3 May 2022	2022May_406	26.6	0.1	18.1	16.3	13.2	25.7	2
K2, 3 May 2022	2022May_407	18.7	2.3	40.7	17.2	7.6	13.5	1
K2, 3 May 2022	2022May_408	19.7	1.9	29.3	16.8	9.9	22.3	2
K2, 3 May 2022	2022May_409	14.4	1.4	17.1	12.2	17.5	37.5	2
K2, 3 May 2022	2022May_410	21.7	2.5	32.3	17.2	8.6	17.6	1
K2, 3 May 2022	2022May_411	22.8	2.3	33.0	17.1	8.8	16.1	1
K2, 3 May 2022	2022May_412	16.9	1.8	38.9	15.4	9.2	17.9	1
K2, 3 May 2022	2022May_413	0.0	0.4	0.0	3.0	20.7	75.9	2
K2, 3 May 2022	2022May_414	19.0	1.0	31.5	15.3	10.5	22.8	2
K2, 3 May 2022	2022May_415	2.5	0.8	0.0	5.8	19.6	71.3	3
K2, 3 May 2022	2022May_416	20.0	1.7	35.9	17.2	8.2	17.0	1
K2, 3 May 2022	2022May_417	28.4	2.4	31.2	16.8	7.7	13.5	1
K2, 3 May 2022	2022May_418	2.2	0.7	7.1	4.2	18.3	67.5	3
K2, 3 May 2022	2022May_419	0.2	0.3	0.0	2.7	24.0	72.7	3
K2, 3 May 2022	2022May_420	21.7	1.7	38.6	20.6	6.9	10.7	1
K2, 3 May 2022	2022May_421	15.9	0.3	13.4	12.3	17.6	40.5	2
K2, 3 May 2022	2022May_422	28.0	1.1	26.7	18.8	8.8	16.7	1
K2, 3 May 2022	2022May_423	22.4	1.8	38.2	19.4	6.0	12.1	1
K2, 3 May 2022	2022May_424	18.5	2.3	30.9	15.6	10.6	22.0	2
K2, 3 May 2022	2022May_425	18.4	2.2	41.2	16.6	7.4	14.3	1
K2, 3 May 2022	2022May_426	17.5	1.8	9.3	15.4	19.5	36.6	2
K2, 3 May 2022	2022May_427	22.0	1.3	27.8	16.6	12.9	19.4	2
K2, 3 May 2022	2022May_428	17.2	1.0	17.8	14.6	15.6	33.8	2
K2, 3 May 2022	2022May_429	8.0	1.3	27.0	15.5	11.4	36.8	2
K2, 3 May 2022	2022May_430	13.9	1.5	32.3	15.7	10.5	26.1	2
K2, 3 May 2022	2022May_431	9.9	1.0	15.0	9.5	16.2	48.5	3
K2, 3 May 2022	2022May_432	14.2	1.4	31.3	16.3	10.0	26.8	2
K2, 3 May 2022	2022May_433	19.2	1.4	41.8	19.4	6.2	12.0	1
K2, 3 May 2022	2022May_434	5.0	0.9	8.6	8.7	15.5	61.4	3
K2, 3 May 2022	2022May_435	17.6	1.7	34.7	15.6	9.5	20.9	1
K2, 3 May 2022	2022May_436	15.9	1.5	38.5	17.8	8.7	17.6	1
K2, 3 May 2022	2022May_437	23.0	1.6	15.6	12.0	15.7	32.1	2
K2, 3 May 2022	2022May_438	19.0	1.7	29.3	15.4	9.7	24.9	2
K2, 3 May 2022	2022May_439	14.4	1.1	25.5	13.8	13.8	31.5	2
K2, 3 May 2022	2022May_440	18.0	2.0	35.4	17.6	8.5	18.6	1
K2, 3 May 2022	2022May_441	16.9	2.1	37.0	18.6	7.6	17.8	1
K2, 3 May 2022	2022May_442	7.2	1.0	17.6	9.0	14.6	50.7	3
K2, 3 May 2022	2022May_443	15.1	1.7	38.9	16.1	8.7	19.5	1
K2, 3 May 2022	2022May_444	11.7	1.4	27.2	11.6	9.4	38.8	2
K2, 3 May 2022	2022May_445	4.2	0.6	12.2	7.9	15.0	60.1	3
K2, 3 May 2022	2022May_446	19.6	2.2	21.3	14.3	11.2	31.5	2

K2, 3 May 2022	2022May_447	3.5	0.3	5.0	4.2	17.1	69.9	3
Nagasaki, Japan, 20–21 March 2010	Dust_1	21.1	2.8	40.0	10.6	10.3	15.2	1
Nagasaki, Japan, 20–21 March 2010	Dust_2	16.1	2.6	23.6	10.2	14.6	33.0	2
Nagasaki, Japan, 20–21 March 2010	Dust_3	25.1	2.7	32.7	12.7	9.2	17.5	1
Nagasaki, Japan, 20–21 March 2010	Dust_4	32.3	0.9	16.0	11.4	15.8	23.6	2
Nagasaki, Japan, 20–21 March 2010	Dust_5	30.2	2.7	38.8	12.9	6.8	8.6	1
Nagasaki, Japan, 20–21 March 2010	Dust_6	24.7	2.9	31.6	14.2	9.8	16.9	1
Nagasaki, Japan, 20–21 March 2010	Dust_7	27.4	0.5	8.3	7.7	22.4	33.7	2
Nagasaki, Japan, 20–21 March 2010	Dust_8	26.8	3.0	46.3	12.0	5.7	6.3	1
Nagasaki, Japan, 20–21 March 2010	Dust_9	22.7	3.2	19.7	13.7	17.4	23.2	2
Nagasaki, Japan, 20–21 March 2010	Dust_10	19.9	2.7	34.0	10.8	9.1	23.6	2
Nagasaki, Japan, 20–21 March 2010	Dust_11	22.3	3.1	32.7	11.6	11.3	19.1	1
Nagasaki, Japan, 20–21 March 2010	Dust_12	25.1	1.6	39.3	17.1	4.2	12.8	1
Nagasaki, Japan, 20–21 March 2010	Dust_13	20.8	1.9	31.4	11.6	10.5	23.8	2
Nagasaki, Japan, 20–21 March 2010	Dust_14	22.6	2.6	39.4	11.8	8.8	14.7	1
Nagasaki, Japan, 20–21 March 2010	Dust_15	39.0	3.9	23.7	13.5	8.9	11.0	1
Nagasaki, Japan, 20–21 March 2010	Dust_16	22.6	3.6	31.7	11.4	7.9	22.8	2
Nagasaki, Japan, 20–21 March 2010	Dust_17	24.9	3.6	32.0	14.9	9.9	14.8	1
Nagasaki, Japan, 20–21 March 2010	Dust_18	23.3	2.3	46.5	15.2	4.9	7.8	1
Nagasaki, Japan, 20–21 March 2010	Dust_19	22.6	2.9	22.7	14.3	17.2	20.3	2
Nagasaki, Japan, 20–21 March 2010	Dust_20	21.3	3.0	20.1	13.5	15.6	26.5	2
Nagasaki, Japan, 20–21 March 2010	Dust_21	0.6	1.6	1.7	4.0	29.1	63.0	3
Nagasaki, Japan, 20–21 March 2010	Dust_22	23.6	3.1	31.1	11.3	10.9	19.9	1
Nagasaki, Japan, 20–21 March 2010	Dust_23	27.7	3.0	40.0	11.2	7.0	11.1	1
Nagasaki, Japan, 20–21 March 2010	Dust_24	26.9	3.5	34.6	13.0	12.1	9.9	1
Nagasaki, Japan, 20–21 March 2010	Dust_25	20.5	2.5	21.4	10.9	17.0	27.6	2
Nagasaki, Japan, 20–21 March 2010	Dust_26	26.0	4.3	30.6	14.1	10.8	14.4	1
Nagasaki, Japan, 20–21 March 2010	Dust_27	24.2	2.1	36.7	11.9	9.8	15.2	1
Nagasaki, Japan, 20–21 March 2010	Dust_28	28.7	2.1	41.9	13.6	5.4	8.3	1
Nagasaki, Japan, 20–21 March 2010	Dust_29	20.8	3.3	43.3	11.3	8.6	12.6	1
Nagasaki, Japan, 20–21 March 2010	Dust_30	25.0	2.5	42.2	15.0	5.9	9.4	1
Nagasaki, Japan, 20–21 March 2010	Dust_31	13.9	2.4	14.0	11.9	22.7	35.2	2
Nagasaki, Japan, 20–21 March 2010	Dust_32	19.8	2.1	35.1	10.8	10.8	21.3	1
Nagasaki, Japan, 20–21 March 2010	Dust_33	24.7	0.0	0.0	8.1	23.9	43.3	3
Nagasaki, Japan, 20–21 March 2010	Dust_34	16.7	2.5	34.0	8.4	13.6	24.9	2
Nagasaki, Japan, 20–21 March 2010	Dust_35	26.6	3.0	35.1	13.6	9.5	12.3	1
Nagasaki, Japan, 20–21 March 2010	Dust_36	30.9	0.3	27.8	10.9	12.7	17.3	1
Nagasaki, Japan, 20–21 March 2010	Dust_37	30.1	3.0	38.0	13.5	6.8	8.7	1
Nagasaki, Japan, 20–21 March 2010	Dust_38	32.1	0.0	25.2	13.0	11.9	17.8	2
Nagasaki, Japan, 20–21 March 2010	Dust_39	25.0	2.0	34.3	13.1	9.2	16.4	1
Nagasaki, Japan, 20–21 March 2010	Dust_40	22.0	2.8	36.9	12.5	11.8	14.0	1
Nagasaki, Japan, 20–21 March 2010	Dust_41	25.2	1.0	19.1	11.3	15.4	28.1	2
Nagasaki, Japan, 20–21 March 2010	Dust_42	26.1	2.8	41.9	14.0	6.1	9.1	1
Nagasaki, Japan, 20–21 March 2010	Dust_43	22.7	3.1	43.6	12.7	7.3	10.6	1
Nagasaki, Japan, 20–21 March 2010	Dust_44	31.4	0.0	20.0	9.6	17.1	21.9	2
Nagasaki, Japan, 20–21 March 2010	Dust_45	28.3	1.3	29.2	11.5	12.1	17.6	1
Nagasaki, Japan, 20–21 March 2010	Dust_46	21.0	2.7	38.1	13.5	8.9	15.7	1
Nagasaki, Japan, 20–21 March 2010	Dust_47	26.7	0.3	15.7	8.3	20.8	28.3	2
Nagasaki, Japan, 20–21 March 2010	Dust_48	30.7	0.0	27.8	10.5	12.9	18.0	1
Nagasaki, Japan, 20–21 March 2010	Dust_49	24.6	0.0	0.0	4.4	29.0	42.0	3
Nagasaki, Japan, 20–21 March 2010	Dust_50	10.6	0.3	8.6	6.5	23.1	51.0	3
Nagasaki, Japan, 20–21 March 2010	Dust_51	28.2	1.5	41.1	12.6	7.0	9.5	1
Nagasaki, Japan, 20–21 March 2010	Dust_52	28.5	2.1	35.6	14.8	5.8	13.3	1
Nagasaki, Japan, 20–21 March 2010	Dust_53	24.7	4.1	38.1	13.4	9.0	10.8	1
Nagasaki, Japan, 20–21 March 2010	Dust_54	15.3	1.7	15.3	11.1	21.9	34.8	2
Nagasaki, Japan, 20–21 March 2010	Dust_55	27.8	1.4	22.2	11.6	13.5	23.4	2
Nagasaki, Japan, 20–21 March 2010	Dust_56	30.5	2.3	43.0	13.7	3.7	6.7	1
Nagasaki, Japan, 20–21 March 2010	Dust_57	8.6	2.3	20.5	5.2	10.5	52.9	3
Nagasaki, Japan, 20–21 March 2010	Dust_58	17.6	2.2	19.7	11.4	18.4	30.8	2
Nagasaki, Japan, 20–21 March 2010	Dust_59	22.7	2.8	23.1	11.2	13.9	26.2	2
Nagasaki, Japan, 20–21 March 2010	Dust_60	22.5	1.9	20.4	11.9	17.1	26.2	2
Nagasaki, Japan, 20–21 March 2010	Dust_61	28.3	2.2	38.2	16.7	6.7	7.9	1
Nagasaki, Japan, 20–21 March 2010	Dust_62	20.7	2.8	33.9	14.0	12.1	16.6	1
Nagasaki, Japan, 20–21 March 2010	Dust_63	26.8	2.7	43.1	14.1	5.2	8.1	1
Nagasaki, Japan, 20–21 March 2010	Dust_64	8.7	2.5	19.1	8.8	22.0	38.8	2
Nagasaki, Japan, 20–21 March 2010	Dust_65	24.7	2.6	33.2	14.3	8.7	16.4	1
Nagasaki, Japan, 20–21 March 2010	Dust_66	4.7	3.1	22.9	6.9	23.0	39.4	2
Nagasaki, Japan, 20–21 March 2010	Dust_67	27.3	2.6	38.8	12.6	7.9	10.7	1
Nagasaki, Japan, 20–21 March 2010	Dust_68	27.6	2.8	44.7	12.8	5.2	6.9	1
Nagasaki, Japan, 20–21 March 2010	Dust_69	24.0	3.2	37.8	11.3	9.9	13.8	1
Nagasaki, Japan, 20–21 March 2010	Dust_70	18.4	2.6	32.0	11.3	15.8	19.9	2
Nagasaki, Japan, 20–21 March 2010	Dust_71	13.5	4.5	14.0	9.7	20.6	37.7	2
Nagasaki, Japan, 20–21 March 2010	Dust_72	35.0	3.3	28.5	14.5	9.0	9.8	1
Nagasaki, Japan, 20–21 March 2010	Dust_73	28.0	3.1	44.5	12.4	4.7	7.2	1

Table S7. Dust deposition flux and dissolved iron flux calculated using K2 samples.

Station, sampling day	Dust deposition flux ± σ (mg m^{-2} day^{-1})	Dissolved iron flux ± σ (µg m^{-2} day^{-1})
K2, 21 February 2021	0.33 ± 0.14	0.21 ± 0.12
K2, 25 February 2003	0.00 ± 0.04	0.00 ± 0.03
K2, 8 April 2004	0.39 ± 0.11	0.25 ± 0.12
K2, 3 May 2022	6.26 ± 1.74	4.04 ± 1.83
K2, 27 May 2004	0.07 ± 0.05	0.05 ± 0.04
K2, 13 June 2006	1.91 ± 0.48	1.23 ± 0.54
K2, 30 June 2006	0.66 ± 0.33	0.42 ± 0.26
K2, 9 July 2006	0.70 ± 0.34	0.45 ± 0.27
K2, 11 July 2003	0.26 ± 0.10	0.17 ± 0.09
K2, 17 August 2004	0.34 ± 0.14	0.22 ± 0.12
K2, 4 October 2003	0.30 ± 0.09	0.20 ± 0.09

Table S8. Monthly deposition flux (wet and dry depositions) of Asian dust at station K2 averaged for 2001–2020 simulated using MIROC-SPRINTARS.

Month	Total deposition flux (mg m^{-2} day^{-1})	
	average	± σ
1	0.24	0.22
2	0.59	0.39
3	1.93	1.26
4	2.77	1.33
5	2.70	1.37
6	1.37	0.69
7	0.69	0.28
8	0.71	0.36
9	0.78	0.26
10	0.81	0.27
11	0.56	0.25
12	0.33	0.25

Table S9. Calculated dissolved iron amounts transported by winter mixing and the upward flux by advection and diffusion at station K2.

	KH-12-4, BD-7
Winter maximum MLD*, m	120 ± 10
Summer MLD, m	10
dFe/dZ, µmol m^{-4}	0.0036
Dissolved iron concentration at winter MLD in the summer vertical profile, µmol m^{-3}: C1	0.45 ± 0.05
Dissolved iron concentration in summer MLD, µmol m^{-3}: C2	0.12
Mean dissolved iron concentration at subsurface gradient, µmol m^{-3}: R	0.52
Dissolved iron amounts transported by winter mixing, µg m^{-2}	180 ± 30
Dissolved iron amounts transported by winter mixing divided by 120 days, µg m^{-2} day^{-1}	1.5 ± 0.2
Dissolved iron flux transported by eddy diffusion, µg m^{-2} day^{-1}	0.40
Dissolved iron flux transported by vertical advection, µg m^{-2} day^{-1}	0.35
Dissolved iron flux transported by eddy diffusion plus vertical advectiond, µg m-2 day-1	0.73
Reference of dissolved iron concentration data	Nishioka and Obata, 2017[1]

*Employed winter maximum mixed layer depth (MLD) of Ref. (9).
